# Pluripotency-independent induction of human trophoblast stem cells from fibroblasts

**DOI:** 10.1038/s41467-023-39104-1

**Published:** 2023-06-08

**Authors:** Moriyah Naama, Moran Rahamim, Valery Zayat, Shulamit Sebban, Ahmed Radwan, Dana Orzech, Rachel Lasry, Annael Ifrah, Mohammad Jaber, Ofra Sabag, Hazar Yassen, Areej Khatib, Silvina Epsztejn-Litman, Michal Novoselsky-Persky, Kirill Makedonski, Noy Deri, Debra Goldman-Wohl, Howard Cedar, Simcha Yagel, Rachel Eiges, Yosef Buganim

**Affiliations:** 1 Department of Developmental Biology and Cancer Research, The Institute for Medical Research Israel-Canada, The Hebrew University-Hadassah Medical School, 91120 Jerusalem, Israel; 2 Department of Stem Cell Bioengineering, Mossakowski Medical Research Institute, Polish Academy of Sciences, Warsaw, 02-106 Poland; 3 Stem Cell Research Laboratory, Medical Genetics Institute, Shaare Zedek Medical Center, 91031 Jerusalem, Israel; 4 The Hebrew University School of Medicine, 91120 Jerusalem, Israel; 5 The Magda and Richard Hoffman Laboratory of Human Placental Research, Department of Obstetrics and Gynecology, Hadassah-Hebrew University Medical Center, Jerusalem, Israel

**Keywords:** Reprogramming, Multipotent stem cells, Stem-cell differentiation

## Abstract

Human trophoblast stem cells (hTSCs) can be derived from embryonic stem cells (hESCs) or be induced from somatic cells by OCT4, SOX2, KLF4 and MYC (OSKM). Here we explore whether the hTSC state can be induced independently of pluripotency, and what are the mechanisms underlying its acquisition. We identify GATA3, OCT4, KLF4 and MYC (GOKM) as a combination of factors that can generate functional hiTSCs from fibroblasts. Transcriptomic analysis of stable GOKM- and OSKM-hiTSCs reveals 94 hTSC-specific genes that are aberrant specifically in OSKM-derived hiTSCs. Through time-course-RNA-seq analysis, H3K4me2 deposition and chromatin accessibility, we demonstrate that GOKM exert greater chromatin opening activity than OSKM. While GOKM primarily target hTSC-specific loci, OSKM mainly induce the hTSC state via targeting hESC and hTSC shared loci. Finally, we show that GOKM efficiently generate hiTSCs from fibroblasts that harbor knockout for pluripotency genes, further emphasizing that pluripotency is dispensable for hTSC state acquisition.

## Abstract (web summary)

In this work Naama et al. describe and deeply characterize a direct approach to produce human induced trophoblast stem cells from fibroblasts and show that it produces superior hiTSCs when compared to OSKM-hiTSCs.

## Introduction

For an extensive period of time, all attempts to isolate and propagate human trophoblast stem cells (hTSCs) in vitro had failed due to lack of knowledge of the culture conditions required for the maintenance of these cells. Recently, such culture conditions were identified and for the first time hTSCs were successfully derived and propagated from blastocysts and first trimester placentas^1^. Following differentiation, these hTSCs gave rise to all major trophoblast cell types, exhibited transcriptional and epigenetic signatures similar to first trimester cytotrophoblasts and formed trophoblastic lesions when injected into NOD/SCID mice, suggesting fully functional hTSCs^1^.

However, this method did not allow the derivation of hTSCs from disease-affected term placentas^1^. Given that placental disorders are detected only at late stages of pregnancy, this constraint largely restricted the usefulness of these cells in modeling placental pathologies and identifying risk factors at early stages of implantation. Alternatively, the ability to convert fibroblasts into other cell types^2^ by a defined number of transcription factors opens an attractive avenue which resolves this limitation, as mesenchymal cells can be isolated relatively easily from post-gestational tissue, such as term placenta, cord blood or skin biopsy following disease-affected pregnancies.

Recent studies demonstrated that human induced trophoblast stem cells (hiTSCs) can be generated either by transdifferentiating human pluripotent cells^3–8^ or by forced expression of OCT4, SOX2, KLF4 and MYC (OSKM) in fibroblasts^3,9^. In these approaches the efficiency and quality of the cells were dependent on the initial acquisition of a pluripotent state in the case of embryonic stem cells (hESCs)/induced pluripotent stem cells (iPSCs) or by a combination of factors (i.e., OSKM) known to robustly activate pluripotency. Although it has been suggested by Liu et al. that OSKM produce a side population of hiTSCs through an alternate reprogramming trajectory, it is still unknown whether these cells acquire a transient pluripotency state before acquisition of hTSC-like identity^9^. Thus, it is still unclear whether the human TSC state can be directly induced from somatic cells and which are the major factors that can mediate this process. Of note, we and others have shown in the mouse system that the direct conversion of fibroblasts into iTSCs is superior to transdifferentiation from pluripotent cells, as the latter was shown to generate unstable colonies with epigenetic abnormalities^10–13^.

Here, we developed a new paradigm in which human fibroblasts are directly converted into hiTSCs by transient upregulation of GATA3, OCT4, KLF4 and MYC. The resultant hiTSCs remain stable and proliferative for many passages, differentiate into the various trophoblast cell types, generate trophoblastic lesions when injected into NOD/SCID mice, retain normal karyotype and form functional trophoblastic organoids. Transcriptional and methylation analyses indicate that hiTSCs closely resemble human blastocyst-derived TSCs (hbdTSCs).

In order to elucidate mechanisms of acquisition of hTSC fate, we profiled the transcriptome and chromatin accessibility and activity of GOKM and OSKM at an early stage of reprogramming. Our results indicate that cells transduced with GOKM follow a distinct route toward the hTSC state compared with OSKM. While GOKM specifically target hTSC *loci*, OSKM induce the hTSC state by targeting regions that are shared between hESCs and hTSCs. Compared with GOKM-iTSCs and blastocyst-derived iTSCs, stable OSKM-hiTSCs lack expression of a signature of 94 genes that are associated with response to estrogen, which may have important implications for disease modeling. Time-course transcriptomic analysis demonstrates unique gene expression patterns for OSKM and GOKM-induced cells during reprogramming, and illuminates the mechanisms by which GATA3 and SOX2 target distinct genetic regions toward the induction of pluripotency or the hTSC state. Finally, we show that the hTSC state achieved by GOKM is acquired independently of pluripotency, and that major pluripotency factors such as SOX2, NANOG, and PRDM14 are dispensable to hTSC fate acquisition. These data suggest that somatic cells can undergo direct lineage conversion into fully functional hiTSCs, and propose a comparative mechanism by which the GOKM and OSKM transcription factors target the genome to acquire the hTSC state.

## Results

### GATA3, OCT4, KLF4 and MYC produce hTSC-like colonies

Previously, we and others have shown that transient ectopic expression of four mouse key trophectoderm (TE) genes, *Gata3*, *Eomes*, *Tfap2c* and *Myc/Ets2* can force fibroblasts to become stable and fully functional mouse induced trophoblast stem cells (miTSCs^10,13^). However, current knowledge suggests that key TE genes vary significantly between human and mouse. Single-cell RNA-seq studies of the human pre-implantation blastocyst revealed that key mouse TE genes such as *Eomes* and *Elf5* are absent or expressed at very low levels in the human TE^14,15^. *Esrrb*, which is expressed in the mouse epiblast^16^ and which plays an important role in the maintenance and induction of pluripotency^11,17,18^ and mTSCs^19^, is not expressed in the human epiblast, but rather in the TE and primitive endoderm (PE)^14,15^. Another crucial difference between mouse and human blastocysts is the involvement of pluripotency genes such as the key master regulator OCT4 in the establishment of the human TE compartment^20^.

**Fig. 1:**
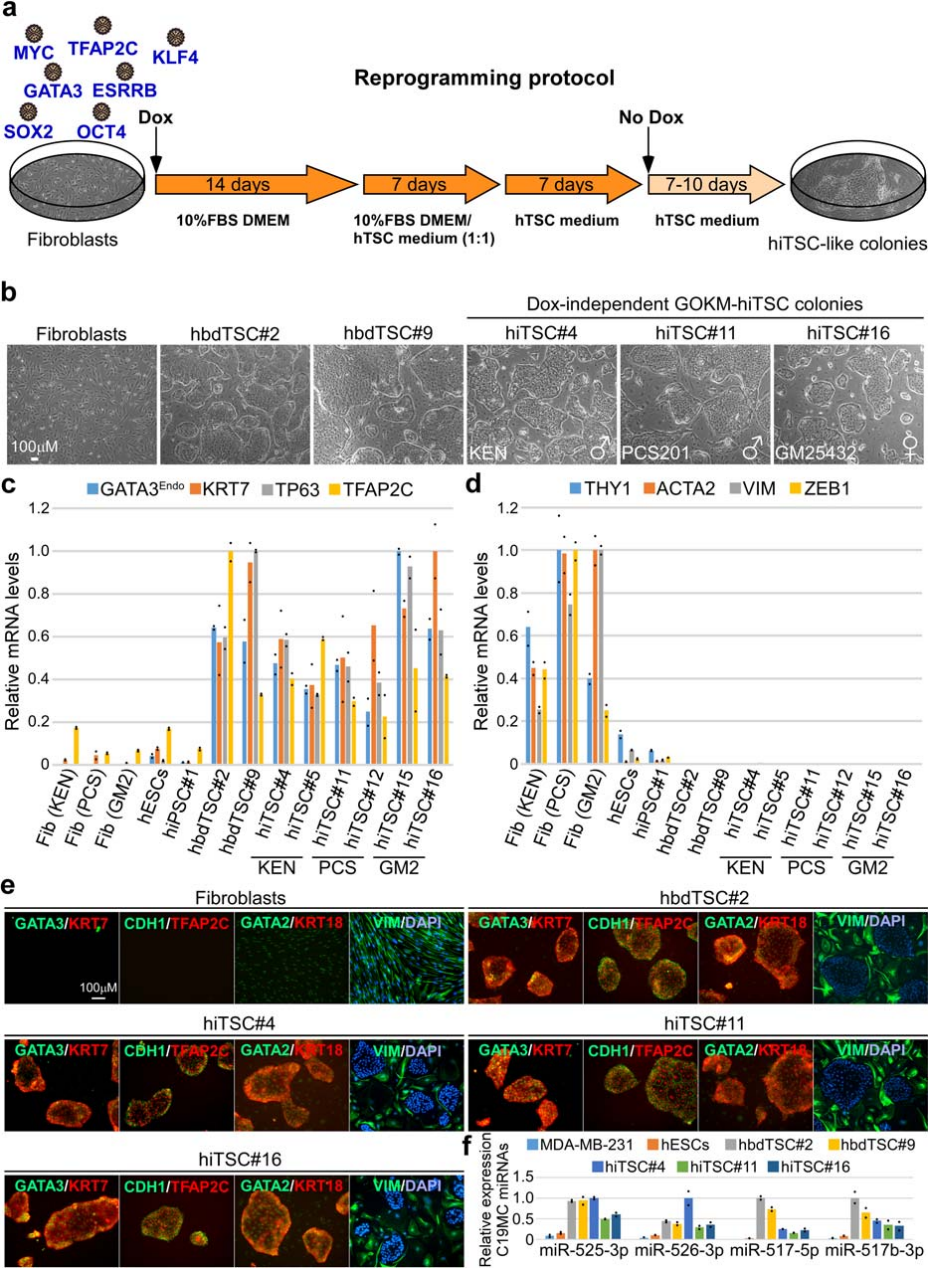
Ectopic expression of GATA3, OCT4, KLF4 and MYC (GOKM) convert human fibroblasts into trophoblast stem-like cells. **a** Schematic representation of the protocol for reprogramming human fibroblasts into human induced trophoblast stem cells (hiTSCs). **b** Bright field images of human primary fibroblasts, two human blastocyst-derived TSC lines, hbdTSC#2 and hbdTSC#9, and three representative GOKM-derived hiTSC colonies (passage #8–11) originating either from KEN (foreskin fibroblasts, hiTSC#4), PCS201 (foreskin fibroblasts, hiTSC#11) or GM25432 (female adult fibroblasts, hiTSC#16) from three independent reprogramming experiments (*n* = 3). **c**, **d** qPCR analysis of mRNA levels for TSC-specific markers *GATA3 (endogenous 5’ UTR expression), KRT7*, *TP63* and *TFAP2C* (**c**) and mesenchymal-specific markers *THY1*, *ACTA2*, *VIM* and *ZEB1* (**d**) in six hiTSC colonies, two hbdTSC lines, three fibroblast lines, hESCs and iPSCs. The indicated hiTSC colonies were derived from three independent reprogramming experiments (*n* = 3). The highest sample for each gene was set to 1. Results were normalized to the mRNA levels of the housekeeping control gene *GAPDH* and are shown as fold change. For each sample two replicates were used (*n* = 2). **e** Immunofluorescence staining for TSC-specific markers GATA3, KRT7, TFAP2C, GATA2, epithelial markers CDH1 and KRT18, and the mesenchymal marker VIM in parental fibroblasts (KEN) and hbdTSC (hbdTSC#2) controls and in three independent (*n* = 3) hiTSC clones, hiTSC#4, hiTSC#11 and hiTSC#16. **f** qPCR analysis for the expression of *C19MC miRNA cluster* in the indicated samples. hiTSC colonies were derived from three independent reprogramming experiments (*n* = 3). The highest sample for each *miR* was set to 1. Results were normalized to the expression levels of the control *miR 103a* and are shown as fold change. For each sample two replicates were used (*n* = 2). See also Supplementary Fig. 1. Source data are provided as a Source Data file.

Thus, in order to reprogram fibroblasts into human induced trophoblast stem cells (hiTSCs), we selected transcription factors with a known role in the development of the human trophoblast lineage but also included pluripotent genes based on their suspected necessity for the induction of the hTSC state^20–22^. In total, we cloned seven genes, *GATA3*, *TFAP2C*, *ESRRB*, *OCT4*, *KLF4*, *SOX2* and MYC, into doxycycline (dox)-inducible lentiviral vectors and used them to infect human foreskin fibroblasts (HFFs). Cells were kept in low (5%) oxygen conditions and treated with dox for 2 weeks in basic reprogramming medium (DMEM + 10%FBS) which was gradually switched to hTSC medium^1^ (Fig. 1a). Following 4 weeks of reprogramming, the induced cells were weaned off dox and allowed to stabilize for 7–10 days, after which individual epithelial-like colonies were manually transferred into separate plates for propagation and analysis. Transgene integration analysis revealed that *GATA3*, *OCT4*, *KLF4* and *MYC* (GOKM) were the only transgenes which had been integrated in all examined colonies (Supplementary Fig. 1a), suggesting that the pluripotent gene *SOX2* is not required for the induction of the TSC state.

Indeed, transduction of GOKM into two primary HFF lines, namely KEN and PCS201, and one primary adult female patient-derived fibroblast line (GM2), produced stable and transgene-independent epithelial-like colonies that maintained their proliferation capacity and hTSC morphology following passaging on mouse feeder cells (Fig. 1b and Supplementary Fig. 1b, c). The reprogramming efficacy of GOKM ranged between 2 × 10^−6^–5 × 10^−5^, depending on the origin and age of the parental fibroblasts, yielding ~5–100 colonies out of 2 × 10^6^ seeded cells in a 10 cm plate (Supplementary Fig. 1d). Importantly, reprogramming with OKM alone yielded no hiTSC colonies (Supplementary Fig. 1d).

In order to evaluate the identity of the resultant colonies, expression of hTSC markers was assessed. Quantitative PCR (qPCR) revealed active transcription of known trophoblast markers such as *GATA2*, *TFAP2A*, *TFAP2C*, *KRT7* and *TP63*, as well as endogenous expression of *GATA3*, in a manner which is comparable to hbdTSCs (Fig. 1c and Supplementary Fig. 1e). As expected, the resultant hiTSC colonies showed drastic downregulation of mesenchymal markers and upregulation of epithelial markers, indicating successful mesenchymal-to-epithelial transition (MET) (Fig. 1d and Supplementary Fig. 1f). Of note, the epithelial marker *KRT18* discriminated between human epithelial cells from trophectodermal origin (i.e., hbdTSCs and hiTSCs), in which the expression was high, and epithelial cells from a pluripotency origin (i.e., ESCs and iPSCs), similar to mouse cells^10^.

Human trophoblast is known to have a unique expression pattern of HLA proteins, including a lack of all HLA class I molecules in villous trophoblast and lack of HLA-A expression in all known trophoblast subtypes^23^. Accordingly, expression of the HLA class I gene *HLA-A* was absent from all hiTSC and hbdTSC lines (Supplementary Fig. 1g^24^).

Expression of hTSC markers GATA3, GATA2, TFAP2C and KRT7, epithelial markers CDH1 and KRT18, as well as the absence of the mesenchymal marker VIM and classical HLA class I proteins (HLA-A/B/C) were validated at the protein level as well (Fig. 1e and Supplementary Fig. 1h). Finally, the TSC-specific C19MC miRNA cluster was highly expressed in all hiTSC colonies and hbdTSC positive controls, but not in hESCs or the breast cancer cell line MDA-MB-231 negative controls (Fig. 1f). Of note, although highly expressed in all hiTSC lines, miR-517-5p demonstrated somewhat reduced levels in hiTSCs compared to hbdTSCs. Taken together, these data suggest that transient GOKM expression can force human fibroblasts to become stable, dox-independent epithelial colonies resembling hbdTSCs in their morphology and hTSC marker expression.

### The transcriptome of hiTSCs is highly similar to hbdTSCs

**Fig. 2:**
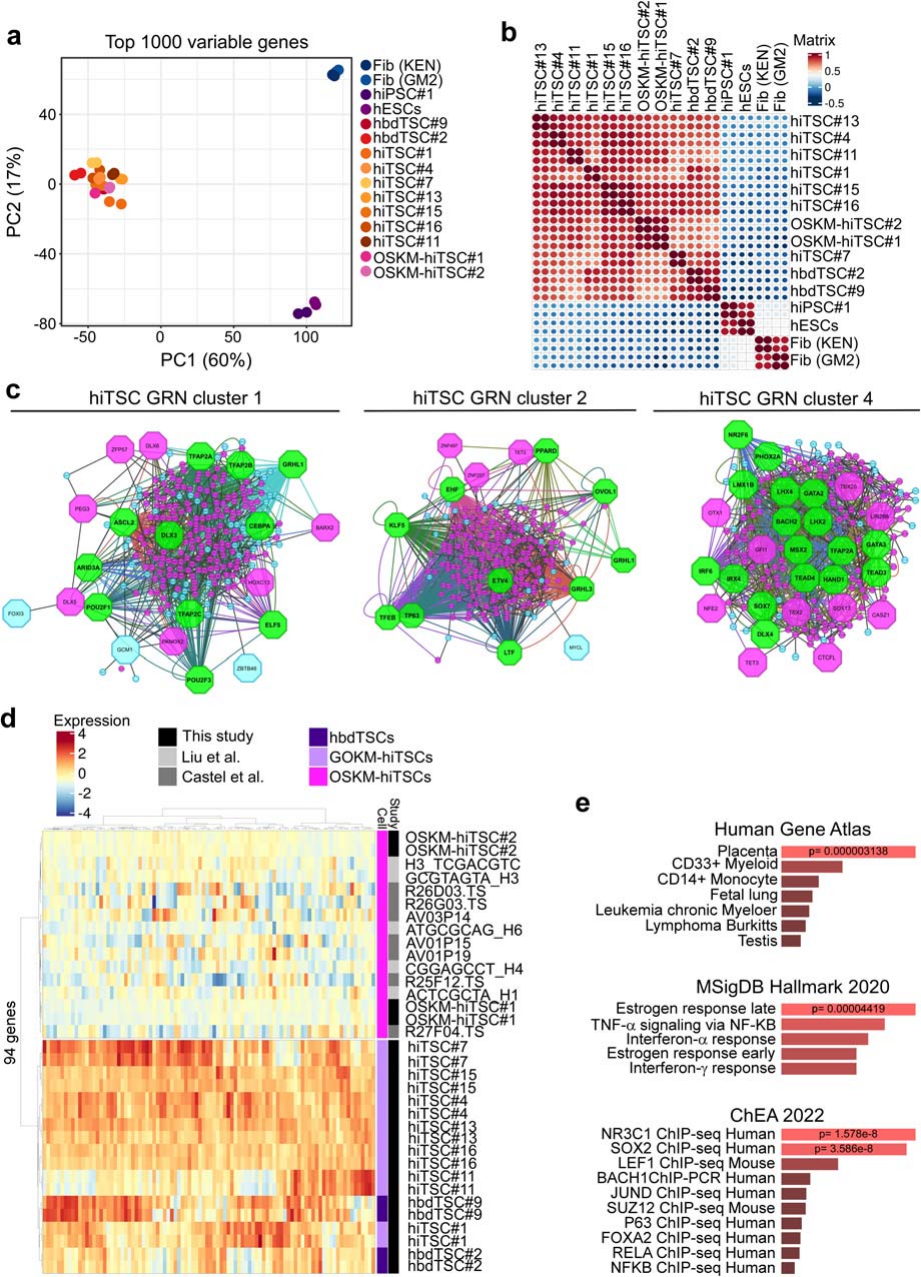
RNA-seq analysis indicates that GOKM-derived hiTSCs exhibit a transcriptome that is highly similar to that of hbdTSCs. **a**, **b** Plots based on RNA-seq data portraying comparisons of whole transcriptome of two biological duplicates of two lines of parental fibroblasts (KEN and GM2), two pluripotent stem cell clones, hESCs and hiPSCs, two hbdTSC lines, hbdTSC#2 and hbdTSC#9, seven independent (*n* = 7) GOKM-derived hiTSC clones, hiTSC#1, hiTSC#4, hiTSC#7, hiTSC#11, hiTSC#13, hiTSC#15 and hiTSC#16, and two OSKM-derived hiTSC clones, OSKM-hiTSC#1 and OSKM-hiTSC#2. Principal component analysis (PCA) plot (**a**) constructed using the top 1000 variable genes, and correlation heatmap (**b**) of bulk RNA displaying the transcriptional similarity between hbdTSCs and hiTSCs and their dissimilarity from PSCs (ESCs and iPSCs) and fibroblasts (KEN and GM2). **c** Network analysis for top 2000 upregulated genes in hiTSCs vs. fibroblasts. Protein-protein interaction network was analyzed with STRING (http://www.string-db.org). The MCODE plugin tool in Cytoscape was used for further analysis of densely connected genes. For each subnetwork we used iRegulon plugin tool in Cytoscape to systematically analyze the composition of the gene promoters in transcription factor binding sites. **d** Heatmap and hierarchical clustering for 94 genes that were found to be differentially expressed in OSKM-derived hiTSCs (in our study and in other studies^3,9^) when compared to hbdTSCs and GOKM-derived hiTSC clones. Importantly, a reciprocal analysis searching for differentially expressed genes in GOKM-hiTSCs identified only one gene (SYK) that is aberrantly expressed in GOKM-hiTSCs. **e** Bar graphs showing the most enriched GO terms, and their *p* value, for the 94 genes from (**d**) using different GO term categories within EnrichR. *p* value was calculated using Fisher exact test. See also Supplementary Fig. 2. Source data are provided as a Source Data file.

Extensive epigenetic reprogramming during somatic cell conversion should ideally result in the activation of a newly established endogenous gene expression circuitry of the targeted cells^25,26^. Incomplete activation of the endogenous circuitry will lead to a partially analogous transcriptome, as can be seen in several direct conversion models^25^. To assess whether hiTSCs successfully activated the endogenous TSC circuitry, we subjected seven hiTSC clones (hiTSC#1, hiTSC#4, hiTSC#7, hiTSC#11, hiTSC#13, hiTSC#15, hiTSC#16) to RNA-sequencing (RNA-seq) analysis. Two hbdTSC clones (hbdTSC#2 and hbdTSC#9), two parental primary fibroblast lines (KEN and GM2) and two pluripotent stem cell (PSC) clones (hESCs and hiPSC#1) were used as positive and negative controls, respectively. As OSKM factors were recently shown to be capable of producing hiTSCs as well^3,9^, we sought to understand whether the selection of factor combination has any effect on gene expression in the resulting hiTSCs. To that end, we reprogrammed fibroblasts into hiTSCs using the OSKM factors and profiled the transcriptome of two OSKM-hiTSC clones (OSKM-hiTSC#1, OSKM-hiTSC#2). Notably, the various hiTSC clones clustered together with hbdTSC clones and far away from the fibroblasts and hESC/hiPSC controls, as indicated by principal component analysis (PCA, Fig. 2a) and hierarchical correlation heatmap (Fig. 2b).

Scatter plot analysis indicated a highly similar transcriptome between hbdTSCs and hiTSCs with R^2^ scores (Pearson coefficient of determination) ranging between 0.89-0.94, and key hTSC genes such as *TP63* and *GATA3* showing high levels of expression in all TSC samples but not in hESC or fibroblast negative controls (Supplementary Fig. 2a). The ~1000 most differentially expressed genes between GOKM-hiTSCs and fibroblasts revealed significant enrichment for gene ontology (GO) terms associated with “trophoblast”, “cell-cell adhesion” and “estrogen response”, and identified TSC factors such as TP63, SUZ12 and GATA family genes as key transcription factors that regulate this gene list according to EnrichR^27^ database (Supplementary Fig. 2b–e). Moreover, using STRING and iRegulon for top 2000 differentially expressed genes between GOKM-hiTSC and fibroblasts we identified gene regulatory networks (GRNs) and protein-protein interactions that are highly associated with the hTSC state^22,23,28^. Among the key regulators of these GRNs are TP63, GATA2, GRHL3, TFAP2A, TFAP2C, ARIDA3, ELF5, TEAD4, KLF5, ETV4, ASCL2, HAND1 and many others (Fig. 2c, marked by green octagons).

To test whether small differences in gene expression exist between hiTSC clones and hbdTSCs, we compared the transcriptome of OSKM-hiTSCs and GOKM-hiTSCs to hbdTSCs. While the comparison between GOKM-hiTSC clones to hbdTSCs identified only one gene (i.e., SYK) that was aberrantly expressed (i.e., low expression) in all GOKM-hiTSC clones, the comparison between OSKM-hiTSC clones to hbdTSCs identified 94 genes with significantly reduced expression in all OSKM-hiTSC clones (*p* value < 0.01), but not in GOKM-hiTSC clones. These 94 genes were similarly absent or mildly expressed in OSKM-hiTSC clones derived in other studies^3,9^, suggesting an intrinsic defect in OSKM-hiTSCs (Fig. 2d). GO term analysis revealed that this group of genes is significantly enriched for “Placenta” and for “early and late response to estrogen”, as well as terms related to TNF-alpha and interferon signaling (Fig. 2e). Appropriate response to estrogen is essential for normal implantation^29,30^, and the critical role of trophoblast in immune modulation during pregnancy is well established^31^. Intriguingly, EnrichR analysis identified SOX2 as a potent transcription factor regulator of these genes, raising the possibility that SOX2 acts as negative effector for this group of genes (Fig. 2e). Together, these data suggest that the transcriptome of GOKM-hiTSCs is highly similar to that of hbdTSCs, and that GOKM reprogramming to hiTSCs overcomes inherent challenges present in OSKM-hiTSC reprogramming.

### GOKM and OSKM remodel the chromatin in distinct ways

**Fig. 3:**
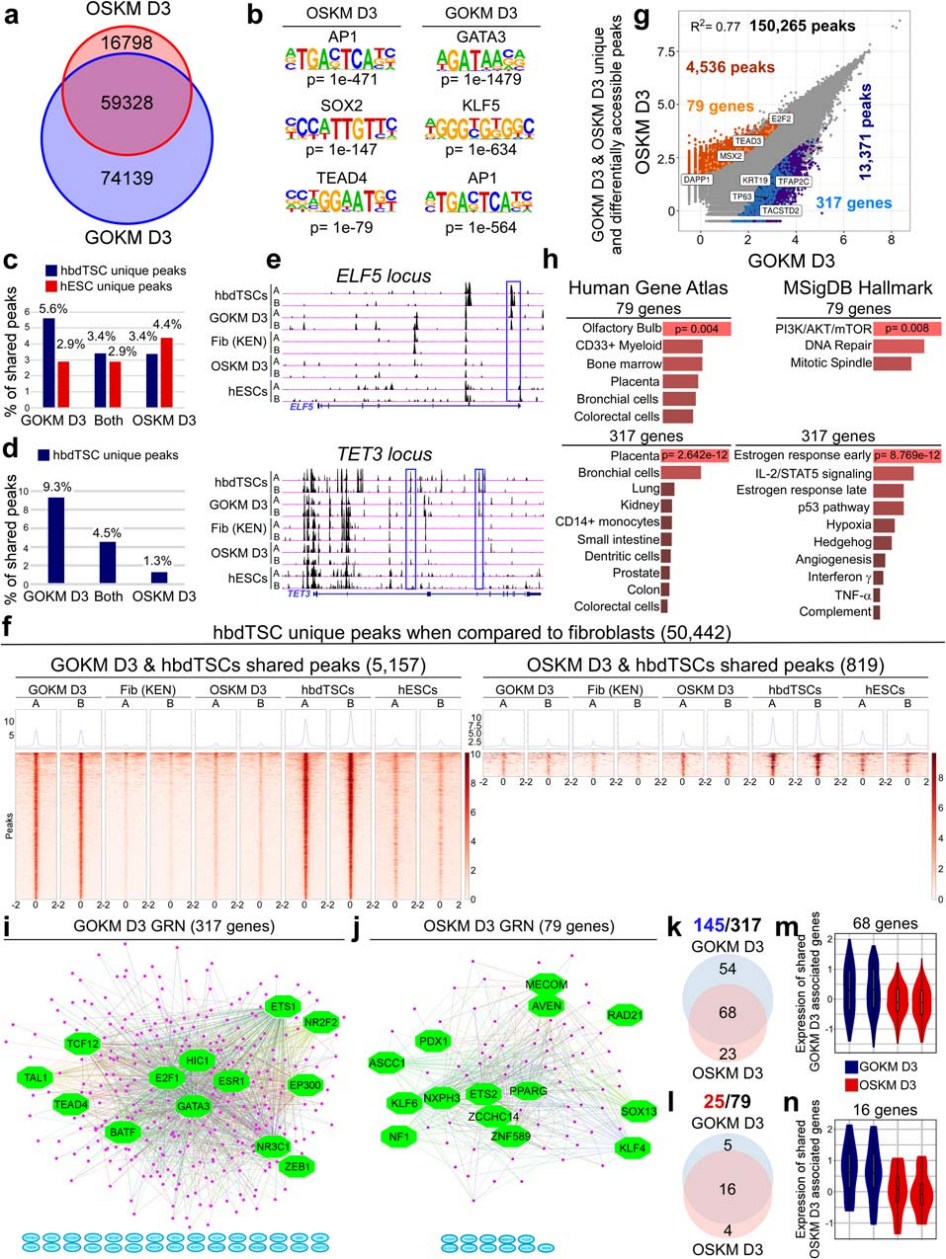
GOKM and OSKM exhibit different chromatin accessibility. **a** Venn diagram of day 3 GOKM and OSKM-unique peaks after subtracting “fibroblasts” peaks (FDR < 0.05). **b** HOMER motif analysis on “GOKM D3” and “OSKM D3”-differentially accessible peaks. *p* value was calculated using binomial distributions. **c** Graph summarizing the overlapping GOKM/OSKM-unique peaks with either hbdTSC/hESC-unique peaks as a percentage from the total GOKM/OSKM-unique peaks. **d** Graph summarizing the overlapping GOKM/OSKM-unique peaks with hbdTSC-unique peaks as a percentage from the total hbdTSC-unique peaks. **e** ATAC-seq signal at the *ELF5* and *TET3 loci*. Blue rectangles mark “GOKM D3” and “hbdTSCs” shared peaks. **f** Heatmap of 5157 GOKM and the 819 OSKM-unique peaks that overlap with the 50,442 hbdTSC-unique peaks (FDR < 0.05). **g** Scatter plot of differentially accessible peaks between “GOKM D3” and “OSKM D3” (FDR < 0.05). Peaks that are exclusive to GOKM/OSKM are labeled with dark blue and dark orange, respectively. Peaks associated with hbdTSC-expressed genes are labeled with light blue (GOKM) and light orange (OSKM). **h** Bar graphs showing the most enriched GO terms, and their *p* value, for the 317 or 79 genes from (**g**) using EnrichR. *p* value was calculated using Fisher exact test. **i**, **j** Gene regulatory networks of 317 genes (**i**), and 79 genes (**j**) from (**g**) constructed by iRegulon plugin tool in Cytoscape. Transcription factor (FDR < 0.05), Network Enrichment Score (NES) > 2. Green represents key regulators, pink marks regulated genes and turquoise depicts genes with no association. **k**, **i** Venn diagrams showing the number of genes that are expressed in GOKM, OSKM or in both at day 3 among the 317 (**k**) or 79 genes (**l**). **m**, **n** Violin plots showing the expression level of the 66-shared genes (**m**) from the 145 genes (**k**) and the 16-shared genes (**n**) from the 25 genes (**l**) in day 3 GOKM and OSKM. Two biological replicates (*n* = 2) are used for each condition. The center line denotes the median (50th percentile), and box limits contain the dataset’s 25th to 75th percentiles. Black whiskers mark the 5th and 95th percentiles. See also Supplementary Figs. 3–5. Source data are provided as a Source Data file.

Given that OSKM are capable of producing hiTSCs, we next sought to understand whether GOKM and OSKM remodel the somatic chromatin in a similar manner during reprogramming. To that end, we profiled chromatin accessibility of cells transduced with GOKM or OSKM for 3 days using Assay for Transposase-Accessible Chromatin using sequencing (ATAC-seq). In parallel, we profiled the transcriptome of the cells using RNA-seq. We chose to profile day 3 (D3) of reprogramming because, in contrast to later stages in reprogramming, cells respond relatively homogenously to transgene induction at this time point^26^, allowing more accurate bulk analysis. Parental fibroblasts, hbdTSCs and hESCs were used as controls for both experiments. Peak calling analysis for all samples using MACS2 (FDR < 0.05) yielded a total of 295,114 peaks for the ATAC-seq samples. Using this data, we first sought to understand whether the chromatin landscapes of GOKM- and OSKM- induced cells at D3 undergo distinct remodeling toward an hTSC or pluripotent state. To address this question, we initially defined all ATAC-seq peaks that are unique to fibroblasts (72,567 peaks), hbdTSCs (44,263 peaks) and hESCs (32,510 peaks, Supplementary Fig. 3a). We then took all the differentially accessible peaks from each group of cells and associated each peak to its closest neighboring gene. Next, we plotted the peaks and associated genes that define each cell type within scatterplots (Supplementary Fig. 3b–d, orange and light blue dots mark peaks that are associated with genes that are expressed in the corresponding cell type (i.e., hbdTSCs, hESCs or fibroblasts) according to RNA-seq data, while red and dark blue dots represent peaks in which no association with gene expression was identified). Low coefficient of determination (*R*^2^, 0.01–0.15) between the three samples, as well as peaks that are associated with known cell type-specific genes (e.g., *GATA3*, *TP63* and *ELF5* for hbdTSCs, *SOX2*, *SALL4* and *NANOG* for hESCs, *POSTN* and *THY1* for fibroblasts) validated these sets of cell type-specific differentially accessible peaks (Supplementary Fig. 3b–d). Next, we aimed to identify newly remodeled ATAC-seq peaks from “GOKM D3” and “OSKM D3”. To do so, we defined all the unique peaks of “GOKM D3” and “OSKM D3” samples by subtracting all the peaks that are shared with fibroblasts (FDR < 0.05). Out of these OSKM and GOKM unique peak sets, 74,139 newly remodeled peaks were exclusive for “GOKM D3” and 16,798 newly remodeled peaks were exclusive for “OSKM D3”, while 59,328 newly remodeled peaks were shared between the two combinations (Fig. 3a). HOMER analysis on “GOKM D3” and “OSKM D3” differentially accessible peaks identified *GATA3* and *KLF5* as the most enriched motifs within GOKM peaks and *SOX2* and *TEAD4* motifs for OSKM peaks, further validating our analysis (Fig. 3b). Like in the mouse^32^, the motif of the fibroblastic safeguard family of proteins *AP1* was highly enriched as well in both peak sets (Fig. 3b). To reveal how GOKM and OSKM facilitate induction of TSC and pluripotent states, we overlapped “GOKM D3” and “OSKM D3” unique ATAC-seq peaks with hbdTSC unique or hESC unique peaks (Supplementary Fig. 3e, f). This analysis revealed that 5.6% of the total GOKM unique peaks overlapped with hbdTSC unique peaks, a fraction representing 9.3% of the total hbdTSC unique peaks (Fig. 3c, d). In contrast, only 3.4% of the total OSKM unique peaks overlapped with hbdTSC unique peaks, representing 1.3% of the total unique peaks of hbdTSCs (Fig. 3c, d). When hESC unique peaks were used for comparison, 4.4% of the total OSKM unique peaks overlapped with hESC unique peaks and only 2.9% of the total GOKM unique peaks overlapped with hESC unique peaks (Fig. 3c), suggesting that while OSKM are biased toward remodeling pluripotency-associated chromatin, GOKM are biased toward the hTSC state. Two examples for GOKM unique peaks that overlap with hbdTSC peaks in hTSC-specific genes (i.e., *ELF5* and *TET3*) are depicted in Fig. 3e (Fig. 3e, marked by blue rectangles).

Given that many pivotal stemness genes are shared between hESCs and hbdTSCs (e.g., *LIN28A*, *FGF4*, *NR6A1*, *ZFP42*, *DPPA2*, *TET3*, *MYBL2*), we next hypothesized that OSKM might activate the hTSC state by remodeling the chromatin of genes that are shared between hbdTSCs and hESCs. To test this hypothesis, we took the entire set of unique peaks of hbdTSCs and subtracted only the peaks that overlapped with parental fibroblasts (FDR < 0.05), leaving hbdTSC unique peaks that a fraction of them is also shared with hESCs. This gave rise to 50,442 peaks which we subsequently overlapped with either GOKM or OSKM unique peaks. In support of our hypothesis, while the 5157 overlapping GOKM peaks were shared mostly with hbdTSC peaks, but not with “OSKM D3”, parental fibroblasts or hESCs, the 819 OSKM overlapping peaks were shared with both hbdTSCs and hESCs (Fig. 3f). A reciprocal experiment with hESCs identified 38,689 hESC unique peaks that overlapped with 3211 GOKM unique peaks and with 983 OSKM unique peaks. Interestingly, the GOKM unique peaks that overlapped with hESC peaks also overlapped with hbdTSC peaks, although to a lesser extent (Supplementary Fig. 3g). In contrast, OSKM unique peaks mostly overlapped with hESC peaks but not with or very mildly with hbdTSC peaks (Supplementary Fig. 3g). These results imply that while the peaks shared between “GOKM D3” and hbdTSCs or with hESCs reflect remodeling of the chromatin toward the hTSC state, those shared between “OSKM D3” and hESCs or with hbdTSCs are mostly specific to pluripotency or a result of an overlap between chromatin which is remodeled in both pluripotency and hTSC state.

We then focused on the relation between chromatin remodeling and gene expression. We created scatterplots between “GOKM D3” and “OSKM D3” differentially accessible peaks (Fig. 3g and Supplementary Fig. 3h) and marked the peaks that overlapped with hbdTSC differentially accessible peaks (dark blue for GOKM (13,371 peaks) and dark orange for OSKM (4536 peaks)). Then, we associated these peaks to their neighboring genes and marked exclusive GOKM or OSKM peaks that are associated with hbdTSC-expressed genes (light blue for GOKM and light orange for OSKM, Fig. 3g and Supplementary Fig. 3h). This analysis revealed two groups of peaks. The first group contains GOKM and OSKM-exclusive peaks that are associated with the same hbdTSC-expressed genes (128 genes, Supplementary Fig. 3h) and the second group contains peaks that are associated with hbdTSC-expressed genes, but each gene is exclusive to either GOKM or OSKM (317 genes for GOKM and 79 genes for OSKM, Fig. 3g). Interestingly, the peaks associated with the 128 shared hbdTSC-expressed genes showed distinct binding motifs between GOKM and OSKM peaks (i.e., *SOX2* and *TEAD4* for OSKM and *GATA3* and *KLF5* for GOKM, Supplementary Fig. 3i). These genes were enriched for GO terms such as “Placenta” and “BMP receptor binding” (Supplementary Fig. 3j). The 79 genes associated with OSKM exclusive peaks were enriched for the GO terms “Olfactory Bulbs” and “PI3K/AKT/mTOR” pathway, while the 317 hbdTSC genes that are associated with GOKM exclusive peaks were enriched for the GO terms “Placenta” and “Estrogen response” (Fig. 3h). These results suggest that already at D3 of reprogramming, the chromatin of a gene set responsible for estrogen response is specifically remodeled by GOKM but not by OSKM, proposing a possible explanation as to why a 94 gene expression signature that is responsible for estrogen response is absent in the final OSKM-hiTSCs (Fig. 2d, e), as discussed above.

iRegulon analysis for the 317 GOKM-associated genes and the 79 OSKM-associated genes identified two distinct GRNs, emphasizing the differences in the regulation of GOKM and OSKM unique gene sets (Fig. 3i, j). Gene expression analysis on GOKM-induced cells and OSKM-induced cells revealed that 145 genes (54 in GOKM, 23 in OSKM and 68 in both) out of the 317 GOKM-associated genes and 25 (5 in GOKM, 4 in OSKM and 16 in both) out of the 79 OSKM-associated genes are expressed already at day 3 of reprogramming, demonstrating a higher potency of GOKM in activating hTSC-specific genes (Fig. 3k, l). In accordance with this notion, genes that are expressed by both combinations at day 3 of reprogramming (i.e., 68 for GOKM-associated genes and 16 for OSKM-associated genes) showed a higher level of expression in GOKM (Fig. 3m, n).

To understand the overall chromatin changes that occur during 3 days of GOKM and OSKM induction, we defined all the regions that become accessible or closed in OSKM and GOKM (closed-to-open (CO peaks), open-to-closed (OC peaks)) by comparing “GOKM D3” and “OSKM D3” overall peaks to fibroblast peaks (Supplementary Fig. 4a). Interestingly, while GOKM and OSKM were able to close roughly the same number of regions (GOKM—10,765 peaks (OSKM OO peaks), OSKM—9623 peaks (GOKM OO peaks), both—69,007 peaks), suggesting that both combinations have a similar ability to shut off fibroblastic gene networks, GOKM were much more efficient in opening of the chromatin (GOKM—71,466 peaks, OSKM—14,790 peaks, both—25,896 peaks). We then associated each peak set to its neighboring genes and ran GO term and HOMER analysis for each gene and peak set (Supplementary Fig. 4b–k). This analysis revealed that while OSKM uniquely remodeled chromatin regions that are associated with “Pluripotent stem cells” and responsible for “Cell cycle” and “DNA replication”, GOKM uniquely remodel chromatin regions that are associated with “Placenta cells” and “Metabolism” (Supplementary Fig. 4c, e). Interestingly, *GATA3* and *SOX2* were the only motifs that were highly enriched in these peak sets, and which distinguish GOKM from OSKM, respectively. The other motifs that were enriched in these peak sets and shared between the two combinations are members of the *AP1*, *KLF*, *TEAD* and *OCT* families and *CTCF* (Supplementary Fig. 4b, d). Regions that become open by both combinations were enriched for the same binding motifs (i.e., *AP1*, *KLF*, *TEAD* and *OCT* families and *CTCF*), but include also the well-known safeguard of the genome, p53. GO term analysis on genes associated with this peak set revealed that both “Placental cells” and “Pluripotent stem cells” are enriched for these genes. Genes from this list are also enriched for GO terms associated with “Viral infection” (probably due to the primary infection of GOKM and OSKM) and “EGF1 pathway” (Supplementary Fig. 4f, g). As expected, regions that become closed by GOKM and OSKM were both enriched for the GO terms “Fibroblasts”, and “TGF-β regulation of ECM” and for motifs of factors known to act as safeguards of the fibroblastic identity such as members of the family of *AP1*, *BACH*, *MAF*, *TEAD* and *CTCF*^32^ as well as key pluripotency and TSC factors such as *NANOG*, *ELF5*, *CDX2* and *TFAP2C* (Supplementary Fig. 4h–k). Once again, *GATA3* for GOKM and *SOX2* for OSKM were of the only motifs that separate GOKM OC peaks from OSKM OC peaks (Supplementary Fig. 4h, j).

Altogether, these data imply that GOKM are, in general, more potent factors in remodeling the chromatin, but they also suggest that while GOKM are directed toward the hTSC fate, OSKM are capable of activating the hTSC state as a byproduct of the activation of pluripotency.

### GOKM and OSKM exhibit different H3K4me2 deposition dynamics

To complement the ATAC-seq data, we performed chromatin immunoprecipitation and sequencing (ChIP-seq) for the histone mark H3K4me2 on OSKM and GOKM-transduced cells following 3 days of factor induction. We selected the histone mark H3K4me2 because it has been shown to mark closed regions that are designated to become open later in the reprogramming process^26,33^. Peak calling analysis for all samples using MACS2 (FDR < 0.05) yielded a total of 270,316 peaks for the ChIP-seq samples. As was described for the ATAC-seq, we initially defined unique (Supplementary Fig. 5a) and differentially deposited peaks (Supplementary Fig. 5b–d) for each cell type and visualized the peaks and their associated genes using Venn diagrams and scatter plots (Supplementary Fig. 5a–d). We then defined all the unique peaks of “GOKM D3” and “OSKM D3” samples by subtracting the peaks that are shared with fibroblasts. Out of these OSKM and GOKM unique peak sets, 20,419 H3K4me2 peaks were exclusive to “GOKM D3” and 21,025 H3K4me2 peaks were exclusive to “OSKM D3”, while 49,691 H3K4me2 peaks were shared between the two factor combinations (Supplementary Fig. 5e). We overlapped “GOKM D3” and “OSKM D3” unique peaks with hbdTSC unique peaks or with hESC unique peaks (Supplementary Fig. 5f, g). In contrast to the ATAC-seq analysis, “GOKM D3” and “OSKM D3” exhibited only mild differences in the overlapping peaks with hbdTSC-unique or hESC-unique peaks (i.e., 1927 peaks for “GOKM D3” and 1245 peaks for “OSKM D3” when compared to hbdTSC-unique peaks, Supplementary Fig. 5f and 741 peaks for “GOKM D3” and 877 peaks for “OSKM D3” when compared to hESC-specific peaks, Supplementary Fig. 5g).

We focused on the relation between chromatin remodeling and gene expression. We created scatterplots between “GOKM D3” differentially deposited peaks and “OSKM D3” differentially deposited peaks, and marked the peaks that overlapped with hbdTSC differentially deposited peaks (Supplementary Fig. 5h, k). In contrast to the unique peak set, the differentially deposited peak set showed a significant difference between GOKM and OSKM yielding 7-fold more differentially deposited peaks for GOKM compared to OSKM (dark blue for GOKM (6375 peaks) and dark orange for OSKM (900 peaks)). Then, we associated these peaks to their neighboring genes and marked exclusive GOKM or OSKM peaks that are associated with hbdTSC-expressed genes (light blue for GOKM and light orange for OSKM, Supplementary Fig. 5h, k). This analysis revealed two groups of peaks. The first group contains GOKM and OSKM exclusive peaks that are associated with the same hbdTSC-expressed genes (18 genes, Supplementary Fig. 5k) and the second group contains peaks that are associated with hbdTSC-expressed genes but are exclusive to either GOKM or OSKM (241 genes for GOKM and 51 genes for OSKM, Supplementary Fig. 5h). HOMER analysis on these peak sets identified again the *AP1* motif as one of the most enriched binding sites for OSKM exclusive peaks along with *ETV2*, *RUNX* and *SOX2*, while *GATA3*, *OCT4*, *KLF5* and *AP1* were enriched in GOKM- exclusive peaks (Supplementary Fig. 5i). The 51 genes associated with OSKM exclusive peaks are enriched for the GO terms “Pancreatic progenitors” and “Hedgehog signaling”, while the 241 genes that were associated with GOKM exclusive peaks are enriched for the GO terms “Trophoblast stem cells”, and similarly to the ATAC-seq results, they were enriched for the GO term “Estrogen response” (Supplementary Fig. 5j). These results suggest that not only the chromatin is being physically remodeled by GOKM toward the hTSC state, but that histone marks that define active regions are being deposited in these regions as well.

### GOKM reprogramming does not induce genomic aberrations

We then focused on our reprogramming approach and examined the stability and the functionality of GOKM-hiTSCs.

We started by asking whether the reprogramming process toward hiTSCs is prone to genomic aberrations. To that end, we subjected two hbdTSC lines, hbdTSC#2 and hbdTSC#9, and four GOKM-hiTSC clones, hiTSC#4, hiTSC#11, hiTSC#2 and hiTSC#1, to sensitive karyotyping measurements using an Affymetrix CytoScan 750 K array. Thorough analysis revealed that 50% of all clones from both origins (i.e., hbdTSCs and hiTSCs) harbor an intact karyotype. The other 50% of the clones exhibited few aberrations in a small fraction of the cells (Supplementary Fig. 6a). These results indicate that hiTSC colonies with an intact karyotype can be isolated and grown in culture and that the reprogramming process in itself does not facilitate genomic instability. However, the results do suggest that like in the mouse^10^, hTSC cells have intrinsic tendency for genomic instability and that hTSC culture conditions should be optimized, as prolonged culture period might sensitize the cells for genomic aberrations, similarly to human ESCs/iPSCs^34^. It is important to note that recent findings have highlighted the abundance of genomic aberrations and mosaicism in endogenous trophoblastic tissue as well^35^.

### hiTSCs exhibit extensive DNA de/methylation rewiring

Given that the GOKM-hiTSC gene expression profile is highly similar to that of hbdTSCs, we next wished to understand whether the epigenetic landscape of GOKM-hiTSCs and hbdTSCs is correspondingly equivalent. DNA methylation is an epigenetic property that has been shown to be modified at late stages of OSKM reprogramming to iPSCs^36^. To examine whether the DNA methylation landscape of hiTSCs is equivalent to that of hbdTSCs, we subjected five GOKM-hiTSC clones (hiTSC#1, hiTSC#2, hiTSC#4, hiTSC#11 and hiTSC#16) to reduced representation bisulfite sequencing (RRBS) analysis, a method which increases depth of sequencing by focusing on genomic regions that are enriched for CpG content. Two hbdTSC lines, hbdTSC#2 and hbdTSC#9, two primary fibroblast lines (KEN and GM2) and hESCs were used as positive and negative controls, respectively.

**Fig. 4:**
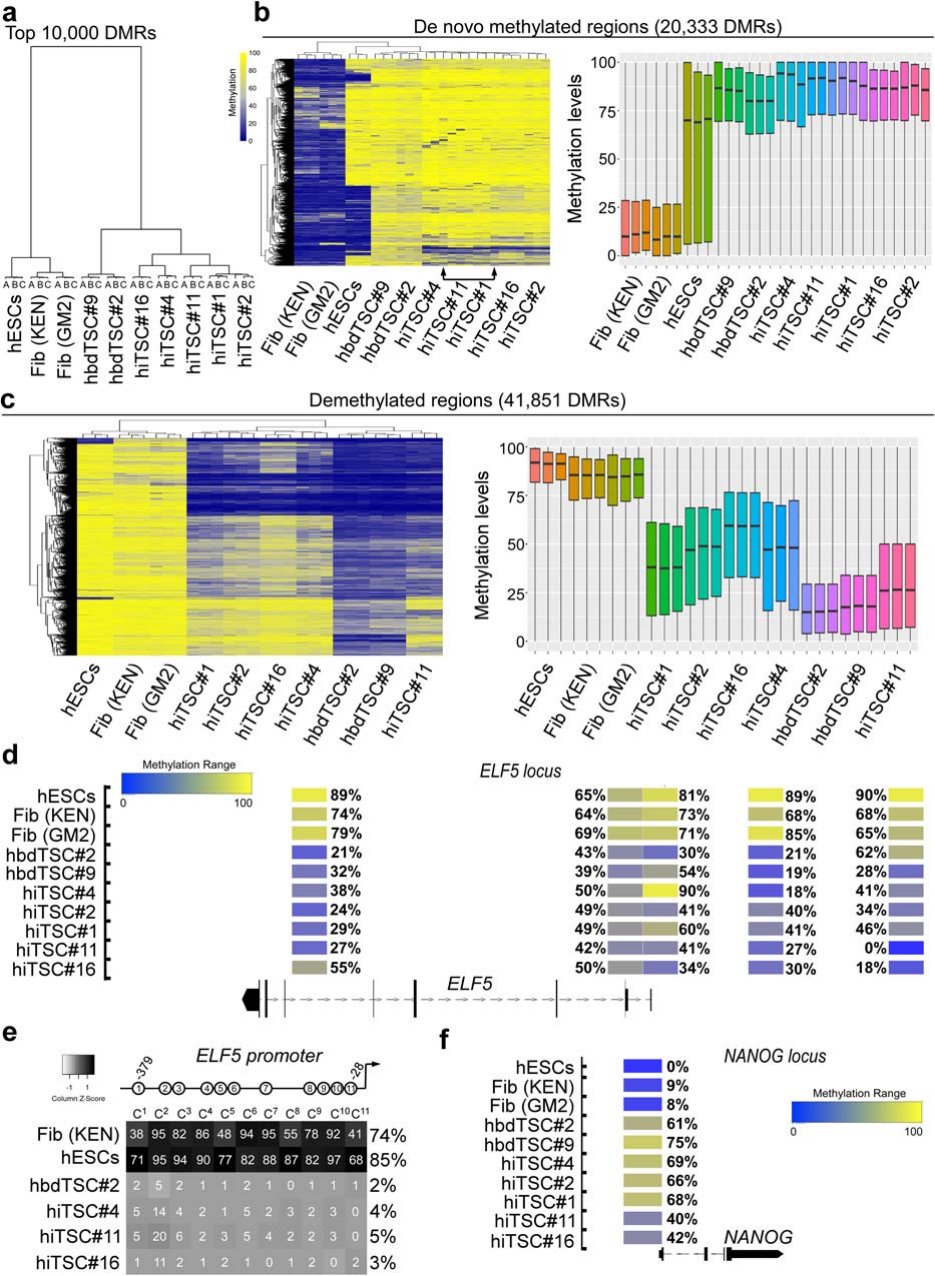
RRBS analysis demonstrates TSC-specific changes in methylation within hiTSCs. DNA methylation analysis of three biological replicates (*n* = 3) of fibroblasts (KEN and GM2), hESCs, two hbdTSCs lines, and five hiTSC clones as assessed by RRBS. Analysis of CpG methylation ratio with sequencing depth of at least 10 reads per CpG was computed, based on 100 bp tiles. **a** Dendogram for top 10,000 differentially methylated regions (DMRs) across all samples. **b** (Left) Heatmap showing 20,333 DMRs that are hypomethylated in fibroblasts and hypermethylated in hbdTSCs with a methylation difference above 50%. **b** (right) Boxplot of DNA methylation level across 20,333 DMRs (*n* = 20,333) from (**b**, left) in the indicated bulk samples. For each sample three biologically independent replicates (*n* = 3) were analyzed. Boxes indicate 50% (25–75%) and whiskers (5–95%) of all measurements, with black lines depicting the medians. **c** (left) Heatmap showing 41,851 DMRs that are hypermethylated in fibroblasts and hypomethylated in hbdTSCs with a methylation difference above 50%. **c** (right) Boxplot of DNA methylation level across 41,851 DMRs (*n* = 41,851) from (**c**, left) in the indicated bulk samples. For each sample three biologically independent replicates (*n* = 3) were analyzed. Boxes indicate 50% (25–75%) and whiskers (5–95%) of all measurements, with black lines depicting the medians. **d** Integrated genome browser capture of the methylation levels of five tiles that reside within the *ELF5 locus* in hESCs, fibroblast (KEN and GM2), hbdTSCs (hbdTSC#2 and hbdTSC#9), and five hiTSC clones, as assessed by RRBS. **e** Heatmap depicting the average methylation levels of 11 CG within the proximal *ELF5* promoter, in the indicated samples, as assessed by targeted bisulfite sequencing using MiSeq 2 × 150 bp paired end run. **f** Integrated genome browser capture of the methylation levels of one tile within the *NANOG locus*, in the indicated samples, as assessed by RRBS.

Hierarchical clustering analysis of the top 10,000 differentially methylated regions (DMRs) clustered all hiTSC clones and hbdTSC lines together and far from hESCs or parental fibroblasts, suggesting that the overall methylation landscape of hiTSCs is remodeled accurately and in close similarity to the methylation landscape of hbdTSCs (Fig. 4a). Deeper methylation analysis revealed 62,184 DMRs between fibroblasts and hbdTSC lines, 20,333 of which are hypomethylated in fibroblasts and hypermethylated in the hbdTSC lines, while the other 41,851 DMRs are hypermethylated in fibroblasts and hypomethylated in the hbdTSCs. Notably, analysis of the methylation landscape of the five hiTSC clones revealed robust de novo methylation in all five hiTSC clones, with a very small fraction of 1418 DMRs exhibiting partial de novo methylation (Fig. 4b), with their associated neighboring genes showing no significant association to hTSCs, hESCs or parental fibroblasts using GREAT and EnrichR. In contrast, the 41,851 comparatively hypomethylated DMRs showed less efficient demethylation activity. Approximately one-third of the tiles, which display significant association to placental loci, demonstrated complete hypomethylation in all hiTSC colonies, while the remainder of the tiles exhibited only partial demethylation with high levels of variation between different hiTSC colonies (Fig. 4c). Of note, one hiTSC clone, hiTSC#11, which was derived from the PCS201 fibroblast line, clustered closer to the two hbdTSC lines than to other hiTSC clones, which were derived from KEN (hiTSC#1, hiTSC#2, hiTSC#4) or GM2 (hiTSC#16) fibroblast lines (Fig. 4c).

These results suggest that demethylation is less rigorous in hiTSC reprogramming, but may also imply that the background, sex and the age of the parental fibroblasts may play a role in the efficiency of the demethylation process (i.e., GM2-derived hiTSC#16 clone that showed the lowest DNA demethylation capabilities was derived from fibroblasts isolated from adult female, compared to KEN and PSC, which are both HFF lines). Importantly, although the demethylation process is not optimal in hiTSCs, the overall methylation landscape of hiTSC clones clustered closely to hbdTSCs and far from hESC and fibroblast controls in both de novo methylated and demethylated DMRs (Fig. 4a–c).

Several TSC gatekeeper genes have been found to remain methylated during mouse ESC transdifferentiation toward TSC fate, producing cells which are TS-like but do not acquire complete TSC identity^12^. One of these gatekeeper genes is *ELF5*, the demethylation thereof is considered an important criterion for human trophoblast cell identity^23^. Thus, we examined whether the *ELF5* locus underwent demethylation in hiTSCs. Analysis of the RRBS data showed five DMRs in the *ELF5 locus*, demonstrating an overall equivalent pattern of hypomethylation between the two hbdTSC lines and all hiTSC clones (Fig. 4d). In agreement with the RRBS results, targeted direct amplification and next generation sequencing of the proximal *ELF5* promoter region after bisulfite conversion demonstrated vigorous demethylation in all 11 CpG promoter sites in both hbdTSCs and hiTSCs, but not in hESC and fibroblast controls (Fig. 4e).

We next examined the methylation levels of the pluripotency-specific locus *NANOG*, which is hypermethylated in mouse TSCs^10,13^. Similar to the mouse, the only DMR from the RRBS data that received coverage in this locus was completely hypomethylated in hESCs and to a lesser extent in fibroblasts, but equivalently methylated in both hbdTSCs and hiTSCs (Fig. 4f).

Taken together, these data suggest that DNA methylation is largely rewired to the hTSC state in the stable hiTSCs, but also suggest that improved reprogramming conditions need to be developed to induce a more robust demethylation.

### hiTSCs differentiate into all major trophoblast cell types

hTSCs have the ability to differentiate into multinucleated syncytiotrophoblast (STs) and extravillous trophoblasts (EVTs)^1^. Thus, our next goal was to examine whether GOKM-hiTSCs have the potential to differentiate into these various trophoblast subtypes.

**Fig. 5:**
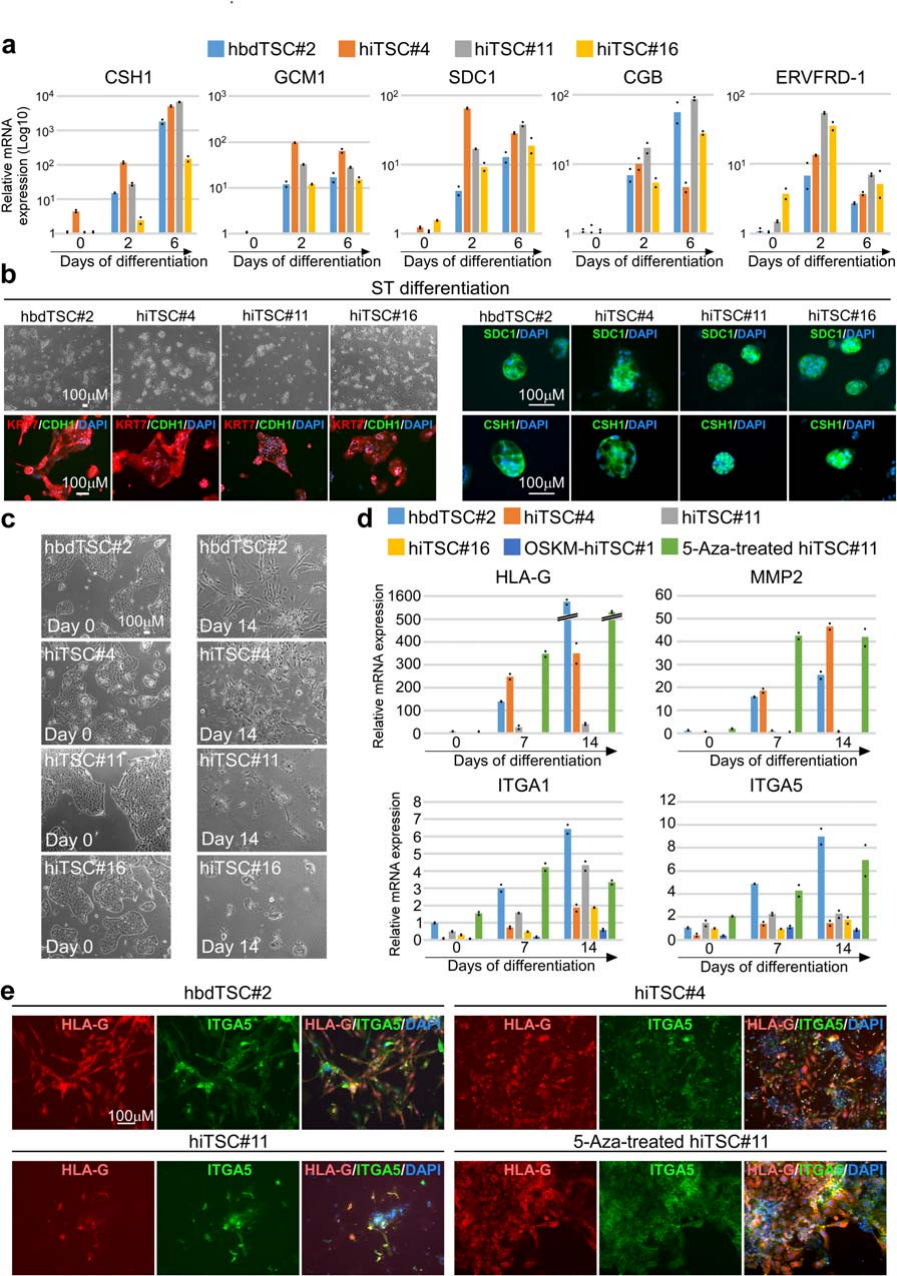
hiTSCs differentiate into multinucleated ST and EVT cells. hbdTSC#2, hiTSC#4, hiTSC#11 and hiTSC#16 were induced to differentiate into STs using previously established protocol^1^. **a** qPCR analysis of relative mRNA levels of ST-specific markers *CSH1*, *GCM1*, *SDC1*, *CGB* and *ERVFRD-1*, for the indicated samples, at days 0, 2 and 6 in medium for directed differentiation into STs (STM). Results were normalized to the mRNA levels of the housekeeping control gene *GAPDH* and are shown as Log base 10-fold change relative to day 0 control cells (hbdTSCs). For each sample two replicates were used (*n* = 2). hiTSC colonies were derived from three independent reprogramming experiments (*n* = 3). **b** Bright field images (left top) and fluorescent images for the pan-trophoblast marker KRT7 and the epithelial marker CDH1 (left bottom), or for the ST-specific markers SDC1 and CSH1 (right panel) following 6 days of directed differentiation to STs. DAPI staining was included to mark nuclei. Two and three dimensional multinucleated-positive cells are shown for three independent hiTSC clones (*n* = 3). **c** Bright field images of the indicated hbdTSC and three independent hiTSC clones (*n* = 3) at day 0 and following 14 days of EVT differentiation. **d** qPCR analysis of relative mRNA levels of EVT-specific markers *HLA-G*, *MMP2*, *ITGA5* and *ITGA1* at days 0, 7 and 14 of directed differentiation into EVTs. Results were normalized to the mRNA levels of the housekeeping control gene *GAPDH* and are shown as fold change relative to day 0 control cells (hbdTSCs). For each sample two replicates (*n* = 2) were used. Three independent hiTSC clones (*n* = 3) were assayed. **e** Immunofluorescence staining for the EVT-specific markers HLA-G and ITGA5 and DAPI in PFA-fixated hbdTSC#2, hiTSC#4, hiTSC#11, and 5-Aza-treated hiTSC#11 (*n* = 3) following 14 days of EVT differentiation. See also Supplementary Fig. 6. Source data are provided as a Source Data file.

We performed directed differentiation into STs and EVTs using previously published protocols^1^. Initially, we differentiated hbdTSCs and three GOKM-hiTSC clones (hiTSC#4, hiTSC#11 and hiTSC#16) into STs and collected samples at days 2 and 6 of differentiation. qPCR analysis for ST markers such as *CSH1*, *GCM1, SDC1, CGB, PSG1, CHSY1 and ERVFRD-1*^1,23^ showed robust induction of ST markers in hiTSCs, equivalent to hbdTSCs with some variations between the clones (Fig. 5a and Supplementary Fig. 6b). Of note, ERVFRD-1, which is an endogenous retroviral gene expressed by cytotrophoblastic ST-precursor cells and orchestrates the fusion event early in the syncytialization process^37^, showed a rapid and transient upregulation on day 2 of differentiation (Fig. 5a). Bright field images and immunostaining for the pan trophoblast KRT7, the epithelial marker CDH1 and DAPI showed clear formation of large KRT7-positive multinucleated cells after 6 days of differentiation in both hbdTSCs and hiTSCs (Fig. 5b). As expected, while the undifferentiated hiTSCs stained positive for CDH1 (Fig. 1e), staining in multinucleated STs was significantly reduced. CSH1 and SDC1-positive three-dimensional ST structures were observed in all hiTSC clones and in the hbdTSC#2 positive control (Fig. 5b). These data indicate that hiTSCs are capable of differentiating into STs similarly to hbdTSCs.

We next performed directed differentiation of hbdTSCs and GOKM-hiTSCs into EVTs^1^. Following seeding and cell attachment to the plate, cell aggregates formed in all tested clones. However, after 14 days of differentiation, the efficiency in the production of EVTs varied widely between clones. While hbdTSC#2 and hiTSC#4 showed significant differentiation into EVTs as assessed by morphology, EVT gene expression (i.e. *HLA-G*, *MMP2*, *ITGA5* and *ITGA1*^1,23^,) and staining for the EVT markers HLA-G and ITGA5 (Fig. 5c–e), hiTSC#11 and hiTSC#16 as well as OSKM-hiTSC#1 demonstrated only a partial capability in producing EVT cells (Fig. 5d, e and Supplementary Fig. 6c). In general, we observed two main morphologies of HLA-G-positive cells following 14 days of EVT differentiation; (1) spindle-shaped and (2) small and migratory cells. Interestingly, while the spindle-shaped cells exhibited strong HLA-G staining and weak ITGA5 expression, the small and migratory cells stained strongly for both HLA-G and ITGA5 (Fig. 5e and Supplementary Fig. 6c). The appearance of multiple morphologies of EVTs in our experiments is in accordance with previous studies showing significant diversity between various EVT subtypes^38^.

We sought to examine whether the partial DNA demethylation observed in some hiTSC clones or reactivation of viral vectors during differentiation might contribute to the inconsistency seen during EVT differentiation. To that end, we treated hiTSC#11 and hiTSC#16 with 5-aza-2′-deoxycytidine (5-Aza), a known demethylation agent, for 2 days and performed EVT differentiation. In parallel, we utilized a previously described non-integrating episomal reprogramming technique^39^, replacing the hSOX2 with hGATA3, and reprogrammed the cells into hiTSCs.

Interestingly, one of two 5-Aza treated GOKM-hiTSC clones (i.e., hiTSC#11) restored its capability to differentiate into EVTs (Fig. 5d, e) and 1 out of 3 examined episomal-derived hiTSC clones (hiTSC^episomal^#7) showed robust differentiation into EVTs (Supplementary Fig. 6d).

These results suggest that suboptimal demethylation can hinder the differentiation potential of the cells and that transgene integration most probably do not contribute to the inconsistency seen in EVT differentiation between hiTSC colonies. It is important to note that hiPSC clones also harbor various propensities for differentiation^40^ and that this variability is an intrinsic property within reprogramming systems.

Taken together, these results suggest that some hiTSC clones harbor differentiation potential similar to hbdTSCs, but also imply that hiTSC formation and differentiation protocols need to be optimized to unleash the full differentiation potential of the cells.

### hiTSCs form trophoblastic lesions

**Fig. 6:**
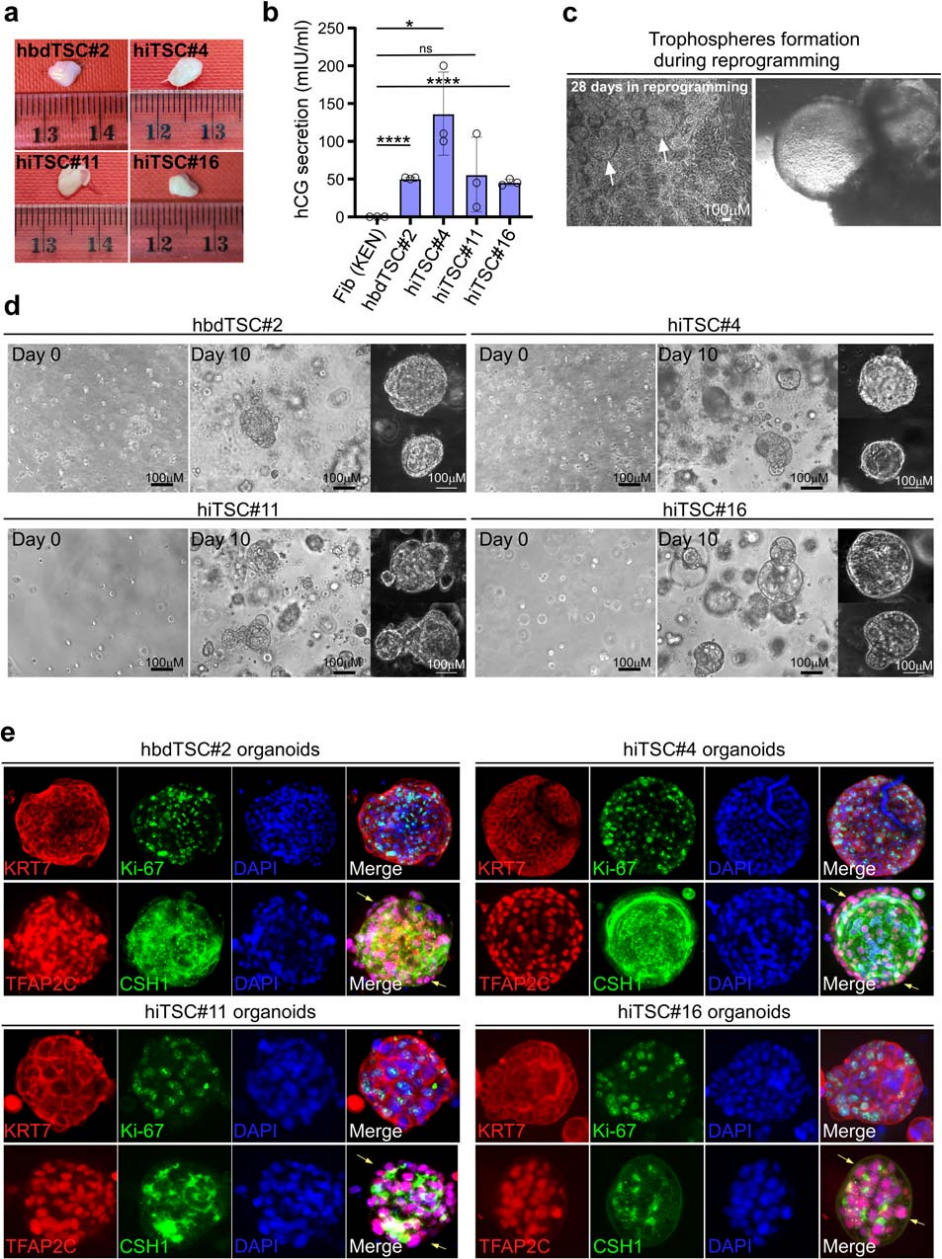
hiTSCs form trophoblastic lesions in NOD/SCID mice and functional organoids in matrigel. **a** Representative images of the lesions that were extracted from NOD/SCID mice following ~9 days of subcutaneous injection of ~4 × 10^6^ cells from hbdTSC#2, hiTSC#4, hiTSC#11 and hiTSC#16 lines. Each line was injected into three NOD/SCID mice (*n* = 3). **b** Graph showing the concentration of hCG secretion in the serum of injected NOD/SCID male mice with the indicated cells. Approximately 500 μl of serum was collected from each mouse and used for hCG detection, using hCG ELISA kit (Alpco). Error bars indicate standard deviation between 3–4 independent experiments/replicates (*n* = 3 for hiTSCs and Fib (KEN), and *n* = 4 for hbdTSC#2). **** indicates *p* value < 0.0001 (95% confidence interval 47.59–53.41 for Fib (KEN) vs. hbdTSC#2 and 39.19–52.15 for Fib (KEN) vs. hiTSC#16), * indicates *p* value of 0.0127 (95% confidence interval 48.38–225.0), ns indicates not statistically significant, using two-tailed unpaired *t-*test calculated by GraphPad Prism (8.3.0). Mean values (from left to right) are: 0.000, 50.50, 136.7, 56.00, 45.67. **c** Bright field images of trophospheres within a reprogramming plate at day 28 of dox treatment following GOKM infection. **d** Bright field images of hbdTSC#2 and 3 independent hiTSC clones (hiTSC#4, hiTSC#11 and hiTSC#16, *n* = 3) at day 0 and 10 of organoid formation protocol. Note that by day 10 of the protocol, large and 3-dimantional organoid structures were generated in each clone. **e** Spinning disk confocal images of organoids from (**d**), following immunofluorescence staining for pan-trophoblast marker KRT7, proliferative cell marker Ki-67, TFAP2C and CSH1, with DAPI nuclear staining. Yellow arrows indicate areas of undifferentiated cells. See also Supplementary Fig. 7. Source data are provided as a Source Data file.

When mouse TSCs/iTSCs are injected subcutaneously into nude mice, the cells differentiate into the various trophoblast subtypes and orchestrate robust invasion and endothelial cell recruitment to form transient hemorrhagic lesions. Conversely, when hbdTSCs are injected subcutaneously into non-obese diabetic (NOD)-severe combined immunodeficiency (SCID) mice, the cells form KRT7-positive trophoblastic lesions with little differentiation and blood vessel formation^1^. To test whether GOKM-hiTSCs are capable of forming similar trophoblastic lesions, we subcutaneously injected ~4 × 10^6^ cells from three GOKM-hiTSC clones (hiTSC#4, hiTSC#11 and hiTSC#16) and one hbdTSC control line hbdTSC#2, into NOD/SCID male mice. Nine days later, when the lesions reached ~5 mm in size (Fig. 6a), the lesions and blood serum were extracted and examined. Using enzyme-linked immunosorbent assay (ELISA), we quantified the level of the human pregnancy hormone hCG in the serum of the injected male mice. When fibroblasts were injected, the serum levels of hCG were undetectable, while serum levels following injection of hiTSC and hbdTSC clones were in the range of 40–130 mlU/ml (Fig. 6b). Immunohistochemical staining showed that all lesions were KRT7-positive and that small regions of cells had differentiated into EVTs and STs (Supplementary Fig. 7a), similarly to previously published findings^1^. These results imply that hiTSCs can generate trophoblastic lesions in NOD/SCID mice that are similar in their characteristics to lesions that are formed by hbdTSCs.

### hiTSCs form functional trophoblast organoids

Recently, two trophoblast organoid systems have been developed and described^41,42^. These studies demonstrated the capability of first trimester villous CTB cells to form three-dimensional structures which contain both proliferating stem cells and differentiated cells. In-depth examination of the two systems revealed that many characteristics of the early developmental program of the human placenta are present in these organoid platforms^41,42^.

Thus, we next asked whether GOKM-hiTSCs harbor similar potential to form trophoblastic spheres and functional organoids. Initially we observed, albeit occasionally, regions within the reprogramming plates which generated trophoblastic spheres (Fig. 6c), suggesting that these cells are capable of forming three-dimensional structures. Notably, these trophospheres were recently suggested to mark naïve hTSC (i.e., similar to pre-implantation TE^7^). To examine whether GOKM-hiTSCs are capable of forming organoids as well, we employed a previously published protocol for trophoblast organoid formation^41^. hiTSC#4, hiTSC#11, hiTSC#16 and control hbdTSC#2 were trypsinized and seeded as single cells inside a droplet of matrigel and allowed to grow for 10 days. As shown for villous CTBs^41,42^, both hbdTSCs and hiTSC clones were capable of forming three-dimensional structures within few days of culture (Fig. 6d). Immunostaining for the pan-trophoblast marker KRT7 and the proliferation marker Ki-67 followed by confocal microscopy examination revealed KRT7-positive organoids with proliferating cells, suggesting that undifferentiated cells were still present within the organoids after 10 days of culture (Fig. 6e). Immunostaining for TFAP2C and CSH1 validated an outer layer of cytotrophoblasts with a core of STs, displaying the inverted placental villous structure as previously described^41,42^. Taken together, these data demonstrate that hiTSCs can form functional organoids that are similar to their hbdTSC and villous CTB counterparts.

### hiTSC reprogramming with GOKM bypasses pluripotency

Given that OSKM can generate both hiPSCs and hiTSCs^3,9,43^ and since we show here that GATA3 can replace SOX2 in generating hiTSCs, we next asked whether OCT4, KLF4 and MYC (OKM) are sufficient to generate hiTSCs. Supplementary Figs. 1d and 8a–e demonstrate that the OKM combination is, in principle, incapable of producing neither hiTSCs nor hiPSCs as assessed by colony number and expression of hTSC and pluripotency gene markers in reprogramming plates (Supplementary Figs. 1d and 8a–e). Of note, very rarely and following multiple attempts, we surprisingly managed to isolate two hiPSC colonies, but not hiTSC colonies, with the OKM factors only (Supplementary Fig. 7b).

Additionally, since multiple components of the hTSC medium have been shown to enhance mouse and human reprogramming to iPSC^44,45^, we tested the capability of GOKM to generate hiPSCs. The lack of hiPSC marker expression in hiTSC reprogramming plates hints that the hTSC medium does not support pluripotent cells (Supplementary Fig. 8e). Nevertheless, we transduced fibroblasts with the GOKM factors and conducted reprogramming with an established hiPSC reprogramming protocol. No hiPSC colonies emerged. We next repeated this experiment, but instead of using the hiPSC reprogramming protocol, we began with the hTSC reprogramming protocol and upon dox withdrawal we changed the medium to hESC-supportive medium. Following three independent reprogramming experiments, we observed the formation of only two hiPSC colonies that were positive for hESC markers and integrated all four GOKM transgenes (Supplementary Figs. 7b and 8f). According to the transgene integration analysis, it is evident that the GOKM-hiPSC colonies integrated higher levels of *MYC* and lower levels of *KLF4* in comparison with the GOKM-hiTSC colonies (Supplementary Fig. 8f). The importance of factor stoichiometry and levels in determining cell identity was recently shown by our group when one combination of five transcription factors was able to induce three different cell types of the mouse pre-implantation embryo depending on various transgene levels^11^.

In order to further scrutinize the formation of hiPSCs during GOKM reprogramming, we sorted for pluripotency cell-surface marker TRA-1-60 positive cells at various time points during GOKM reprogramming, then seeded them and counted the hiPSC colonies which emerged (Supplementary Fig. 8g). We chose to sort even very weakly positive cells as so not to omit any potential hiPSCs at the cost of a higher false-positive sorting rate. Although hiPSC colonies emerged after seeding sorted cells from OSKM reprogramming and hiPSC positive controls, no hiPSC colonies emerged in plates seeded with cells sorted from GOKM reprogramming in various time points in the hiTSC reprogramming protocol, following GOKM reprogramming with hiPSC protocol or after switching to hESC-supportive medium after dox withdrawal following hiTSC protocol (Supplementary Fig. 8g).

**Fig. 7:**
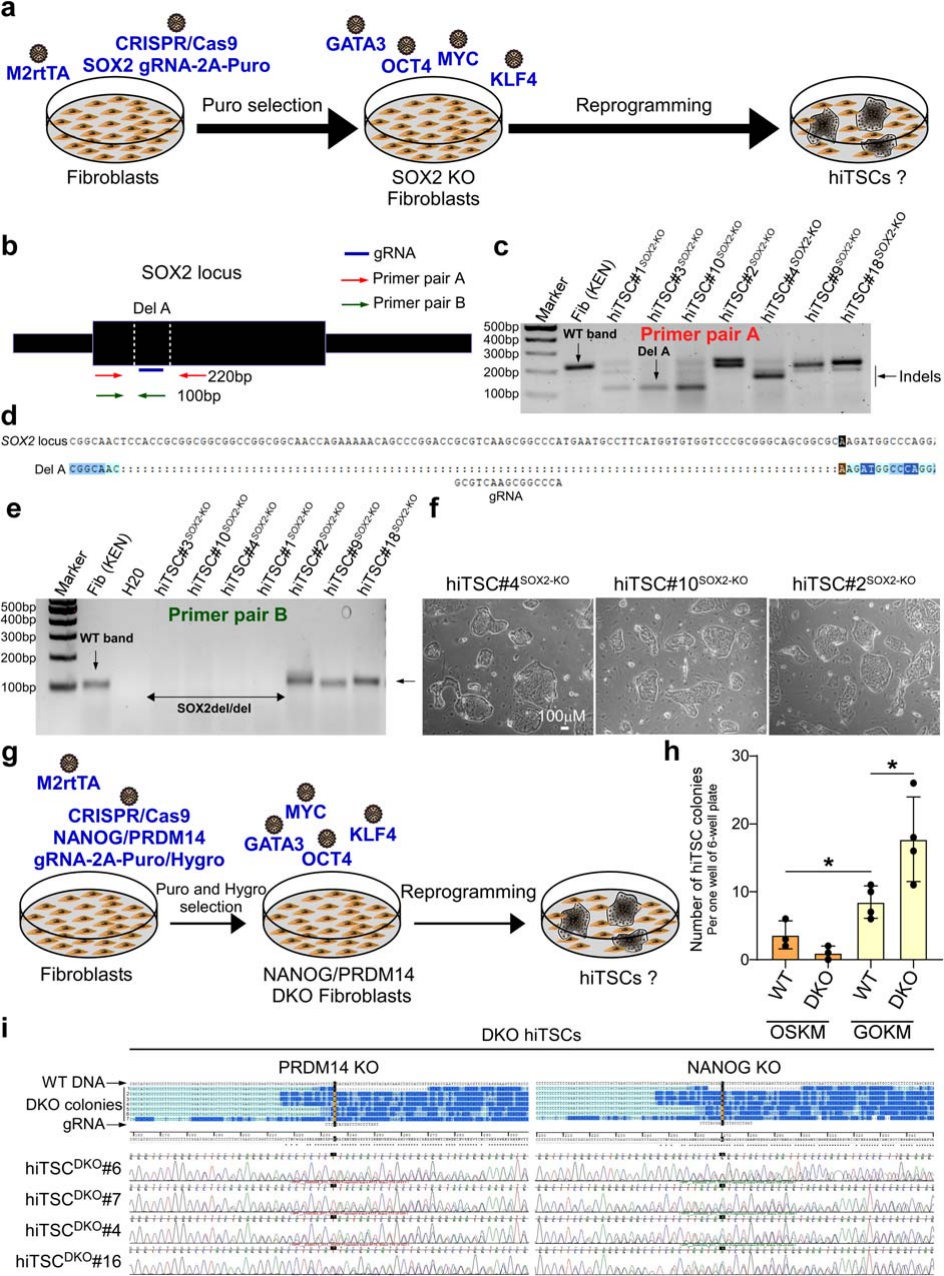
GOKM produce hiTSCs independently to SOX2 or PRDM14 and NANOG expression. **a** Schematic representation for the strategy to generate and reprogram knockout (KO) *SOX2* fibroblasts into hiTSCs. **b** Schematic representation of the *SOX2* gene locus, displaying the location of the gRNA used, as well as the primer pairs designed to examine indels**. c** DNA gel showing a WT band of 219 bp within the *SOX2* coding region in WT fibroblasts and the same PCR reaction in seven independent hiTSC clones (*n* = 7) from *SOX2* KO fibroblasts. **d** Sequence alignment image of one indel event (i.e., Del A) within the *SOX2 locus* in hiTSC#3^SOX2-KO^ using Sequencher software (demo version). **e** DNA gel showing a WT band of 100 bp within the *SOX2* coding region in WT fibroblasts and the same PCR reaction in seven independent hiTSC clones (*n* = 7) from *SOX2* KO fibroblasts. Note, 4 out of 7 colonies show a bi-allelic deletion within *SOX2* coding region (SOX2del/del). **f** Bright field images of three *SOX2 KO* hiTSC colonies (*n* = 3). **g** Schematic representation for the strategy to double knockout (DKO) *NANOG* and *PRDM14* in fibroblasts and reprogramming into hiTSCs. **h** Graph depicting the number of hiTSC colonies that emerged either in DKO fibroblasts or in WT controls following reprogramming with GOKM or OSKM. Error bars indicate standard deviation between 3–4 experiments/replicates (for OSKM *n* = 3 and for GOKM *n* = 4). * indicates *p* value of 0.0383 for OSKM WT vs. GOKM WT and 0.0324 for OSKM WT vs. GOKM WT (95% confidence interval of 0.3851–9.282 and 1.081–17.42, respectively), using two-tailed unpaired *t-*test calculated by GraphPad Prism (8.3.0). Mean values (from left to right) are: 3.667, 1.000, 8.500, 17.75. **i** Sequences alignment image of various indel events within the *NANOG* and *PRDM14 loci* in seven independent hiTSC^DKO^ clones (*n* = 7) using Sequencher (Demo version). gRNA sequences and Sanger chromatograms are shown for 4 hiTSC^DKO^ clones. Note that a significant enrichment for double KO events is evident in hiTSC clones that were derived from DKO fibroblasts. See also Supplementary Figs. 7–10. Source data are provided as a Source Data file.

Although the emergence of hiPSC colonies with GOKM is evidently extremely rare, it prompts further investigation of whether GOKM-derived hiTSCs undergo a stage of transient pluripotency. To address whether pluripotency is a requirement for hiTSC formation, we utilized a previously published lentiviral-vector constitutively expressing the CRISPR/Cas9 protein and gRNA, which was shown to produce a very high occurrence of indels^46^. Using bulk infection, we generated a heterogeneous population of *SOX2* knockout (KO) fibroblasts that also express the Tet-On system transactivator, M2rtTA (Fig. 7a). Since pluripotency cannot be maintained without *SOX2*^47^, obtaining hiTSC colonies that are *SOX2* KO with GOKM indicates that pluripotency is not required for achieving the hTSC state during reprogramming. *SOX2* KO fibroblasts were reprogrammed into hiTSCs with GOKM and several hiTSC colonies were isolated and propagated. Careful examination of the resulting colonies revealed that 7 out of 7 examined colonies contained *SOX2* indels (Fig. 7b–d) and 4 out of 7 contained a bi-allelic deletion within the *SOX2* coding region (Fig. 7e). *SOX2* KO hiTSC colonies exhibited normal morphology (Fig. 7f) and comparable gene expression to WT hiTSCs and hbdTSCs (Supplementary Fig. 8h). To confirm functional KO of *SOX2* in the cells, we reprogrammed *SOX2* KO fibroblasts into hiPSCs with human OKM and mouse *Sox2* vector. This approach allowed us to distinguish between the endogenous human *SOX2* from the exogenous mouse *Sox2* gene in the resulting colonies. We were able to generate hiPSC colonies with OKM and mouse *Sox2*, although following dox withdrawal many hiPSCs collapsed and the ones that retained pluripotency were KO-escapees, which harbored at least one functional allele of *SOX2* as assessed by qPCR analysis (Supplementary Fig. 8i–l). This was in stark contrast to hiTSCs that demonstrated full homozygous deletion in 4 out of 6 examined colonies (Supplementary Fig. 8i). To validate our results, we generated an additional KO fibroblast line in which both *NANOG* and *PRDM14* were targeted with specific gRNAs (Fig. 7g). In agreement with the *SOX2* KO results, GOKM efficiently produced hiTSCs from these DKO fibroblasts (Fig. 7h). In contrast, DKO fibroblasts showed significantly reduced capacity to reprogram into hiPSCs (Supplementary Fig. 8m). Sequencing of the gRNAs regions of seven DKO hiTSC colonies followed by chromatogram analysis revealed that 6/7 colonies contained homozygous indels causing a frameshift of the gene for both *PRDM14* and *NANOG*, while 1/7 colonies contained homozygous indels for *PRDM14* and a heterozygous indel for *NANOG* (Fig. 7i). These data indicate that SOX2, NANOG and PRDM14 are dispensable for the formation of hiTSCs.

### GOKM and OSKM activate distinct gene signatures

To exclude the possibility that GOKM induce the hTSC state by activating pluripotent gene signature at some point during the reprogramming process, we performed GOKM and OSKM hiTSC reprogramming and collected cells at 3D, 6D, 12D, 18D and 24D after factor induction. To segregate between the effect of GATA3 and SOX2 in activating the hTSC state, we included an additional hiTSC reprogramming experiment with GATA3, SOX2, KLF4 and MYC (GSKM), a combination known to induce pluripotency^48^. Given that OCT4 is essential for hiTSC induction, no hiTSCs are expected to be formed by GSKM (Supplementary Fig. 1a), allowing a clear segregation between the role of GATA3 as pluripotency inducer and its role as hTSC state inducer. RNAs from the different reprogramming combinations (i.e., OSKM, GOKM and GSKM) were extracted and subjected to RNA-seq at the various time points during reprogramming.

Initially we sought to understand whether cells induced by OSKM or GOKM express a shared gene signature at some point during the reprogramming process, an observation that may suggest that GOKM are capable of activating a complete or partial pluripotent state. To address this question, we used principal component analysis (PCA) and plotted all the time points of OSKM and GOKM reprogramming on the same graph, while in each graph different pairs of principal components were used (i.e., PC1 vs. PC2, PC1 vs. PC3 and PC2 vs. PC3, Supplementary Fig. 9a–c). In accordance with our hypothesis, no gene signature overlap was seen between the two reprogramming combinations in all the tested combinations of principal components (Supplementary Fig. 9a–c). We then produced a correlation heatmap for all samples and time points (Supplementary Fig. 9d). Again, OSKM mostly clustered separately from GOKM and GSKM, suggesting that OCT4 in conjunction with SOX2 activates a unique gene signature which is different from the gene signatures that are activated when OCT4 is combined with GATA3 or when SOX2 is combined with GATA3. However, when the top 1000 most variable genes where analyzed by PCA, GSKM reprogramming samples partially overlapped with OSKM samples, supporting the notion that GSKM are capable in activating, at least partially, a similar set of genes as OSKM (Supplementary Fig. 9e). We then defined a rigorous (logFC>6) pluripotency gene signature by selecting genes that are uniquely expressed in pluripotent cells when compared to hbdTSCs and fibroblasts. This yielded 289 genes that are highly unique to pluripotent cells (Supplementary Fig. 9f). Hierarchical clustering focusing on this set of genes revealed 3 main clusters. While OSKM-induced cells mostly activated genes from cluster 3 and at late stages of reprogramming also a fraction of genes from cluster 2, GSKM, that were shown to induce pluripotency as well, mostly activated genes from cluster 1 (Supplementary Fig. 9f). In contrast, GOKM showed scant and scattered expression from the different clusters. In accordance with that, violin plots for all reprogramming combinations and time points, using clusters 1 and 3, demonstrated a significant upregulation trendline in OSKM (*p* < 1e−05), and to a lesser extent in GSKM, compared to GOKM (Supplementary Fig. 9g). These data suggest that GOKM activate pluripotency unique genes to a minimal extent.

We then defined a rigorous (logFC > 6) hTSC-specific gene signature. This yielded 201 genes that are highly unique to hbdTSCs (Supplementary Fig. 9h). Hierarchical clustering focusing on this set of genes revealed 3 main clusters. Interestingly, all combinations were capable of activating a set of hTSC genes. While OSKM-induced cells mostly activated genes from cluster 2 (OCT4-specific activation) and GSKM activated genes from cluster 1 (GATA3-specific activation), GOKM activated genes from both clusters, mainly at late stages of reprogramming (Supplementary Fig. 9h). These data demonstrate the additive effect of combining GATA3 with OCT4 in activating the hTSC state.

Finally, to identify and characterize any differences in the mechanisms by which GOKM and OSKM induce the hTSC state, we took all the genes that showed differential expression (LogFC > 3) between OSKM and GOKM in at least one-time point during the reprogramming process and performed clustering analysis (Supplementary Fig. 10). Gap statistics analysis on the identified 706 differentially expressed genes resulted in 10 defined clusters. Clusters 1 and 2 are specific to fibroblasts, while cluster 5 is specific to pluripotent cells, and cluster 6 to hbdTSC cells. Clusters 4 and 7 are shared between pluripotent and hbdTSC cells, while cluster 3 is mostly specific for OSKM reprogramming. Cluster 10 is mostly specific to GOKM reprogramming, while cluster 9 is shared between GOKM and GSKM reprogramming. Cluster 8 is partially present in pluripotent cells and is shared between GOKM and the final steps of OSKM reprogramming.

Importantly, these clusters emphasize the different functions GATA3, OCT4 and SOX2 exert in the activation of pluripotency and hTSC state in conjunction with other transcription factors. For example, genes from cluster 5 that are specific to pluripotent cells are specifically upregulated by OCT4 but become much further upregulated when SOX2 is present in the combination. Genes from cluster 6 that are unique to hTSCs are specifically upregulated by GATA3 but become further upregulated at late stages of reprogramming in the absence of SOX2, suggesting an inhibitory effect of SOX2 on hTSC gene signature. Genes from cluster 7 which are shared between pluripotent cells and hTSCs are specifically upregulated when GATA3 is combined with OCT4 but not when GATA3 is combined with SOX2 or when OCT4 is combined with SOX2.

Taken together, this analysis uncovers how different transcription factors within different reprogramming combinations regulate unique sets of genes that are important for the induction of pluripotency and the hTSC state. It emphasizes again that while OSKM is mostly biased toward pluripotency by activating pluripotency-specific set of genes (cluster 5) and a cluster of pluripotency and hTSC shared genes (cluster 4), GOKM is mostly biased toward the hTSC state by activating hTSC-specific gene sets (cluster 6) as well as genes shared by pluripotency and the hTSC state (cluster 7).

## Discussion

Placental disorders such as preeclampsia and intra-uterine growth restriction are commonly detected at late stages of pregnancy, when proliferative villous CTBs are no longer available for isolation and exploration. Thus, developing a method to reprogram differentiated cells derived from disease-affected placenta or cord blood into functional TSCs is of vital importance for modeling and identifying potential risk factors for placental disorders, as well as for possible future cell-based therapy for supporting implantation in cases of recurrent miscarriages.

The generation of hiTSCs from hPSCs or following OSKM pluripotency reprogramming has been recently described^3–9^, proposing one possible strategy of producing hiTSCs from mesenchymal cells. However, the production of hiTSCs from mesenchymal cells, independently of pluripotency or to a combination of factors that robustly induces pluripotency, has not been shown before.

Here, we demonstrate that transient expression of GATA3, OCT4, KLF4 and MYC (GOKM) is capable of directly converting male and female human fibroblasts into functional hiTSCs without acquiring a pluripotent state. We reveal that, although essential for mouse TSC circuitry^49^, SOX2 is dispensable for the induction of the hTSC state and that GATA3 together with OCT4 are the main drivers of hTSC identity. Consistent with previous surprising work^20^ that uncovered a central role for OCT4 in the establishment of the human trophoblast lineage, our work confirms that this role can be utilized for the generation of hiTSC from fibroblasts independent of its known function as a master regulator of pluripotency.

We show that GOKM target the chromatin differently than OSKM and that GOKM reprogramming is more directed toward the hTSC state. In contrast, OSKM predominantly target regions that are shared between hESCs and hbdTSCs, suggesting an explanation as for how OSKM are also capable of generating hiTSCs. One interesting observation is that GOKM demonstrate a greater chromatin opening activity at early stages of reprogramming than OSKM as assessed by the higher number of regions that are remodeled and defined along the somatic genome. In accordance with these data, GOKM are capable of generating hiTSCs more efficiently than OSKM.

Methylation analysis revealed that the overwhelming majority of hbdTSC-specific methylated regions, compared to fibroblasts, underwent appropriate de novo methylation in the hiTSC clones. In contrast, a higher variation was seen between the various hiTSC clones in the hbdTSC-demethylated regions. Notwithstanding, one of the hiTSC clones clustered closer to hbdTSCs than to the other hiTSC clones, suggesting that near complete DNA methylation reprogramming is possible with GOKM reprogramming. Importantly, gatekeeper genes such as *ELF5* that are abnormally methylated during mouse ESC-TSC transdifferentiation are demethylated properly in the reprogrammed hiTSCs, similarly to the mouse model^12,23^.

It is important to note that appropriate rewiring of the methylation landscape during reprogramming is essential for both driving the reprogramming process and for acquiring the full epigenetic state of the targeted cells. Thus, we believe that improving demethylation capability by optimizing culture conditions and factor transduction will not only unleash the full functional potential of the cells, but will likely also increase the reprogramming efficiency. Devising simple methods such as gene expression markers to screen higher quality from lower quality reprogrammed colonies would also be helpful in obtaining optimal hiTSC lines.

Given recent literature showing the capacity to derive hiTSCs from pluripotent stem cells and reprogramming, and following the issues mentioned above regarding pluripotency and the hTSC state, it was important to confirm that the formation of GOKM- hiTSCs does not rely on obtaining a transient pluripotent state. By knocking out *SOX2* or *NANOG/PRDM14* in fibroblasts, we show that these factors, while essential for pluripotency induction, are dispensable for hTSC state acquisition.

Functional experiments validated hiTSC identity and demonstrated full developmental potential as assessed by capability to differentiate into STs and EVTs, form trophoblastic lesions in NOD/SCID mice and develop organoids in matrigel. We believe that the variations in EVT differentiation noted between different hiTSC colonies may be a result of the variation observed in the DNA methylation landscape of the cells. In support of this assumption, 5-Aza treatment facilitated EVT differentiation in some EVT-refractory treated clones. Alternative explanation is related to the expression of the *C19MC miRNA cluster* that was shown recently to be important for trophoblast differentiation^50^. In agreement with that, hiTSC#4 that demonstrated proper differentiation to EVTs also showed higher levels of some of these mIRs.

Overall, we describe here a system to produce fully functional hiTSCs from mesenchymal cells originating from either male or female, neonate or adult individuals. We show that stable GOKM-hiTSCs harbor a transcriptional advantage over OSKM-hiTSCs, which lack a gene signature associated with estrogen response and immune function. Though some calibration of culture and reprogramming conditions is likely needed, the advantages of a direct conversion approach are clear. We offer this reprogramming strategy as a valuable source to study diseases that are associated with pathological placental development.

## Methods

### Ethical considerations

This research was performed in compliance with the Ethic Committee of Shaare Zedek Medical Center, the joint ethics committee (IACUC) of the Hebrew University and Hadassah Medical Center and the National ethic committee (Israel health ministry) and NIH. The Hebrew University is an AAALAC international accredited institute.

### Derivation of human trophoblast stem cells from human blastocysts and human fibroblasts from skin biopsy and cell lines

The establishment and use of hbdTSC lines or hESC lines from PGD-derived embryos was performed in compliance with the protocols approved by the Ethics Committee of Shaare Zedek Medical Center (IRB 87/07). Embryo donations were carried out under the strict regulation of the National Ethics Committee (Israel Health Ministry) and NIH and ISSCR guidelines. The donated embryos are byproducts of PGD treatments and would otherwise have been destroyed. Complete separation was maintained between the individual who approached the patients and received informed consent for donation (genetic counselor), the attending physician (IVF gynecologist) and the researcher. While obtaining informed consent, the general aim of the research was explained to the patients. The patients were approached for donation only once and not every IVF cycle, to ensure that there was no connection between the signing of the informed consent form and medical treatment. There was no monetary compensation for the embryo donation.

In order to generate human blastocyst-derived TSC (hbdTSC) control lines, human blastocysts were plated on Mitomycin C-treated mouse embryonic fibroblast (MEF) feeder cells and cultured in human TSC medium (DMEM/F12 supplemented with 0.1 mM 2-mercaptoethanol, 0.2% FBS, 0.5% Penicillin-Streptomycin, 0.3% BSA, 1% ITS-X, 1.5 μg/ml L-ascorbic acid, 50 ng/ml EGF, 2 μM CHIR99021, 0.5 μM A83-01, 1 μM SB431542, 0.8 mM VPA and 5 μM Y27632) as previously described^1^. Following blastocyst outgrowth, the cells were trypsinized and transferred into new Mitomycin C-treated MEF feeder plates. The cells were passaged several times, until stable proliferative hbdTSCs emerged. PCS201 human primary fibroblasts were purchased from ATCC (PCS-201-012). GM2 female adult fibroblasts (GM25432) were a gift from Dr. Oren Ram (Hebrew University of Jerusalem). These cells were derived by Coriell INSTITUTE (https://catalog.coriell.org/) in compliance with their regulations. The derivation of KEN human fibroblasts from foreskin biopsy was performed in compliance with protocols approved by the Ethics Committee of Shaare Zedek Medical Center (IRB 88/11). hESCs was provided by Dr. Rachel Eiges and the breast cancer cell line MDA-MB-231 (HTB-26) was purchased from ATCC.

### Molecular cloning and hiTSC and hiPSC reprogramming

All dox-inducible factors were generated by cloning the open reading frame of each factor into the pMiniT2.0 vector (NEB) and then restricted with EcoRI or MfeI and inserted into the FUW-TetO expression vector. A lentiviral vector dox-dependent system was utilized for the transient expression of transcription factors. For infection, replication-incompetent lentiviruses containing the various reprogramming factors (GOKM 3:3:3:1, OSKM 3:3:3:1 and GSKM 3:3:3:1) were packaged with a lentiviral packaging mix (psPAX2 and pDGM.2 1:1) in HEK 293T cells (CRL-3216, embryonic human kidney, ATCC) and collected 48 h after transfection. The supernatants were filtered through a 0.45 μm filter, supplemented with 8 μg/ml of polybrene, and then used to infect fibroblasts which were previously infected with lentiviral vectors encoding puromycin resistance and M2rtTA, and subsequently selected with 2 µg/ml puromycin for 3–5 days. Twelve hours following the fourth infection, medium was replaced with basic reprogramming medium (BRM) consisting of fresh DMEM containing 10%FBS, 1% L-glutamine and 1% penicillin-streptomycin.

For hiTSC reprogramming, 6 h after medium replacement 2 μg/ml doxycycline (dox) was added to the medium. The reprogramming medium was changed every other day. After 14 days in BRM with dox, the medium was replaced with 50% BRM and 50% hTSC medium^1^ with dox, followed by 7 days in hTSC medium with dox. Then, dox was removed and colonies were allowed to stabilize for 7–10 days. Plates were then screened for primary hiTSC colonies. Each colony was isolated by manually picking up colonies with a pipette, trypsinized with TrypLE (Gibco) and plated in a separate well on feeder cells in hTSC medium. The cells were passaged several times until stable proliferative hiTSC colonies emerged.

For hiPSC reprogramming, 6 h after medium replacement 2 μg/ml doxycycline (dox) was added to the medium. The reprogramming medium was changed every other day. After 7 days in BRM, the medium was replaced with 50% BRM and 50% human embryonic stem cell (hESC) medium comprised of Knockout DMEM containing 15% KnockOut serum replacement, 0.1 mM 2-mercaptoethanol, 1% L-glutamine, 1% non-essential amino acids and 1% Penicillin-Streptomycin, with dox, followed by 7 additional days in hESC medium with dox. Then, dox was removed and colonies were allowed to stabilize for 7–10 days in hESC medium supplemented with freshly added 4 ng/ml bFGF. Each colony was isolated by manually cutting colonies into small chunks with a Pasteur pipette and manually transferring each with a pipette to a separate well on feeder cells in hESC medium supplemented with 4 ng/ml bFGF freshly added to each well.

### Generation of non-integrating episomal-derived hiTSCs

Episomal vectors: pCLXE-hOCT4-shp53, pCXWB-EBNA1, pCLXE-hL-MYC/LIN28A vectors used for this study were originally described in Okita et al.^39^, Okita et al.^51^ and were obtained from Addgene (Cat 27078, Cat 37624, and Cat 27077, respectively). pCXLE-hGATA3 and pCXLE-hKLF4 vectors were generated by subcloning the open reading frame of either hGATA3 or hKLF4 into pCXLE vector using EcoRI enzyme. For electroporation, fibroblasts were trypsinized and 3.5 × 10^5^ cells were resuspended in 100 µl of R buffer (Life Technologies, Carlsbad, CA, USA). Subsequently, 1 µg/100 µl of each of the episomal plasmids (pCXLE-hGATA3, pCXLE-hKLF4, pCXLE-hUL) with 1.5 µg/100 µl of pCXLE-hOCT4-shp53 and 0.5 µg/100 µl of pCXWB-EBNA1 episomal plasmids were added to the cell suspension. Electroporation was carried out with the NEON transfection system according to the manufacturer’s instructions (Life Technologies; 1650 V, 20 ms, 1 pulse). Subsequently, cells were seeded onto 6-well plates containing basic reprogramming medium (BRM) consisting of fresh DMEM containing 10%FBS, 1% L-glutamine, and 1% penicillin-streptomycin. For hiTSC reprogramming, the fibroblasts were cultured in BRM for 10 days, and then the medium was exchanged to hTSC medium (Okae et al.^1^) for another 14 days. Plates were then screened for primary hiTSC colonies. Each colony was isolated by manually picking up colonies with a pipette, trypsinized with TrypLE (Gibco), and plated in a separate well on feeder cells in hTSC medium. The cells were passaged several times until stable proliferative hiTSC colonies emerged.

### Quantitative PCR (qPCR) for mRNA expression and analysis of genomic integration of transgenes

For analysis of mRNA expression using qPCR, total RNA was isolated using the Macherey-Nagel kit (Ornat). In total, 500–1000 ng of total RNA was reverse transcribed using iScript cDNA Synthesis kit (Bio-Rad). Quantitative PCR analysis was performed in duplicates using 1/100 of the reverse transcription reaction in a StepOnePlus (Applied Biosystems) with SYBR green Fast qPCR Mix (Applied Biosystems). Specific primers were designed for the different genes (see Supplementary Table 1). All quantitative real-time PCR experiments were normalized to the expression of *GAPDH* and presented as a mean ± standard deviation of two duplicate runs.

For analysis of integration of transgenes into genomic DNA using qPCR, genomic DNA was isolated by incubating trypsinized cell pellets in lysis buffer consisting of 100 mM Tris pH8, 5 mM EDTA, 0.2% SDS and 200 mM NaCl overnight with 400 μg/ml proteinase K (Axxora) at 37 °C for 1 h followed by incubation at 55 °C for 1 h. Then, genomic DNA was precipitated with isopropanol, washed with 70% ethanol and resuspended in ultra-pure water (BI). Forward primers for the end of the last exon of cloned genes were used in conjunction with reverse primers for the FUW-tetO vector at the region immediately downstream of the cloned gene (see Supplementary Table 1). Results were normalized to an intronic region of the *GAPDH* gene and presented as a mean ± standard deviation of two duplicate runs.

### qRT-PCR of miRNA

Cells of four hiTSC colonies, hbdTSC, hESCs and breast cancer cell line MDA-MB-231 were lysed and total RNA was isolated using the Macherey-Nagel kit (Ornat). To quantify C19MC miRNAs, we adapted a previously published method^52^. Briefly, RNA (10 ng) in 15 ml reaction mixture was converted into cDNA using RT primers (50 nM) that were complementary to each miRNA with a TaqMan MicroRNA Reverse Transcription Kit (Life Technologies #4366596). Primers were adapted from (Lee et al.^23^). The cDNAs were quantified by qRT-PCR with qPCRBIO Fast qPCR SyGreen (Tamar # PB20.16). hsa-miR-103a was used for normalization of the results^23^.

### Immunostaining of PFA-fixated cells and flow cytometry

Cells were fixed in 4% paraformaldehyde (in PBS) for 20 min, then rinsed 3 times with PBS and blocked for 1 h with PBS containing 0.1% triton X-100 and 5% FBS. The cells were incubated overnight with primary antibodies in 4 C. The antibodies are: anti-KRT7 (Abcam, ab215855, 1:200), anti-GATA3 (Abcam, ab106625, 1:200), anti-GATA2 (Abcam, ab173817, 1:200), anti-TFAP2C (Santa Cruz Biotechnologies, sc-12762, 1:200), anti-KRT18 (Santa Cruz Biotechnologies, sc-51582, 1:200), anti-CDH1 (Santa Cruz Biotechnologies, sc-7870, 1:200), anti-VIM (Cell Signaling Technology, #5741, 1:200), anti-SDC1 (Abcam, ab128936, 1:200), anti-CSH1 (Abcam, ab15554, 1:200), anti-HLA-G (Abcam, ab52455, 1:200), anti-ITGA5 (Abcam, ab150361, 1:200), anti-SOX2 (Abcam, ab97959, 1:200), anti-OCT4 (Abcam, ab19857, 1:200), anti-TRA-1-60 (Abcam, ab16288, 1:200) diluted in PBS containing 0.1% triton X-100 and 1% FBS. The next day, the cells were washed 3 times with PBS, and incubated for 1 h with relevant (Alexa) secondary antibody (Abcam, Cat#ab150112; Abcam, Cat#ab150065; Invitrogen, Cat#A21202; 1:500 dilution) in PBS containing 0.1% triton X-100 and 1%FBS. DAPI was added 10 min before end of incubation. Negative control included incubation with secondary antibody without primary.

For flow cytometry analysis of HLA class I and TRA-1-60 expression, cells were trypsinized and blocked for 10 min in incubation buffer containing 0.5% bovine serum albumin (BSA) (Sigma-Aldrich) in PBS. Then, cells were centrifuged and resuspended in incubation buffer with anti-HLA class I (1:300, Abcam, ab22432) or anti-TRA-1-60 (1:300, Abcam, ab16288) for 1 h. Cells were then washed with incubation buffer and incubated for 30 min with relevant (Alexa) secondary antibody (Invitrogen, Cat#A21202, 1:500), after which cells were washed, resuspended in incubation buffer, filtered through mesh and analyzed and/or sorted. HLA class I stained cells were analyzed by Beckman Coulter (Gallios) flow cytometer using the Kaluza Software (V 1.0.14029.14028). TRA-1-60 stained cells were sorted using FACSAria III (BD Biosciences) and analyzed using BD FACSDiva (V 8.0.1).

### RNA and RRBS library preparation and sequencing and karyotype analysis

For RNA-seq, total RNA was isolated using the Qiagen RNeasy kit. mRNA libraries were prepared using the SENSE mRNA-seq library prep kit V2 (Lexogen), and pooled libraries were sequenced on an Illumina NextSeq 500 platform to generate 75-bp single-end reads.

For RRBS, DNA was isolated from samples and incubated in lysis buffer (25 mM Tris-HCl at pH8, 2 mM EDTA, 0.2%SDS, 200 mM NaCl) supplemented with 300 μg/ml proteinase K (Roche) followed by phenol:chloroform extraction and ethanol precipitation. hiTSC colonies and hbdTSC colonies were passaged twice on matrigel in order to eliminate the presence of MEF feeder cells. RRBS libraries were prepared as previously described^53^. Samples were run on HiSeq 2500 (Illumina) using 100 bp paired-end sequencing.

Karyotype analysis was performed on identical isolated DNA samples using Affymetrix CytoScan 750 K array.

### Chromatin immunoprecipitation (ChIP)

Chromatin immunoprecipitation (ChIP) assay was performed as previously described^54^. Briefly, cells from two biological replicates per line were fixed for 10 min at RT with a final formaldehyde concentration of 0.8%. Formaldehyde was quenched with glycine at a final concentration of 125 mM. The cells were then lysed with lysis buffer (100 mM Tris-HCl, 300 mM NaCl, 2% Triton X-100, 0.2%v sodium deoxycholate and 10 mM Cacl2) supplemented with EDTA-free protease inhibitor (Roche, 11873580001) for 20 min on ice. The chromatin was digested by adding MNase (Thermo Scientific, 88216) for 20 min at 37 °C and MNase was inactivated by adding 20 mM EGTA. The fragmented chromatin was added to pre-bounded Dynabeads (A and G mix, Invitrogen, 10004D/ 10002D) using H3K4me2 antibody (Millipore, 07-030) at 2 μg per reaction. Samples were then washed twice with RIPA buffer, twice with RIPA high salt buffer (NaCl 360 mM), twice with LiCl wash buffer (10 mM Tris-Hcl, 250 mM LiCl, 0.5% DOC, 1 mM EDTA, 0.5% IGEPAL) and twice with 10 mM Tris-HCl pH = 8. DNA was purified by adding RNase A (Thermo Scientific, EN0531) and incubated for 30 min at 37 °C and then with Proteinase K (Invitrogen, 25530049) for 2 h. The DNA was eluted by adding 2X concentrated elution buffer (10 mM Tris-HCl, 300 mM NaCl, 1% SDS, 2 mM EDTA) and reverse crosslinked overnight at 65 °C. The DNA was then extracted using AMPure XP beads (Beckman Coulter Genomics, A63881). ChIP sample libraries were prepared according to Illumina Genomic DNA protocol.

### ATAC libraries and sequencing

ATAC-Seq library preparation was performed as previously described^55^. Briefly, 50,000 cells per replicate (two biological replicates per line) were incubated with 0.1% NP-40 to isolate nuclei. Nuclei were then transposed for 30 min at 37 °C with adapter-loaded Nextera Tn5 (Illumina, Fc-121-1030). Transposed fragments were directly PCR amplified and Sequenced on an Illumina NextSeq 500 platform to generate 2 × 36-bp paired-end reads.

### Differentiation of hiTSCs

For directed differentiation into ST, ~10^5^ cells were seeded on Matrigel coated 12-well plates at a concentration of 1:30 in ambient oxygen conditions in a medium consisting of DMEM/F12 supplemented with 0.1 mM 2-mercaptoethanol, 0.5% Penicillin-Streptomycin, 0.3% BSA, 1% ITS supplement, 2.5 μM Y27632, 2 μM forskolin, and 4% KSR, as described^1^. Cells were collected at day 2 and 6 for analysis of mRNA expression using qPCR as described above. Cells were also seeded on 12-well plates at a density of ~10^5^ cells per plate, cultured similarly and fixated in 4% PFA for immunostaining as described above.

For directed differentiation into EVT, ~4 × 10^5^ cells were seeded on Matrigel coated 12-well plates at a concentration of 1:100 in ambient oxygen conditions in a medium consisting of DMEM/F12 supplemented with 0.1 mM 2-mercaptoethanol, 0.5% Penicillin-Streptomycin, 0.3% BSA, 1% ITS supplement, 100 ng/ml NRG1, 7.5 μM A83-01, 2.5 μM Y27632, and 4% KnockOut Serum Replacement, as described by ref. ^1^. Matrigel was added to a final concentration of 2%. At day 3, the medium was replaced with the EVT medium without NRG1, and Matrigel was added to a final concentration of 0.5%. Medium was replaced every other day, and cells were collected at day 7 and 14. Cells were also fixated in 4% PFA for immunostaining as described above.

### Formation of trophoblast organoids with bdTSCs and hiTSCs

Similar to as described in ref. ^41^, hbdTSCs and hiTSCs were suspended in trophoblast organoid medium (TOM) consisting of DMEM/F12, 10 mM HEPES, 1 × B27, 1 × N2, 1 mM L-glutamine, 100 ng/ml R-spondin, 1 μM A83-01, 100 ng/ml recombinant human epidermal growth factor (rhEGF), 50 ng/ml recombinant murine hepatocyte growth factor (rmHGF), 2.5 μM prostaglandin E2, 3 μM CHIR99021, and 100 ng/ml Noggin. Growth factor-reduced Matrigel (GFR-M) was added to reach a final concentration of 60%. Solution (40 μl) containing 10^4^−10^5^ hbdTSCs/hiTSCs was placed in the center of 24-well plates. After 2 min at 37 °C, the plates were turned upside down to ensure equal spreading of the cells in the solidifying GFR-M-forming domes. After 15 min, the plates were turned again and the domes were carefully overlaid with 500 μl prewarmed TOM. Cells were cultured in 5% oxygen for 10–19 days and then subject to immunostaining.

### Immunostaining of hbdTSC and hiTSC trophoblast organoids

Organoid-containing Matrigel domes were fixated in 4% PFA overnight. Then, domes were washed with PBS for 15 min twice. Domes were submerged in blocking solution containing 3% bovine serum albumin (BSA), 5% fetal bovine serum (FBS), 0.1% Triton X-100 in PBS, at 4 °C overnight. Then, tissues were incubated with primary antibodies including anti-Ki67 (1:200 Abcam, ab15580), anti-KRT7 (1:200, Abcam, ab215855), anti-CSH1 (1:200, Abcam, ab15554) and anti-TFAP2C (1:100, Santa Cruz Biotechnologies, sc-12762) diluted in PBS containing 1% BSA and 0.1% Triton X-100, on a rocking plate at 4 °C for two nights. Plates were moved to room temperature and continued rocking for at least 2 additional hours before washing in PBS containing 0.1% Triton X-100 overnight, with at least 5 changes of buffer. Following this, domes were incubated in secondary antibody solution containing relevant (Alexa) secondary antibody (1:200) diluted in 1% BSA and 0.1% Triton X-100 on a rocking plate at 4 °C overnight. Then, domes were washed again with PBS containing 0.1% Triton X-100 overnight, with at least 5 changes of buffer. Domes were then incubated with DAPI for 1 h and stored in PBS in 4 °C until imaging. Imaging was performed using spinning disk confocal microscopy with Nikon Eclipse Ti2 CSU-W1 Yokogawa confocal scanning unit, Andor Zyla sCMOS camera and Nikon Plan Apo VC 20X NA 0.75 lens. Maximal intensity projection images were created using NIS-Elements microscope imaging software.

### Engraftment of hiTSCs into NOD/SCID mice and immunohistochemistry (IHC)

Mouse (Mus musculus) NOD/SCID male, ~12 weeks of age were used. The animals were housed in the Ein-Karem Faculty of Medicine and Hadassah Medical Center Animal House and all housing conditions were overseen by the Hebrew University Authority for Biological and Biomedical Models (ABBM). The joint ethics committee (IACUC) of the Hebrew University and Hadassah Medical Center approved the study protocol (IACUC# MD-18-15628-3) for animal welfare. The Hebrew University is an AAALAC international accredited institute.

For each lesion, ~4 × 10^6^ were trypsinized with TrypLE, washed twice in PBS, resuspended in 150 μl of a 1:2 mixture of Matrigel and PBS and subcutaneously injected into NOD/SCID mice.

Lesions were collected 9 days after injection, dissected, fixed in 4% paraformaldehyde overnight, embedded in paraffin, sectioned and mounted onto slides. Some slides were stained with H&E, while others were subject to IHC staining.

For IHC, slides were deparaffinized in xylene and rehydrated in a decreasing ethanol gradient. Antigen retrieval was performed in a sodium citrate buffer and slides were heated for 3 min at 110–120 °C. After a short incubation in 3% hydrogen peroxide, sections were incubated overnight in CAS-block (Invitrogen) with primary antibodies anti-KRT7 (1:1000, Abcam, ab215855), HLA-G (1:100, Abcam, ab52455) and anti-CSH1 (1:100, Abcam, ab15554). Then, sections were incubated with appropriate HRP-conjugated secondary antibody (Vector Laboratories) for 30 min and immunohistochemistry was performed using DAB peroxidase substrate kit (Vector Laboratories). Slides were lightly counterstained with hematoxylin.

### hCG detection in the blood of hiTSC-injected mice

For testing presence of hCG in NOD/SCID mice, ~500 μl of mouse serum was collected in Eppendorf tubes by collecting whole blood and centrifuging at 1500 G for 10 min at 4 °C to separate serum. Serum samples were stored at −80 °C until processing.

Quantification of human chorionic gonadotropin (hCG) hormone levels were determined by ELISA kit by Alpco (Almog diagnostic #25-HCGHU-E01), following the manufacturer’s protocol.

### ELF5 bisulfite sequencing

ELF5 bisulfite sequencing was performed as previously published^23^. DNA from each sample was treated with bisulfite using the EpiTect Bisulfite Kit (Qiagen #59110), according to the manufacturer’s protocol. In total, 10% of the resulting DNA was used for the amplification of the 432 to 3 bp region upstream of the ELF5 start site via nested PCR. Amplicons were directly sequenced using MiSeq 2 × 150 bp paired-end run.

### Generation of SOX2 and NANOG/PRDM14 KO hiTSCs

gRNAs designed to target the first exon of SOX2, NANOG and PRDM14 (see Supplementary Table 1) were cloned into pLentiCRISPR V2 vectors with puro (SOX2, NANOG) or hygromycin B (PRDM14) resistance (Addgene plasmids #52961 and #98291 respectively) using BsmBI restriction enzyme, as described in ref. ^46^. These were used to infect HFFs (KEN), which were previously infected with lentiviral vectors encoding for GFP and M2rtTA. Next, cells were selected with 2 µg/ml puromycin for 3–5 days (SOX2-KO), or underwent double selection with both 2 µg/ml puromycin for 3–5 days and 200 ug/ml hygromycin B for 5–7 days (NANOG/PRDM14-DKO). Identical fibroblasts infected with pLentiCRISPR V2 vectors without gRNA and similarly selected were used as controls. Cells were then subjected to reprogramming to hiTSCs or hiPSCs using GOKM or OSKM factors, as described above. For analysis of the presence of genomic indels, as well as analysis of mouse vs. human SOX2 integration and expression, colonies were passaged at least twice on Matrigel 1:30 without the presence of MEF feeder cells. For assessment of indel events in hiTSCs resulting from the SOX2-KO HFF reprogramming, genomic DNA was isolated as described above and analyzed using PCR and running products on agarose gel, or qPCR with specific primer pairs (see Fig. 7b and Supplementary Table 1). For assessment of indel events in hiTSCs resulting from the NANOG/PRDM14-DKO HFF reprogramming, colonies were subjected to Sanger sequencing at the NANOG and PRDM14 gene loci (HAI Laboratories) and visualized using Sequencher software.

### Quantification and statistical analysis

Two-tailed unpaired *t-*test was used for experiments comparing differences between two groups. Statistical significance differences were considered when *p* value ≤ 0.05. For EnrichR GO term analysis, *p* value was calculated using Fisher exact test and for HOMER binding motif analysis, *p* value was calculated using the binomial distributions. All experiments were repeated at least three times, unless specified otherwise.

### RNA-seq analysis

Poly-A/T stretches, and Illumina adapters were trimmed from the reads using cutadapt (10.14806/ej.17.1.200); resulting reads shorter than 30 bp were discarded. Reads for each sample were aligned independently to the H. sapiens reference genome GRCh38 using STAR^56^. Counting proceeded over genes annotated in Ensembl using htseq-count^57^. Only uniquely mapped reads were used to determine the number of reads that map to each gene (intersection-strict mode). Differential analysis was performed using DESeq2^58^ package in R with the betaPrior, cooksCutoff, and independentFiltering parameters set to False. Raw *p* values were adjusted for multiple testing using the procedure of Benjamini and Hochberg. Differentially expressed (DE) genes were determined by a *p* adj of <0.05, absolute fold changes >2, and a count of at least 30 in at least one sample.

PCA analysis was done on the 1000 most variable genes between all samples.

Additional samples of hiTSC were added to the analysis. The relevant count matrixes were downloaded from^3,9^. The normalized counts of all samples were adjusted using the limma^59^ removeBatchEffect function for downstream visualization.

In total, 201 DE genes specific to hbdTSC with logFC > 6 when compared to both hiPSC and fibroblasts cells were selected, and their expression were plotted in the other samples. The same analysis was done for 289 DE genes specific to hiPSC.

### DNA methylation analysis

For the analysis of RRBS data, raw reads (FastQ files) were quality-trimmed using Trim Galore (v 0.6.5, default parameters) and aligned to the human genome GRCh38 using BSMAP (v 2.9^60^). The methylation ratio of CpGs with sequencing depth of at least 10 reads were computed based on 100 bp tiles. Differentially methylated regions (DMR) table obtained from Methylkit (v 1.14.2: 10.18129/B9.bioc.methylKit) processing of the BAM files yielded by BSMAP alignment. Each table represents the following parameters: Chromosome (chr), start and end coordinates of the methylated region (start, end), strand location (strand), probability value “pvalue”, adjusted *p* value “qvalue”, differential methylation score “meth.diff”. Only regions with a meth.diff score over 50 or under −50 and a *q* value under 1E−5 where considered as differentially methylated (hyper- or hypo-methylated respectively).

For the analysis of direct amplification and sequencing of the *ELF5 promoter a* FATSTA file that contains ~20 kbp from the human genome GRCh38 upstream of ELF5 (chr11:34496606-34517332) was constructed. An index for the ELF5 FASTA file was constructed using the function “bismark_genome_preparation” from the Bismark (v 0.22.3^61^). Alignment was then performed using bismark --bowtie2^62^ and methylation. Finally, methylation information was extracted using the function “bismark_methylation_extractor”.

### ATAC-seq and ChIP-seq analysis

For ATAC-seq, the cutadapt (10.14806/ej.17.1.200) tool was used to trim adapters using the following sequence: CTGTCTCTTATACACATCT from both reads. Additional 3 bases were trimmed from the end of the read. Reads which were shorter than 25 bases after the trimming were discarded. The trimmed reads were aligned to the H. sapiens reference genome GRCh38 using bowtie^62^ (version 1.0.0), using the options --maxins 2000, -m 1, best, strata, and -n 1. Reads that were mapped to the mitochondrial chromosome were excluded. Duplicate reads were removed using Picard (https://broadinstitute.github.io/picard/) (version 2.3.0) MarkDuplicates command. Peaks were called using MACS2^63^ with --nomodel --shift −100 --extsize 200 parameters. Peaks from unknown contigs and blacklist regions were removed from the analysis.

For ChIP-seq, H3K4me2 data of hESC was downloaded from GSE16256.

Illumina adapters were trimmed from the reads using cutadapt (10.14806/ej.17.1.200). Reads which were shorter than 25 bases after the trimming were discarded. The trimmed reads were aligned to the H. sapiens reference genome GRCh38 using bowtie^62^ (version 1.0.0), using the options -m 1 --phred33-quals −5 0 −3 0 --best -n 1 -l 28 --strata. Reads that were mapped to the mitochondrial chromosome were excluded. Peaks were called using MACS2^63^ with --nomodel --broad parameters. Peaks from unknown contigs and blacklist regions were removed from the analysis.

Peaks from all samples were merged using bedtools^64^ merge (version 2.29.2) command. R csaw package^65^ was used to count reads on the merged peaks. EdgeR^66^ package in R was used for normalization and differential peak analysis. Peaks were defined specifically for each group of cells and the R package (VennDiagram) was used to construct all Venn diagrams. Differential peaks were determined by FDR < 0.05 and absolute log2 fold changes >1. For GOKM and OSKM differential ATAC-seq analysis, DA peaks were determined with the same criteria as above, with the additional filter of unique peaks (peaks that were called as peaks using MACS2 only in one of the samples).

Association between peaks and genes was done using GREAT^67^ with the Basal plus extension option with distal up to 150 kb. Extracted genes were then intersected with a specific set of genes that were differentially expressed in hbdTSCs and not in fibroblasts or hESCs, or set of genes that were differentially expressed in hESCs and not in fibroblasts or hbdTSCs.

Motif analysis on specific sets of peaks was done using Homer^68^ findMotifsGenome.pl with --size given parameter. The summits of the peaks were extended ±100 bp and these intervals were used for the motif analysis.

### Reporting summary

Further information on research design is available in the Nature Portfolio Reporting Summary linked to this article.

## Supplementary information

Supplementary Information

Reporting Summary

## Data Availability

All RNA-seq, RRBS, ATAC-seq and ChIP-seq data were deposited in the Gene Expression Omnibus database (GEO) under accession number GSE182017. All analyses used UCSC hg38 human reference genome. The figures that are associated with the raw data files are: Figs. 2a, b, d, 3a–n, 4a–d, f, 5a–j and S2a–e, S3a–j, S4a–k, S5a–k, S9a–h and S10. Remaining data are provided within the Article, Supplementary Information. Source data are provided with this paper.

## Source data

Source Data
