# Supplementary material for: Pluripotency-independent induction of human trophoblast stem cells from fibroblasts
Source: Nat Commun. 2023 Jun 8;14:3359. doi: 10.1038/s41467-023-39104-1 (PMC10250329; doi:10.1038/s41467-023-39104-1)
Supplement: Supplementary file 1 — Supplementary Information [file 41467_2023_39104_MOESM1_ESM.pdf]

SUPPLEMENTARY DATA

Pluripotency-Independent Induction of Human Trophoblast Stem Cells from Fibroblasts

Moriyah Naama[1,6], Moran Rahamim[1,6], Valery Zayat[2], Shulamit Sebban[1], Ahmed Radwan[1], Dana Orzech[1], Rachel Lasry[1], Annael Ifrah[1], Mohammad Jaber[1], Ofra Sabag[1], Hazar Yassen[1], Areej Khatib[1], Silvina Epsztejn-Litman[3,4], Michal Novoselsky-Persky[5], Kirill Makedonski[1], Noy Deri[1], Debra Goldman-Wohl[5], Howard Cedar[1], Simcha Yagel[5], Rachel Eiges[3,4] & Yosef Buganim[1,#]

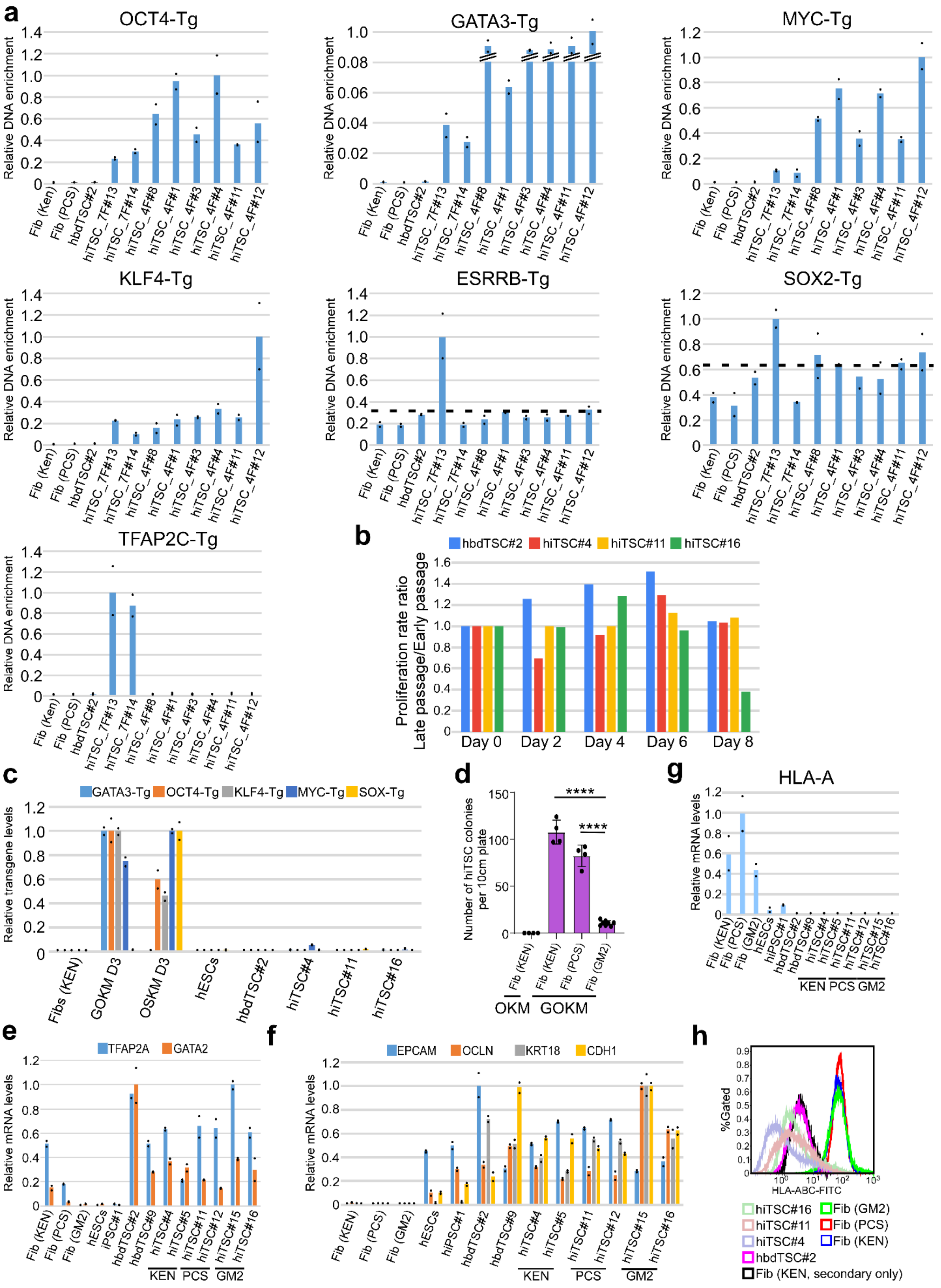
a
OCT4-Tg
GATA3-Tg
MYC-Tg
KLF4-Tg
ESRRB-Tg
SOX2-Tg
TFAP2C-Tg
Relative DNA enrichment
Fib (Ken)
Fib (PCS)
hbdTSC#2
hiTSC_7F#13
hiTSC_7F#14
hiTSC_4F#8
hiTSC_4F#1
hiTSC_4F#3
hiTSC_4F#4
hiTSC_4F#11
hiTSC_4F#12
b
hbdTSC#2
hiTSC#4
hiTSC#11
hiTSC#16
Proliferation rate ratio
Late passage/Early passage
Day 0
Day 2
Day 4
Day 6
Day 8
c
GATA3-Tg
OCT4-Tg
KLF4-Tg
MYC-Tg
SOX-Tg
Relative transgene levels
Fibs (KEN)
GOKM D3
OSKM D3
hESCs
hbdTSC#2
hiTSC#4
hiTSC#11
hiTSC#16
d
Number of hiTSC colonies per 10cm plate
****
****
Fib (KEN)
Fib (KEN)
Fib (PCS)
Fib (GM2)
OKM
GOKM
g
HLA-A
Relative mRNA levels
Fib (KEN)
Fib (PCS)
Fib (GM2)
hESCs
hiPSC#1
hbdTSC#2
hbdTSC#9
hiTSC#4
hiTSC#5
hiTSC#11
hiTSC#12
hiTSC#15
hiTSC#16
KEN
PCS
GM2
e
TFAP2A
GATA2
Relative mRNA levels
Fib (KEN)
Fib (PCS)
Fib (GM2)
hESCs
iPSC#1
hbdTSC#2
hbdTSC#9
hiTSC#4
hiTSC#5
hiTSC#11
hiTSC#12
hiTSC#15
hiTSC#16
KEN
PCS
GM2
f
EPCAM
OCLN
KRT18
CDH1
Relative mRNA levels
Fib (KEN)
Fib (PCS)
Fib (GM2)
hESCs
hiPSC#1
hbdTSC#2
hbdTSC#9
hiTSC#4
hiTSC#5
hiTSC#11
hiTSC#12
hiTSC#15
hiTSC#16
KEN
PCS
GM2
h
%Gated
HLA-ABC-FITC
hiTSC#16
hiTSC#11
hiTSC#4
hbdTSC#2
Fib (GM2)
Fib (PCS)
Fib (KEN)
Fib (KEN, secondary only)

Figure S1. Fibroblasts reprogrammed to hiTSCs with GOKM undergo MET and express hTSC markers. (a) qPCR analysis of the indicated transgenes in hiTSC colonies and negative controls. The highest sample for each transgene was set to 1. Results were normalized to an intronic region of *GAPDH* and are shown as fold change. For each sample two replicates (n=2) were used. Dashed lines mark the negative threshold of the various transgenes based on uninfected cells. **(b)** Graph depicting the differences in the proliferation rate of early (~5) and late passage (~30) hbdTSCs in 3 independent hiTSC clones (n=3) over 8 days. Although we observed differences in the proliferation rate between clones, only mild differences were seen between early and late passage. **(c)** qPCR analysis of mRNA levels of the indicated transgenes in various hiTSC colonies and in GOKM or OSKM transduced fibroblasts following 3 days of dox. The highest sample for each transgene was set to 1. Results were normalized to the mRNA levels of *GAPDH* and are shown as fold change. For each sample two replicates (n= 2) were used. hiTSC colonies were derived from 3 independent reprogramming experiments (n= 3). **(d)** Graph showing an average number of hiTSC colonies generated by OKM or GOKM in the indicated fibroblast lines. Error bars indicate standard deviation between 4 independent experiments/replicates (n= 4, for GM2 n=8). **** indicates p-value < 0.0001 (95% confidence interval -107.3 to -87.18 for Fib (KEN) vs Fib (GM2) and -81.12 to -62.88 for Fib (KEN) vs Fib (PCS), using 2-tailed unpaired t test calculated by GraphPad Prism (8.3.0). Mean values (from left to right) are: 0.000, 107.8, 82.50, 10.50. **(e-g)** qPCR analysis of mRNA levels of trophoblast markers *GATA2* and *TFAP2A* (e), epithelial markers *EPCAM, OCLN, KRT18, CDH1* (f) and *HLA-A* (g) in the indicated samples. The highest sample for each gene was set to 1. Results were normalized to the mRNA levels of *GAPDH* and are shown as fold change. For each sample two replicates (n =2) were used. **(h)** Flow cytometry histogram of HLA-A/B/C in the indicated samples using W6/32 antibody. Source data are provided as a Source Data file.

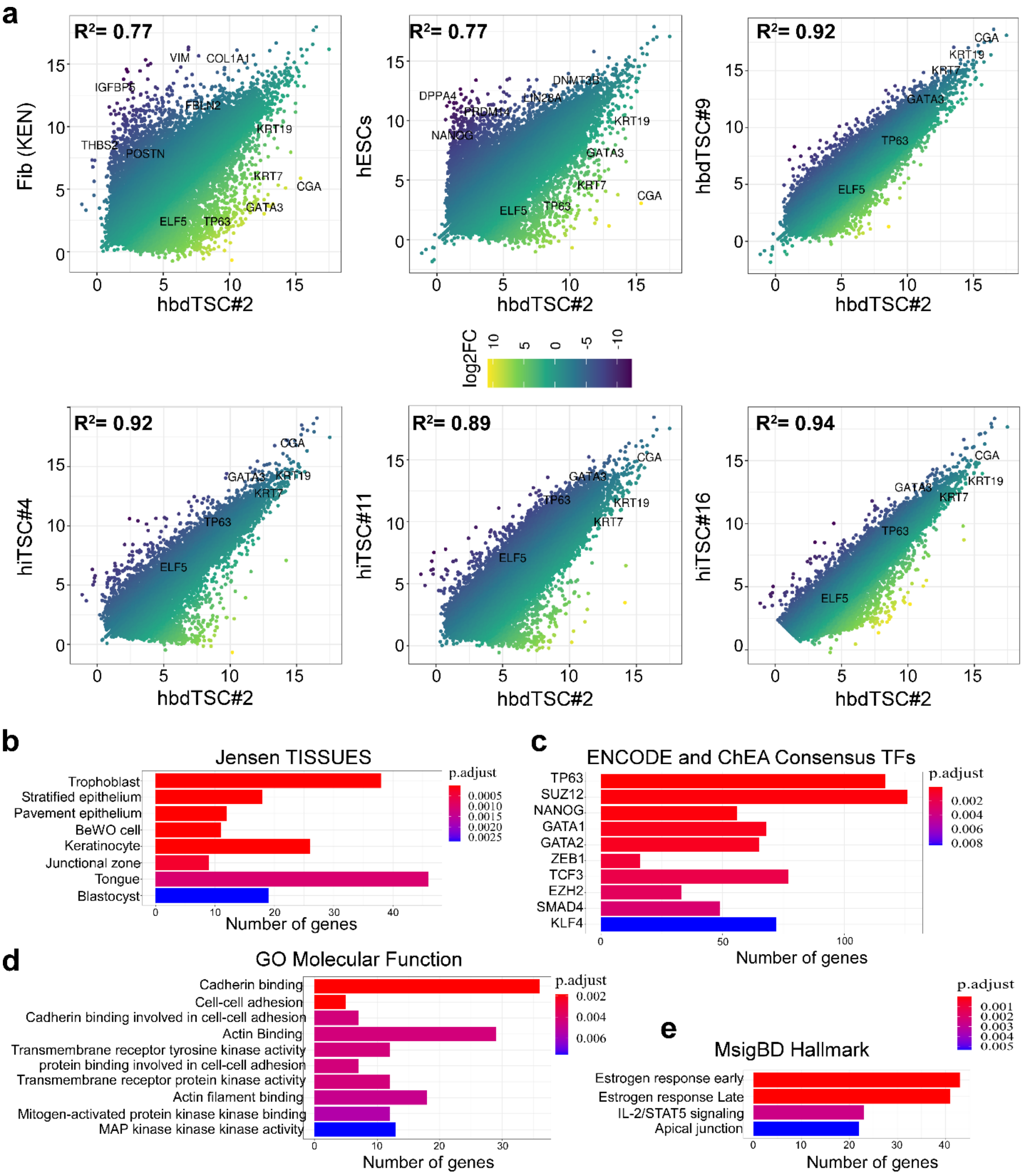

a
R²= 0.77
Fib (KEN)
hbdTSC#2
VIM
COL1A1
IGFBP5
FBLN2
KRT19
THBS2
POSTN
KRT7
CGA
GATA3
ELF5
TP63
R²= 0.77
hESCs
hbdTSC#2
DPPA4
DNMT3B
LIN28A
PRDM14
NANOG
KRT19
GATA3
KRT7
CGA
ELF5
TP63
R²= 0.92
hbdTSC#9
hbdTSC#2
CGA
KRT19
KRT7
GATA3
TP63
ELF5
log2FC
10 5 0 -5 -10
R²= 0.92
hiTSC#4
hbdTSC#2
CGA
GATA3
KRT19
KRT7
TP63
ELF5
R²= 0.89
hiTSC#11
hbdTSC#2
CGA
GATA3
TP63
KRT19
KRT7
ELF5
R²= 0.94
hiTSC#16
hbdTSC#2
CGA
GATA3
KRT19
KRT7
TP63
ELF5
b
Jensen TISSUES
Trophoblast
Stratified epithelium
Pavement epithelium
BeWO cell
Keratinocyte
Junctional zone
Tongue
Blastocyst
Number of genes
p.adjust
0.0005
0.0010
0.0015
0.0020
0.0025
c
ENCODE and ChEA Consensus TFs
TP63
SUZ12
NANOG
GATA1
GATA2
ZEB1
TCF3
EZH2
SMAD4
KLF4
Number of genes
p.adjust
0.002
0.004
0.006
0.008
d
GO Molecular Function
Cadherin binding
Cell-cell adhesion
Cadherin binding involved in cell-cell adhesion
Actin Binding
Transmembrane receptor tyrosine kinase activity
protein binding involved in cell-cell adhesion
Transmembrane receptor protein kinase activity
Actin filament binding
Mitogen-activated protein kinase kinase binding
MAP kinase kinase kinase activity
Number of genes
p.adjust
0.002
0.004
0.006
e
MsigBD Hallmark
Estrogen response early
Estrogen response Late
IL-2/STAT5 signaling
Apical junction
Number of genes
p.adjust
0.001
0.002
0.003
0.004
0.005

Figure S2. RNA-seq analysis indicates that hiTSCs have a transcriptome enriched for gene ontology terms related to placental development. (a) Scatter plots displaying pairwise correlations of gene expression levels for hbdTSC#2 vs KEN fibroblasts, hbdTSC#2 vs hESCs, hbdTSC#2 vs hbdTSC#9, hbdTSC#2 vs hiTSC#4, hbdTSC#2 vs hiTSC#11, and hbdTSC#2 vs hiTSC#16. Two RNA-seq replicates were generated for differential gene expression analyses (log2FC in absolute value >1, p.adj < 0.05, and raw counts > 30 in at least one of the samples). Representative genes from each cell type are marked. R^2 value was calculated for each pairwise comparison, demonstrating a high degree of similarity between hiTSCs and hbdTSCs. **(b-e)** Bar graph showing the highest enriched GO terms for top 1000 most differentially expressed genes between hiTSCs and fibroblasts using different categories within EnrichR. adjusted p-value was calculated using Benjamini-Hochberg. Source data are provided as a Source Data file.

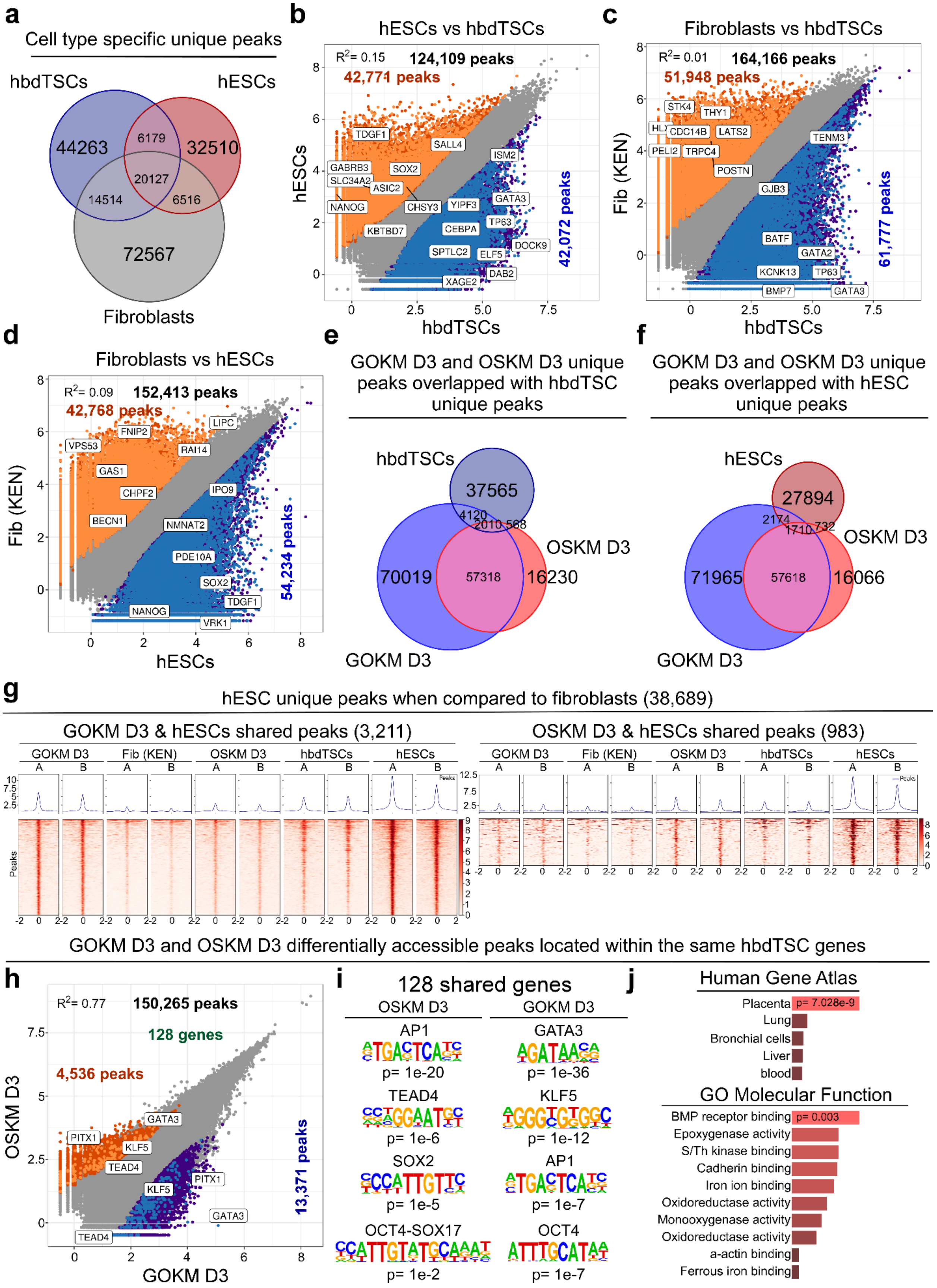
a
Cell type specific unique peaks
hbdTSCs
hESCs
Fibroblasts
44263
6179
32510
20127
14514
6516
72567
b
hESCs vs hbdTSCs
R^2= 0.15
124,109 peaks
42,771 peaks
42,072 peaks
hESCs
hbdTSCs
TDGF1
SALL4
ISM2
GABRB3
SOX2
SLC34A2
ASIC2
GATA3
NANOG
CHSY3
YIPF3
TP63
KBTBD7
CEBPA
DOCK9
SPTLC2
ELF5
XAGE2
DAB2
c
Fibroblasts vs hbdTSCs
R^2= 0.01
164,166 peaks
51,948 peaks
61,777 peaks
Fib (KEN)
hbdTSCs
STK4
THY1
CDC14B
LATS2
TENM3
PELI2
TRPC4
POSTN
GJB3
BATF
GATA2
KCNK13
TP63
BMP7
GATA3
d
Fibroblasts vs hESCs
R^2= 0.09
152,413 peaks
42,768 peaks
54,234 peaks
Fib (KEN)
hESCs
LIPC
FNIP2
VPS53
RAI14
GAS1
CHPF2
IPO9
BECN1
NMNAT2
PDE10A
SOX2
TDGF1
NANOG
VRK1
e
GOKM D3 and OSKM D3 unique peaks overlapped with hbdTSC unique peaks
hbdTSCs
37565
4120
2010
568
OSKM D3
70019
57318
16230
GOKM D3
f
GOKM D3 and OSKM D3 unique peaks overlapped with hESC unique peaks
hESCs
27894
2174
1710
732
OSKM D3
71965
57618
16066
GOKM D3
g
hESC unique peaks when compared to fibroblasts (38,689)
GOKM D3 & hESCs shared peaks (3,211)
OSKM D3 & hESCs shared peaks (983)
GOKM D3
Fib (KEN)
OSKM D3
hbdTSCs
hESCs
A
B
Peaks
GOKM D3 and OSKM D3 differentially accessible peaks located within the same hbdTSC genes
h
R^2= 0.77
150,265 peaks
128 genes
4,536 peaks
13,371 peaks
OSKM D3
GOKM D3
GATA3
PITX1
KLF5
TEAD4
i
128 shared genes
OSKM D3
GOKM D3
AP1
p= 1e-20
GATA3
p= 1e-36
TEAD4
p= 1e-6
KLF5
p= 1e-12
SOX2
p= 1e-5
AP1
p= 1e-7
OCT4-SOX17
p= 1e-2
OCT4
p= 1e-7
j
Human Gene Atlas
Placenta
p= 7.028e-9
Lung
Bronchial cells
Liver
blood
GO Molecular Function
BMP receptor binding
p= 0.003
Epoxygenase activity
S/Th kinase binding
Cadherin binding
Iron ion binding
Oxidoreductase activity
Monooxygenase activity
Oxidoreductase activity
a-actin binding
Ferrous iron binding

Figure S3. ATAC-seq analysis on GOKM and OSKM-transduced cells following 3 days of transgene induction. (a) Venn diagram of ATAC-seq peaks for fibroblasts, hbdTSCs, and hESCs demonstrating the number of peaks that are unique (FDR< 0.05) to each cell type. **(b-d)** Scatter plots displaying pairwise correlations of differentially accessible peaks between hESCs vs hbdTSCs (b), fibroblasts vs hbdTSCs (c), and fibroblasts vs hESCs (d) (FDR< 0.05). Differential accessible peaks along the horizontal axes or the vertical axes are labeled by dark blue and dark orange, respectively. Peaks associated with genes that are expressed in the corresponding cell type along the horizontal axes are labeled with light blue, while peaks associated with genes that are specific to the vertical axes are labeled with light orange. The total number of exclusive peaks and representative genes for each cell type are depicted. **(e-f)** Venn diagrams of ATAC-seq peaks showing the overlap between 'GOKM D3' or 'OSKM D3'-unique peaks with hbdTSC-unique peaks (e) or hESC-unique peaks (f) (FDR< 0.05). **(g)** Heatmap showing the 3,211 GOKM and the 983 OSKM-unique peaks that overlap with the 38,689 hESC-unique peaks (after subtracting 'fibroblasts' peaks, FDR< 0.05). **(h)** Scatter plot displaying pairwise correlation of differentially accessible peaks between 'GOKM D3' and 'OSKM D3'. GOKM and OSKM exclusive peaks are labeled by dark blue and dark orange, respectively, while peaks that are associated with the same hbdTSC-expressed genes are marked by light blue (GOKM) and light orange (GOKM). The total number of exclusive peaks for each combination and the number of their shared associated genes (marked by green) are indicated. Representative genes and their corresponding peaks are depicted. **(i)** HOMER analysis on the peaks associated with the 128 shared hbdTSC genes from (g) reveals binding site enrichment for *GATA3*, *KLF5* and *OCT4* for GOKM and *SOX2*, *TEAD4* and *OCT4-SOX17* for OSKM. P-value was calculated using the binomial distributions. **(j)** Bar graphs showing the highest enriched GO terms and their p-value for the 128 shared hbdTSC genes from (g) using different categories within EnrichR. p-value was calculated using Fisher exact test. Source data are provided as a Source Data file.

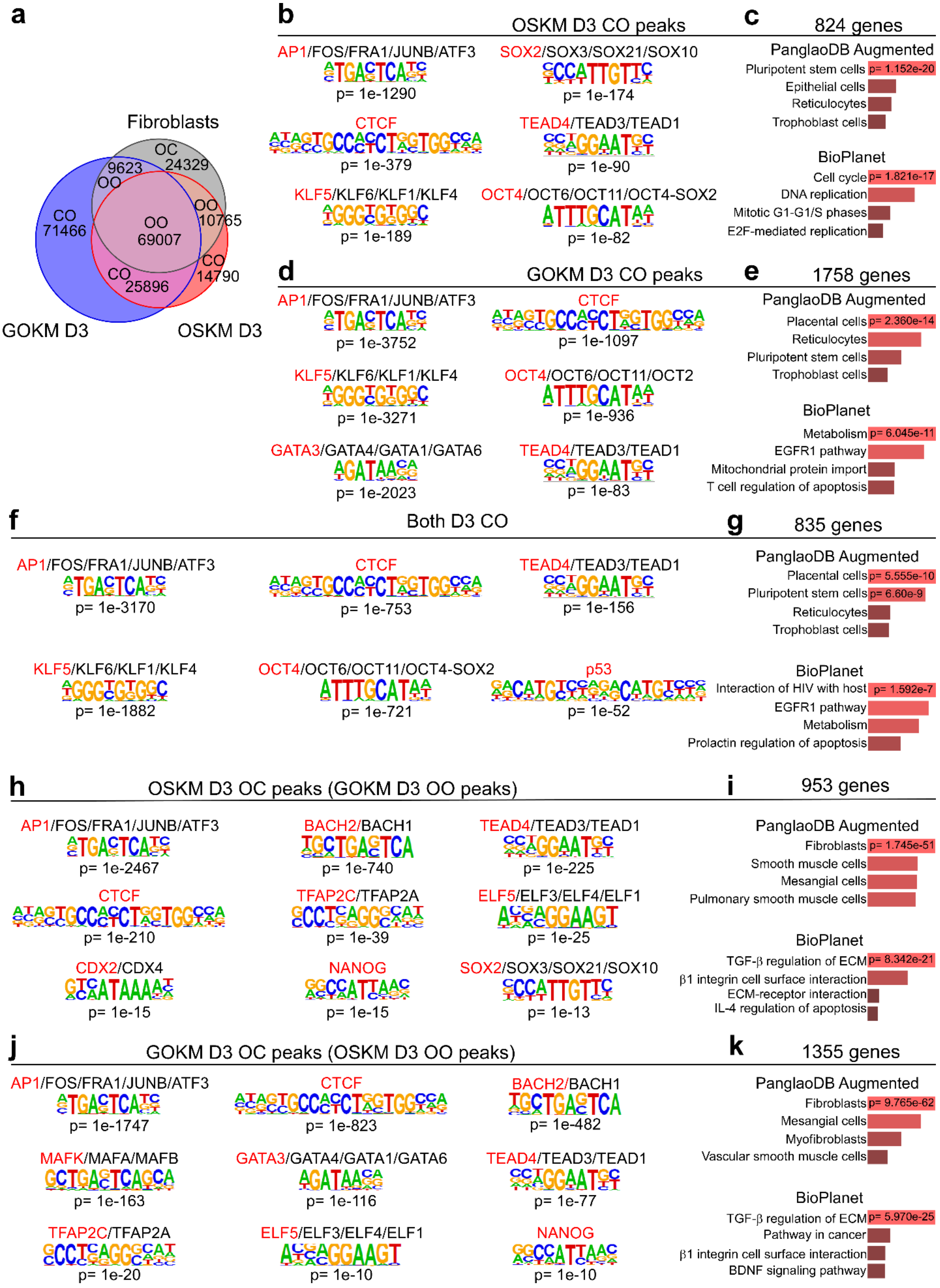
a
Fibroblasts
OC 24329
9623 OO
OO 10765
CO 71466
OO 69007
CO 25896
CO 14790
GOKM D3
OSKM D3
b
OSKM D3 CO peaks
AP1/FOS/FRA1/JUNB/ATF3
p= 1e-1290
SOX2/SOX3/SOX21/SOX10
p= 1e-174
CTCF
p= 1e-379
TEAD4/TEAD3/TEAD1
p= 1e-90
KLF5/KLF6/KLF1/KLF4
p= 1e-189
OCT4/OCT6/OCT11/OCT4-SOX2
p= 1e-82
c
824 genes
PanglaoDB Augmented
Pluripotent stem cells p= 1.152e-20
Epithelial cells
Reticulocytes
Trophoblast cells
BioPlanet
Cell cycle p= 1.821e-17
DNA replication
Mitotic G1-G1/S phases
E2F-mediated replication
d
GOKM D3 CO peaks
AP1/FOS/FRA1/JUNB/ATF3
p= 1e-3752
CTCF
p= 1e-1097
KLF5/KLF6/KLF1/KLF4
p= 1e-3271
OCT4/OCT6/OCT11/OCT2
p= 1e-936
GATA3/GATA4/GATA1/GATA6
p= 1e-2023
TEAD4/TEAD3/TEAD1
p= 1e-83
e
1758 genes
PanglaoDB Augmented
Placental cells p= 2.360e-14
Reticulocytes
Pluripotent stem cells
Trophoblast cells
BioPlanet
Metabolism p= 6.045e-11
EGFR1 pathway
Mitochondrial protein import
T cell regulation of apoptosis
f
Both D3 CO
AP1/FOS/FRA1/JUNB/ATF3
p= 1e-3170
CTCF
p= 1e-753
TEAD4/TEAD3/TEAD1
p= 1e-156
KLF5/KLF6/KLF1/KLF4
p= 1e-1882
OCT4/OCT6/OCT11/OCT4-SOX2
p= 1e-721
p53
p= 1e-52
g
835 genes
PanglaoDB Augmented
Placental cells p= 5.555e-10
Pluripotent stem cells p= 6.60e-9
Reticulocytes
Trophoblast cells
BioPlanet
Interaction of HIV with host p= 1.592e-7
EGFR1 pathway
Metabolism
Prolactin regulation of apoptosis
h
OSKM D3 OC peaks (GOKM D3 OO peaks)
AP1/FOS/FRA1/JUNB/ATF3
p= 1e-2467
BACH2/BACH1
p= 1e-740
TEAD4/TEAD3/TEAD1
p= 1e-225
CTCF
p= 1e-210
TFAP2C/TFAP2A
p= 1e-39
ELF5/ELF3/ELF4/ELF1
p= 1e-25
CDX2/CDX4
p= 1e-15
NANOG
p= 1e-15
SOX2/SOX3/SOX21/SOX10
p= 1e-13
i
953 genes
PanglaoDB Augmented
Fibroblasts p= 1.745e-51
Smooth muscle cells
Mesangial cells
Pulmonary smooth muscle cells
BioPlanet
TGF-β regulation of ECM p= 8.342e-21
β1 integrin cell surface interaction
ECM-receptor interaction
IL-4 regulation of apoptosis
j
GOKM D3 OC peaks (OSKM D3 OO peaks)
AP1/FOS/FRA1/JUNB/ATF3
p= 1e-1747
CTCF
p= 1e-823
BACH2/BACH1
p= 1e-482
MAFK/MAFA/MAFB
p= 1e-163
GATA3/GATA4/GATA1/GATA6
p= 1e-116
TEAD4/TEAD3/TEAD1
p= 1e-77
TFAP2C/TFAP2A
p= 1e-20
ELF5/ELF3/ELF4/ELF1
p= 1e-10
NANOG
p= 1e-10
k
1355 genes
PanglaoDB Augmented
Fibroblasts p= 9.765e-62
Mesangial cells
Myofibroblasts
Vascular smooth muscle cells
BioPlanet
TGF-β regulation of ECM p= 5.970e-25
Pathway in cancer
β1 integrin cell surface interaction
BDNF signaling pathway

Figure S4. GOKM and OSKM open and close different regions along the chromatin at early stages of reprogramming. (a) Venn diagram of ATAC-seq peaks for fibroblasts, 'GOKM D3', and 'OSKM D3' showing peaks that are unique (FDR< 0.05) to each group. Each set of peaks was defined as follows: closed in fibroblasts but open in induced cells (CO- closed open), open in fibroblasts but closed in induced cells (OC- open closed), open in fibroblasts and remains open in induced cells (OO- open open). Note that OO in GOKM is OC for OSKM, and vice versa. **(b-k)** Left- HOMER analysis on the corresponding peak set showing the most enriched motifs and their p-value. p-value was calculated using the binomial distributions. Right- Bar graphs showing the highest enriched GO terms and their p-value for each group of genes that is associated with its corresponding peak set. p-value was calculated using Fisher exact test. The number of genes that are associated with each peaks set is depicted. Enriched transcription factors motifs from the same family are depicted above each motif. Transcription factor binding motifs marked by red represent the shown motifs. Source data are provided as a Source Data file.

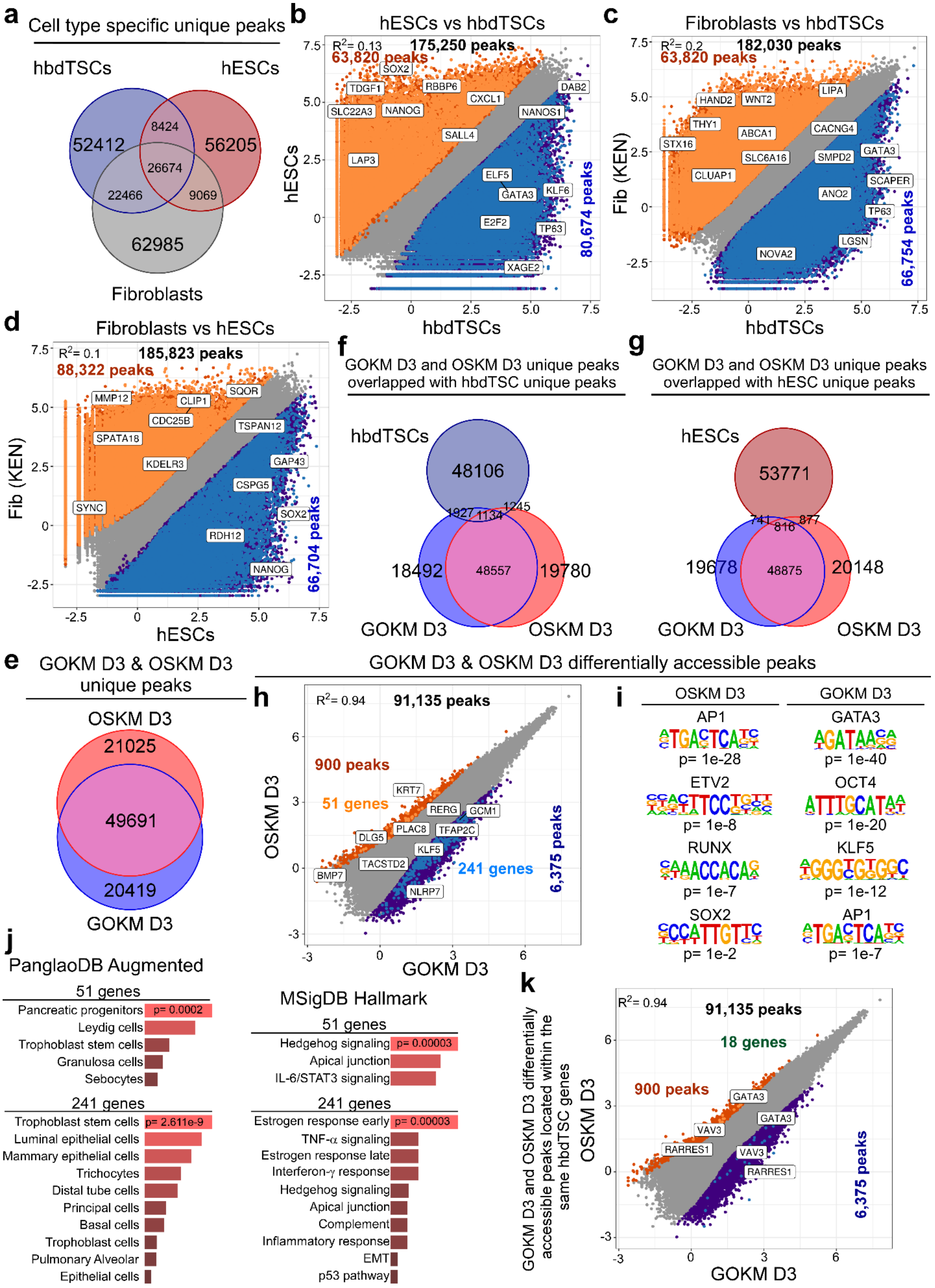
a
Cell type specific unique peaks
hbdTSCs
hESCs
Fibroblasts
52412
8424
56205
26674
22466
9069
62985
b
hESCs vs hbdTSCs
R^2= 0.13
175,250 peaks
63,820 peaks
80,674 peaks
SOX2
TDGF1
RBBP6
CXCL1
DAB2
SLC22A3
NANOG
NANOS1
SALL4
LAP3
ELF5
GATA3
KLF6
E2F2
TP63
XAGE2
hESCs
hbdTSCs
c
Fibroblasts vs hbdTSCs
R^2= 0.2
182,030 peaks
63,820 peaks
66,754 peaks
HAND2
WNT2
LIPA
THY1
ABCA1
CACNG4
STX16
SLC6A16
SMPD2
GATA3
CLUAP1
SCAPER
ANO2
TP63
LGSN
NOVA2
Fib (KEN)
hbdTSCs
d
Fibroblasts vs hESCs
R^2= 0.1
185,823 peaks
88,322 peaks
66,704 peaks
MMP12
CLIP1
SQOR
CDC25B
TSPAN12
SPATA18
KDELR3
GAP43
CSPG5
SYNC
SOX2
RDH12
NANOG
Fib (KEN)
hESCs
e
GOKM D3 & OSKM D3 unique peaks
OSKM D3
21025
49691
20419
GOKM D3
f
GOKM D3 and OSKM D3 unique peaks overlapped with hbdTSC unique peaks
hbdTSCs
48106
1927
1134
1245
18492
48557
19780
GOKM D3
OSKM D3
g
GOKM D3 and OSKM D3 unique peaks overlapped with hESC unique peaks
hESCs
53771
741
816
877
19678
48875
20148
GOKM D3
OSKM D3
GOKM D3 & OSKM D3 differentially accessible peaks
h
R^2= 0.94
91,135 peaks
900 peaks
51 genes
241 genes
6,375 peaks
KRT7
RERG
GCM1
PLAC8
TFAP2C
DLG5
KLF5
TACSTD2
BMP7
NLRP7
OSKM D3
GOKM D3
i
OSKM D3
GOKM D3
AP1
p= 1e-28
GATA3
p= 1e-40
ETV2
p= 1e-8
OCT4
p= 1e-20
RUNX
p= 1e-7
KLF5
p= 1e-12
SOX2
p= 1e-2
AP1
p= 1e-7
j
PanglaoDB Augmented
51 genes
Pancreatic progenitors p= 0.0002
Leydig cells
Trophoblast stem cells
Granulosa cells
Sebocytes
241 genes
Trophoblast stem cells p= 2.611e-9
Luminal epithelial cells
Mammary epithelial cells
Trichocytes
Distal tube cells
Principal cells
Basal cells
Trophoblast cells
Pulmonary Alveolar
Epithelial cells
MSigDB Hallmark
51 genes
Hedgehog signaling p= 0.00003
Apical junction
IL-6/STAT3 signaling
241 genes
Estrogen response early p= 0.00003
TNF-α signaling
Estrogen response late
Interferon-γ response
Hedgehog signaling
Apical junction
Complement
Inflammatory response
EMT
p53 pathway
k
GOKM D3 and OSKM D3 differentially accessible peaks located within the same hbdTSC genes
R^2= 0.94
91,135 peaks
18 genes
900 peaks
6,375 peaks
GATA3
VAV3
RARRES1
OSKM D3
GOKM D3

Figure S5. ChIP-seq analysis for H3K4me2 on GOKM and OSKM-transduced cells. (a) Venn diagram of ChIP-seq for H3K4me2 showing peaks that are unique to 'fibroblasts', 'hbdTSCs', and 'hESCs' (FDR< 0.05). **(b-d)** Scatter plots displaying pairwise correlations of differentially deposited peaks between 'hESCs' vs 'hbdTSCs' (b), 'fibroblasts' vs 'hbdTSCs' (c), and 'fibroblasts' vs 'hESCs' (d) FDR< 0.05). Differential deposited peaks along the horizontal axes or the vertical axes are labeled by dark blue and dark orange, respectively. Peaks associated with genes that are expressed in the corresponding cell type along the horizontal axes are labeled with light blue. Peaks associated with genes that are specific to samples along the vertical axes are labeled with light orange. The total number of exclusive peaks and representative genes for each cell type are depicted. **(e)** Venn diagram of 'GOKM D3' and 'OSKM D3'-unique H3K4me2 peaks after subtracting 'fibroblasts' peaks (FDR < 0.05). **(f-g)** Venn diagrams of H3K4me2 peaks showing the overlap between 'GOKM D3' or 'OSKM D3'-unique peaks with 'hbdTSC'-unique peaks (f) or 'hESC'-unique peaks (g) (FDR< 0.05). **(h)** Scatter plot of differentially accessible peaks between 'GOKM D3' and 'OSKM D3' (FDR< 0.05). Peaks that are exclusive to GOKM or OSKM are labeled with dark blue and dark orange, respectively. Peaks associated with 'hbdTSC'-expressed genes are labeled with light blue (GOKM) and light orange (OSKM). The total number of exclusive peaks, the number of their associated genes and representative genes are depicted. **(i)** HOMER analysis on OSKM or GOKM exclusive peaks reveals motif enrichment for each combination. P-value was calculated using the binomial distributions. **(j)** Bar graphs showing the most enriched GO terms, and their p-value, for the 241 or 51 genes from (h) using EnrichR. P-value was calculated using Fisher exact test. **(k)** Scatter plot displaying pairwise correlations of differentially deposited peaks between 'GOKM D3' and 'OSKM D3'. GOKM/OSKM exclusive peaks are labeled by dark blue and dark orange, respectively. Peaks that are associated with the same hbdTSC-expressed genes are marked by light blue (GOKM) and light orange (GOKM). Source data are provided as a Source Data file.

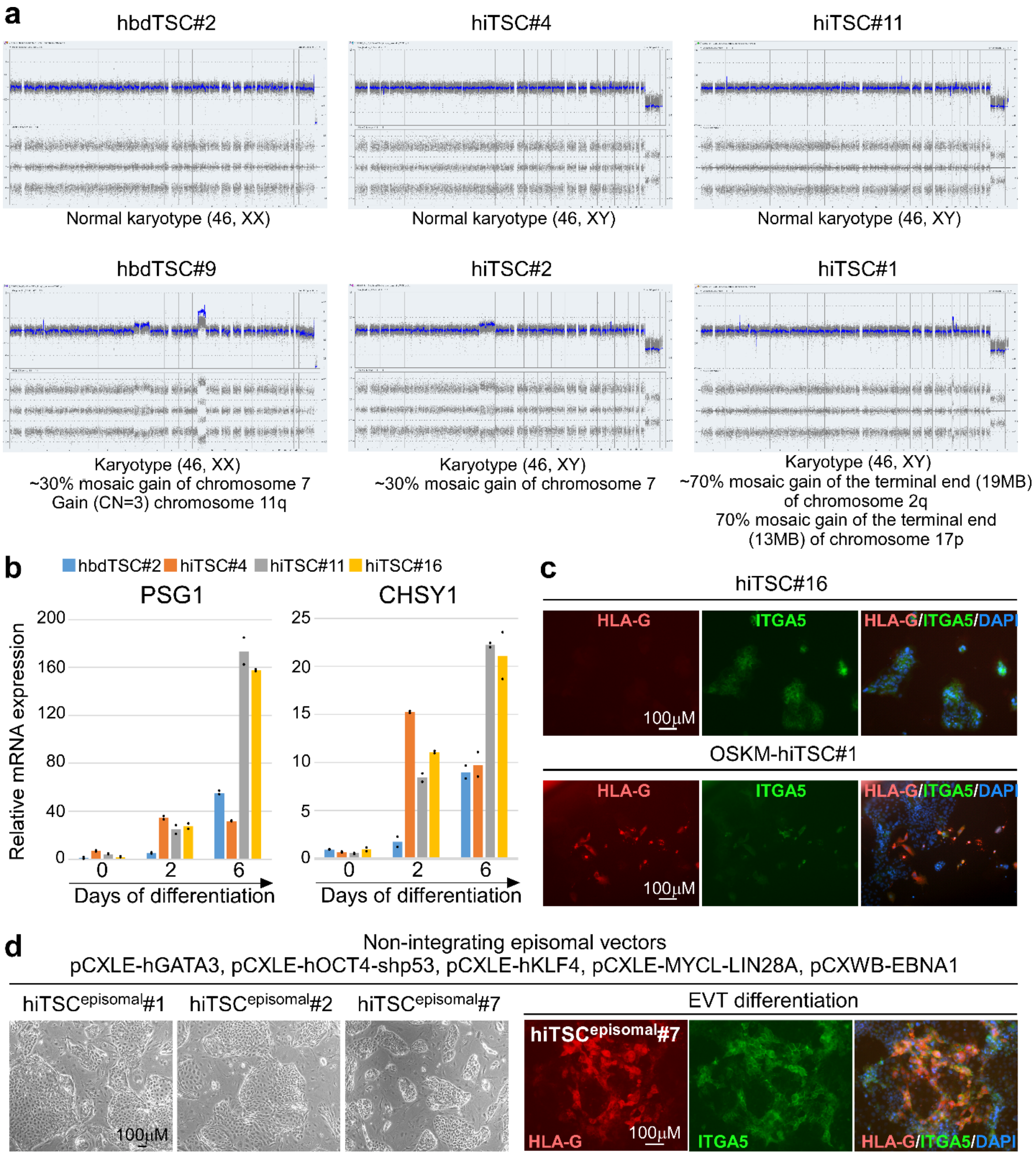
a
hbdTSC#2
hiTSC#4
hiTSC#11
Normal karyotype (46, XX)
Normal karyotype (46, XY)
Normal karyotype (46, XY)
hbdTSC#9
hiTSC#2
hiTSC#1
Karyotype (46, XX)
~30% mosaic gain of chromosome 7
Gain (CN=3) chromosome 11q
Karyotype (46, XY)
~30% mosaic gain of chromosome 7
Karyotype (46, XY)
~70% mosaic gain of the terminal end (19MB) of chromosome 2q
70% mosaic gain of the terminal end (13MB) of chromosome 17p
b
hbdTSC#2 hiTSC#4 hiTSC#11 hiTSC#16
PSG1
CHSY1
Relative mRNA expression
200
160
120
80
40
0
25
20
15
10
5
0
0 2 6
Days of differentiation
c
hiTSC#16
HLA-G
ITGA5
HLA-G/ITGA5/DAPI
100μM
OSKM-hiTSC#1
d
Non-integrating episomal vectors
pCXLE-hGATA3, pCXLE-hOCT4-shp53, pCXLE-hKLF4, pCXLE-MYCL-LIN28A, pCXWB-EBNA1
hiTSCepisomal#1
hiTSCepisomal#2
hiTSCepisomal#7
EVT differentiation

Figure S6. hiTSCs can maintain normal karyotype and differentiate into STs and EVTs. Both hbdTSC#2 and hbdTSC#9 were isolated from PGD embryos (see Methods). Geneic examination of the two lines reveals that hbdTSC#2 is heterozygous for RB mutation and hbdTSC#9 is heterozygous for RB and Marfan mutations **(a)** Plots displaying the karyotype of hbdTSC and hiTSC lines. Two hbdTSC lines, hbdTSC#2 and hbdTSC#9, and four hiTSC clones (n=4), hiTSC#1, hiTSC#2, hiTSC#4 and hiTSC#11, were subjected to karyotyping analysis using Affymetrix CytoScan 750K array. 50% of hbdTSC lines and 50% of hiTSC lines displayed an intact chromosomal karyotype. The other 50% of the colonies exhibited few aberrations in a fraction of the cells. The specific aberrations and the relevant affected fraction of the cells are specified below each plot. **(b)** qPCR analysis of relative mRNA levels of ST marker genes *PSG1* and *CHSY1* at days 0, 2 and 6 in ST differentiation protocol. Results were normalized to the mRNA levels of the housekeeping control gene *GAPDH* and are shown as fold change relative to day 0 control cells (hbdTSCs). Error bars indicate standard deviation between two duplicates. **(c)** Immunofluorescence staining for the EVT-specific markers HLA-G and ITGA5 and DAPI nuclear staining in PFA-fixated hiTSC#16 and OSKM-hiTSC#1 following 14 days of EVT differentiation. **(d, left)** Bright field images of isolated hiTSC clones derived using the indicated episomes, delivered through electroporation. **(d, right)** Immunofluorescence staining for the EVT-specific markers HLA-G and ITGA5 and DAPI nuclear staining in PFA-fixated hiTSC^{episomal}#7 following 14 days of EVT differentiation. Source data are provided as a Source Data file.

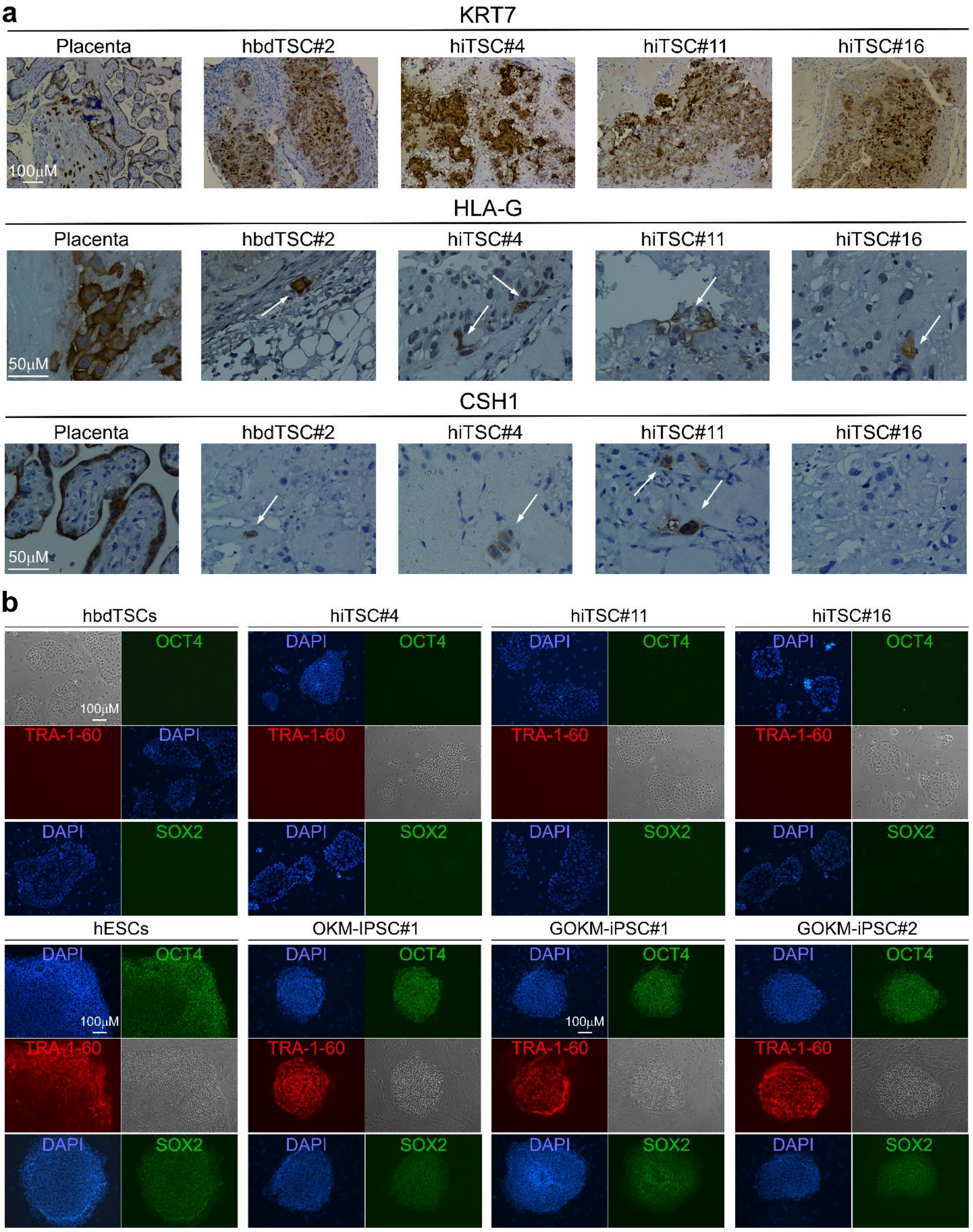

a
KRT7
Placenta
hbdTSC#2
hiTSC#4
hiTSC#11
hiTSC#16
100μM
HLA-G
Placenta
hbdTSC#2
hiTSC#4
hiTSC#11
hiTSC#16
50μM
CSH1
Placenta
hbdTSC#2
hiTSC#4
hiTSC#11
hiTSC#16
50μM
b
hbdTSCs
hiTSC#4
hiTSC#11
hiTSC#16
DAPI
OCT4
TRA-1-60
SOX2
100μM
hESCs
OKM-IPSC#1
GOKM-iPSC#1
GOKM-iPSC#2

Figure S7. hiTSCs engrafted into NOD-SCID mice form trophoblastic lesions and hiTSCs are negative to pluripotency-specific markers. For lesion formation approximately $4x10^6$ hbdTSCs or hiTSCs were subcutaneously injected into NOD-SCID mice. Lesions were collected nine days after injection and analyzed by immunohistochemistry for specific markers. **(a)** Immunohistochemically stained sections of human placenta and trophoblastic lesions extracted from SCID-NOD mice showing strong KRT7 staining (top) and scattered staining for the EVT marker HLA-G (middle) and ST marker CSH1 (bottom). White arrows point to positive staining for the indicated markers. Three independent lesions were stained for each clone (n=3) **(b)** hiTSCs are negative to pluripotent-specific markers. Fluorescent images displaying high expression levels of OCT4, SOX2 and TRA-1-60 in pluripotent stem cells (hESCs, GOKM-hiPSCs and OKM-hiPSCs, bottom panel) and none (OCT4 and TRA-1-60) to very low expression (SOX2) in 3 independent hiTSC clones (n=3, upper panel).

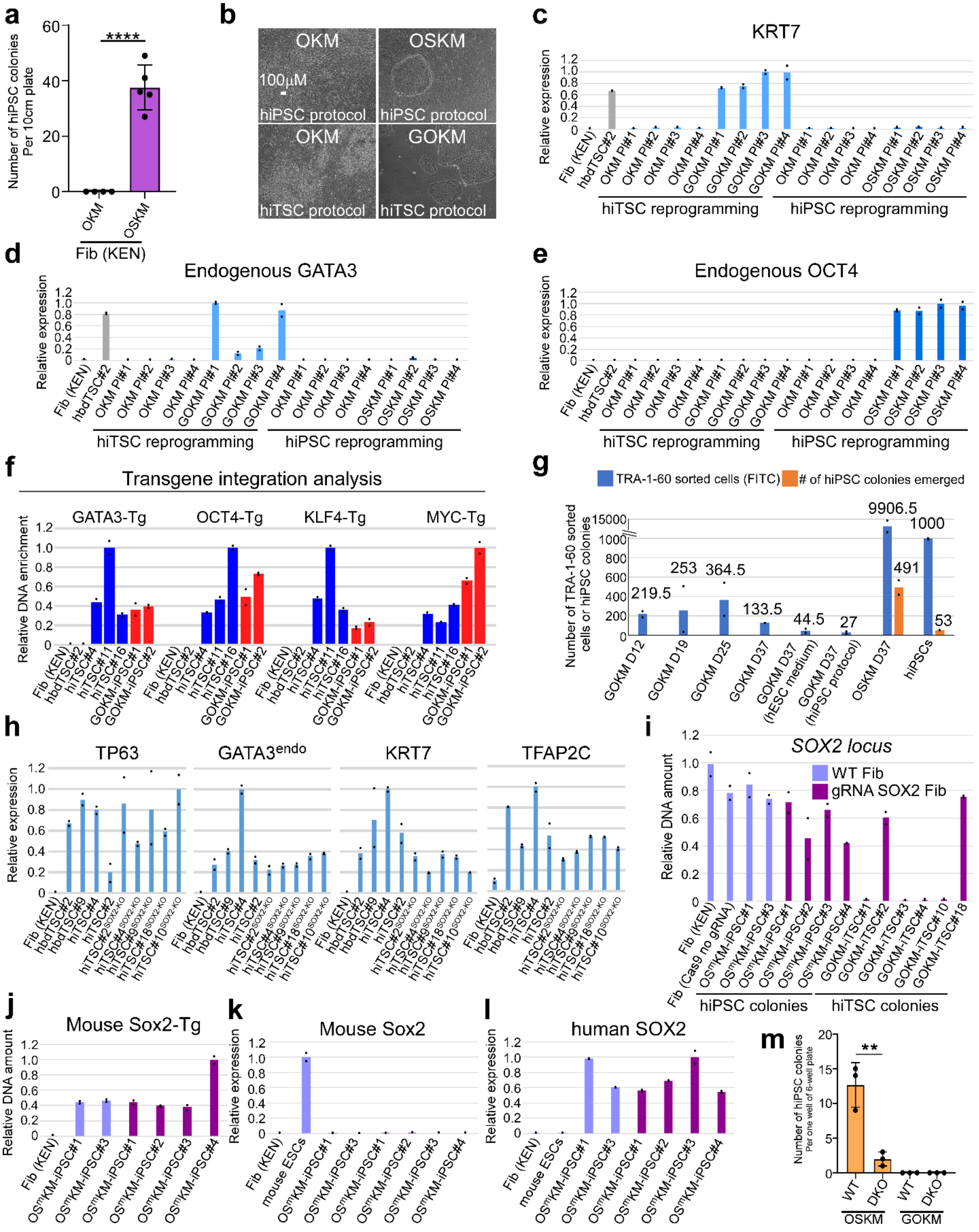
a
Number of hiPSC colonies Per 10cm plate
****
OKM
OSKM
Fib (KEN)
b
OKM
OSKM
100μM
hiPSC protocol
hiPSC protocol
OKM
GOKM
hiTSC protocol
hiTSC protocol
c
KRT7
Relative expression
Fib (KEN)
hbdTSC#2
OKM PI#1
OKM PI#2
OKM PI#3
OKM PI#4
GOKM PI#1
GOKM PI#2
GOKM PI#3
GOKM PI#4
OKM PI#1
OKM PI#2
OKM PI#3
OKM PI#4
OSKM PI#1
OSKM PI#2
OSKM PI#3
OSKM PI#4
hiTSC reprogramming
hiPSC reprogramming
d
Endogenous GATA3
Relative expression
hiTSC reprogramming
hiPSC reprogramming
e
Endogenous OCT4
Relative expression
hiTSC reprogramming
hiPSC reprogramming
f
Transgene integration analysis
GATA3-Tg
OCT4-Tg
KLF4-Tg
MYC-Tg
Relative DNA enrichment
Fib (KEN)
hbdTSC#2
hiTSC#4
hiTSC#11
hiTSC#16
GOKM-iPSC#1
GOKM-iPSC#2
g
TRA-1-60 sorted cells (FITC)
# of hiPSC colonies emerged
Number of TRA-1-60 sorted cells or hiPSC colonies
15000
1000
800
600
400
200
0
219.5
253
364.5
133.5
44.5
27
9906.5
491
1000
53
GOKM D12
GOKM D19
GOKM D25
GOKM D37
GOKM D37 (hESC medium)
GOKM D37 (hiPSC protocol)
OSKM D37
hiPSCs
h
TP63
GATA3endo
KRT7
TFAP2C
Relative expression
Fib (KEN)
hbdTSC#2
hbdTSC#9
hiTSC#4
hiTSC#2
hiTSC#2SOX2-KO
hiTSC#4SOX2-KO
hiTSC#9SOX2-KO
hiTSC#18SOX2-KO
hiTSC#10SOX2-KO
i
SOX2 locus
Relative DNA amount
WT Fib
gRNA SOX2 Fib
Fib (KEN)
Fib (Cas9 no gRNA)
OSmKM-iPSC#1
OSmKM-iPSC#3
OSmKM-iPSC#1
OSmKM-iPSC#2
OSmKM-iPSC#3
OSmKM-iPSC#4
GOKM-iTSC#1
GOKM-iTSC#2
GOKM-iTSC#3
GOKM-iTSC#4
GOKM-iTSC#10
GOKM-iTSC#18
hiPSC colonies
hiTSC colonies
j
Mouse Sox2-Tg
Relative DNA amount
Fib (KEN)
OSmKM-iPSC#1
OSmKM-iPSC#3
OSmKM-iPSC#1
OSmKM-iPSC#2
OSmKM-iPSC#3
OSmKM-iPSC#4
k
Mouse Sox2
Relative expression
Fib (KEN)
mouse ESCs
OSmKM-iPSC#1
OSmKM-iPSC#3
OSmKM-iPSC#1
OSmKM-iPSC#2
OSmKM-iPSC#3
OSmKM-iPSC#4
l
human SOX2
Relative expression
Fib (KEN)
mouse ESCs
OSmKM-iPSC#1
OSmKM-iPSC#3
OSmKM-iPSC#1
OSmKM-iPSC#2
OSmKM-iPSC#3
OSmKM-iPSC#4
m
Number of hiPSC colonies Per one well of 6-well plate
**
WT
DKO
WT
DKO
OSKM
GOKM

Figure S8. GOKM do not acquire pluripotency during hiTSC formation. (a) Graph showing an average number of hiPSC colonies generated by either OKM or OSKM. Error bars indicate standard deviation between independent experiments/replicates (OKM n=4, OSKM n=5). **** indicates p-value< 0.0001 (95% confidence interval 27.87 to 47.33), using 2-tailed unpaired t test calculated by GraphPad Prism (8.3.0). Mean values (from left to right) are: 0.000, 37.60. **(b)** Bright field images of plates which were reprogrammed with OKM or OSKM using hiPSC (top) or hiTSC (bottom) reprogramming protocol. **(c-e)** qPCR analysis of mRNA levels of *KRT7* (c)*,* endogenous *GATA3* (d) and endogenous *OCT4* (e) in plates (4=4) transduced with OKM, GOKM or OSKM following hiPSC or hiTSC reprogramming protocols. Fibroblasts and hbdTSCs were used as controls. The highest sample for each gene was set to 1. Results were normalized to the mRNA levels of *GAPDH* and are shown as fold change. Two replicates were used from each sample (n= 2). **(f)** qPCR analysis for the relative DNA enrichment of GOKM transgene integration in the indicated samples. The highest sample for each transgene was set to 1. Results were normalized to *GAPDH* genomic regions and are shown as fold change. Two replicates (n= 2) were used for each sample. **(g)** Graph displaying the average number of TRA-1-60-positive cells that were sorted during GOKM-mediated hiTSC or hiPSC reprogramming protocols at the indicated time points, and the number of hiPSC colonies that emerged in each plate. Two biological replicates (n= 2) were used for each condition. **(h)** qPCR analysis of mRNA levels of the indicated genes and samples. The highest sample for each gene was set to 1. Results were normalized to *GAPDH* and are shown as fold change. Two replicates (n= 2) were used for each sample. **(I)** qPCR analysis for the relative DNA enrichment of the human *SOX2* genomic locus in six OKM+ mouse SOX2 ($OS^{m}KM$)-derived hiPSC colonies and six GOKM-derived hiTSC colonies in either WT or "*SOX2* KO" fibroblasts. The highest sample for each gene was set to 1. Results were normalized to genomic region of *GAPDH* and are shown as fold change. Two replicates (n= 2) were used for each sample. **(j-l)** qPCR analysis for the relative DNA enrichment of the mouse *Sox2 transgene* (J), mRNA levels of mouse *Sox2* gene (k) and mRNA levels of human *SOX2* gene (l) in the indicated samples. The highest sample for each gene was set to 1. Results were normalized to *GAPDH* and are shown as fold change. Two replicates (n= 2) were used for each sample **(m)** Graph displaying the number of hiPSC colonies that were generated by OSKM or GOKM factors in WT or double knockout (DKO, for *NANOG* and *PRDM14*) fibroblasts. Error bars indicate standard deviation between 3 independent experiments/replicates (n=3). ** indicates p-value of 0.0054 (95% confidence interval -16.06 to -5.270), using 2-tailed unpaired t test calculated by GraphPad Prism (8.3.0). Mean values (from left to right) are: 12.67, 2.000, 0.000, 0.000. Source data are provided as a Source Data file.

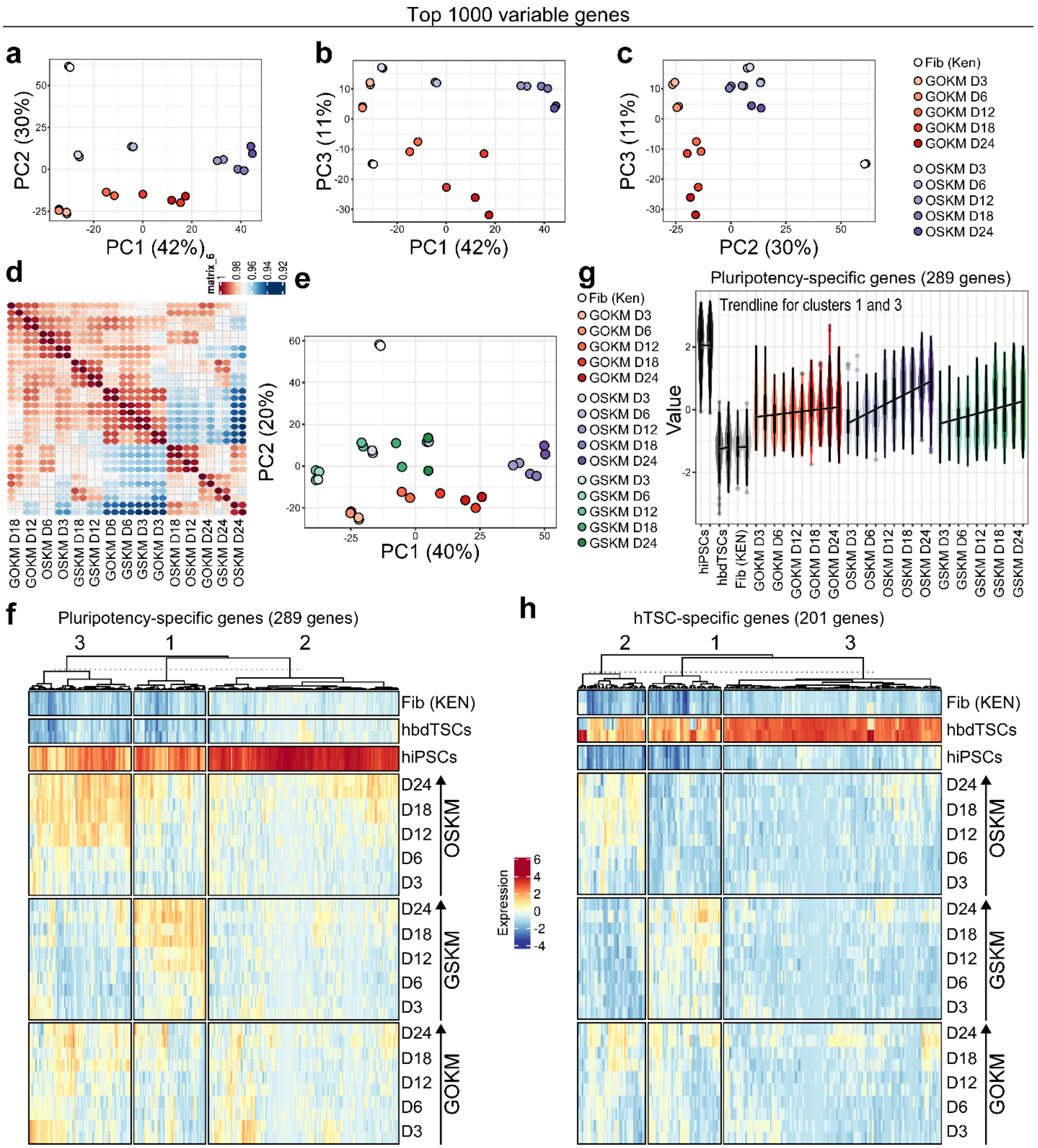

Top 1000 variable genes
a
b
c
d
e
f
g
h
PC1 (42%)
PC2 (30%)
PC3 (11%)
PC1 (40%)
PC2 (20%)
Fib (Ken)
GOKM D3
GOKM D6
GOKM D12
GOKM D18
GOKM D24
OSKM D3
OSKM D6
OSKM D12
OSKM D18
OSKM D24
GSKM D3
GSKM D6
GSKM D12
GSKM D18
GSKM D24
Pluripotency-specific genes (289 genes)
Trendline for clusters 1 and 3
Value
hiPSCs
hbdTSCs
Fib (KEN)
hTSC-specific genes (201 genes)
Expression
OSKM
GSKM
GOKM
D24
D18
D12
D6
D3

Figure S9. GOKM do not activate OSKM nor pluripotency gene signatures. (a-c) Principal component analysis for top 1000 variable genes among all samples showing no overlap in gene expression between OSKM and GOKM reprogramming samples (a, PC1 vs PC2; b, PC1 vs PC3; c, PC2 vs PC3). **(d)** Correlation heatmap based on RNA-seq data portraying comparisons of whole transcriptome of two biological duplicates of the indicated samples. **(e)** Principal component analysis for top 1000 variable genes among all samples showing clear overlap between OSKM and GSKM reprogramming samples. **(f)** Heatmap and clustering tree for 289 unique pluripotency genes along the reprogramming process (day 3, 6, 12, 18 and 24) of OSKM, GSKM and GOKM**.** Pluripotency gene signature was defined by selecting genes that are uniquely expressed (logFC>6) in pluripotent cells when compared to hbdTSCs and fibroblasts. **(g)** Violin plots showing the average expression of the 289 pluripotency genes (n= 289) in the indicated samples. Two biological replicates (n=2) are used for each sample or condition. The center line denotes the median value (50th percentile), and box limits contain the dataset's 25th to 75th percentiles. The black whiskers mark the 5th and 95th percentiles. Trendline (black) for clusters 1 and 3 is depicted for each reprogramming combination. **(h)** Heatmap and clustering tree for 201 unique hTSC genes along the reprogramming process (day 3, 6, 12, 18 and 24) of OSKM, GSKM and GOKM**.** hTSC gene signature was defined by selecting genes that are uniquely expressed (logFC>6) in hbdTSCs when compared to hiPSCs and fibroblasts.

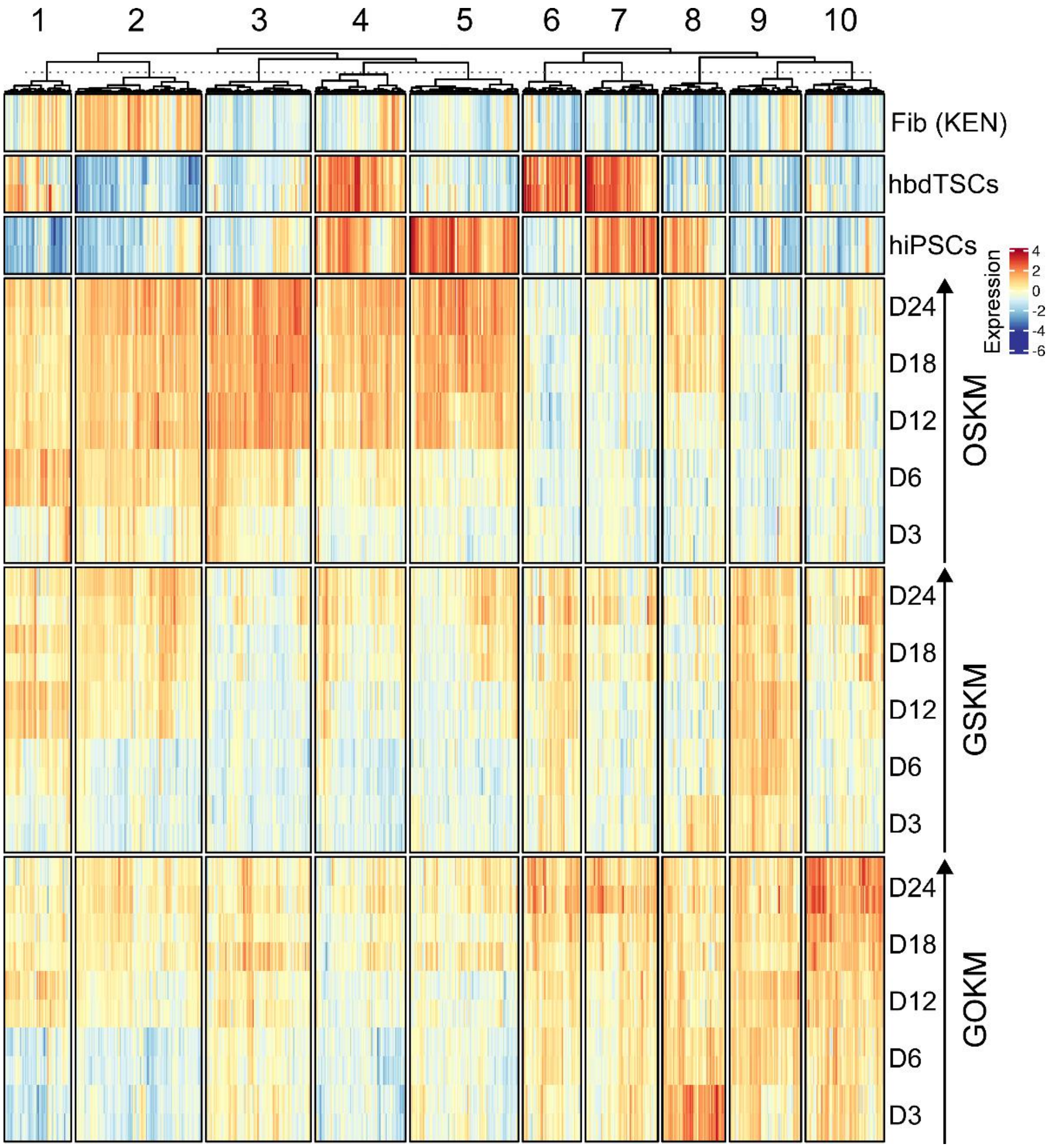

1
2
3
4
5
6
7
8
9
10
Fib (KEN)
hbdTSCs
hiPSCs
Expression
4
2
0
-2
-4
-6
D24
D18
D12
D6
D3
OSKM
D24
D18
D12
D6
D3
GSKM
D24
D18
D12
D6
D3
GOKM

Figure S10. GATA3 and OCT4 activate a unique set of genes to achieve the hTSC state. Clustering analysis of genes that showed differential expression (LogFC>3) between OSKM and GOKM in at least one-time point during the reprogramming process. Gap statistics analysis on the identified 706 differentially expressed genes resulted in 10 defined clusters with unique specificity to cell types and transcription factors.

New Composite 2 - Plot Sheet 1

Data Set 1: KEN fibroblasts p12 no primary antibody ? Event Count: 15,028

Data Set 1: KEN fibroblasts p12 no primary antibody
[Ungated] FS Area / SS Area

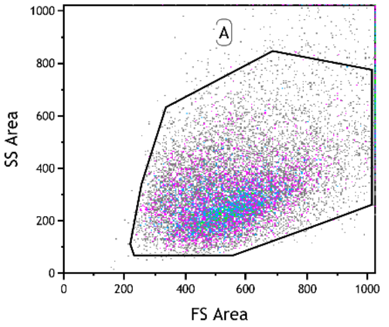

Gate	Number
All	15,028
A	13,933

Data Set 1: KEN fibroblasts p12 no primary antibody
[A] SS Area / SS Height

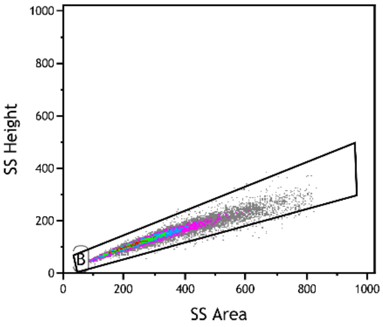

Data Set 1: KEN fibroblasts p12 no primary antibody
[B] FL1 Area

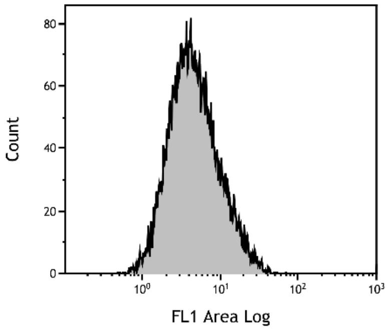

Gate	X-GMean
All	4.62

Data Set 2: KEN fibroblasts p12 W6/32 antibody
[Ungated] FS Area / SS Area

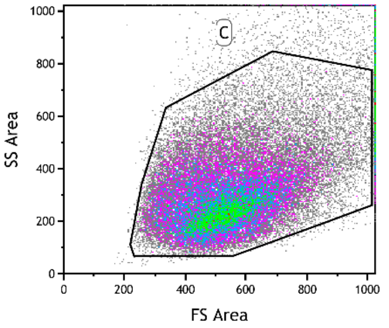

Gate	Number
All	51,789
C	48,100

Data Set 2: KEN fibroblasts p12 W6/32 antibody
[C] SS Area / SS Height

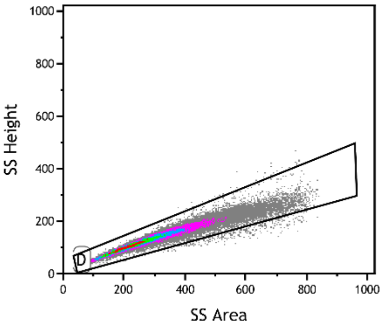

Data Set 2: KEN fibroblasts p12 W6/32 antibody
[D] FL1 Area

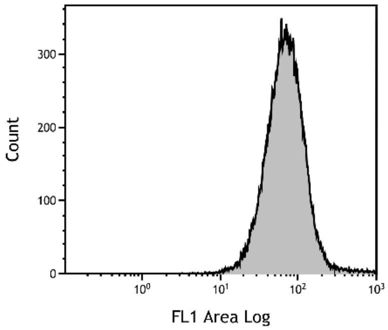

Gate	X-GMean
All	69.75

Data Set 3: PCS fibroblasts p8 W6/32 antibody
[Ungated] FS Area / SS Area

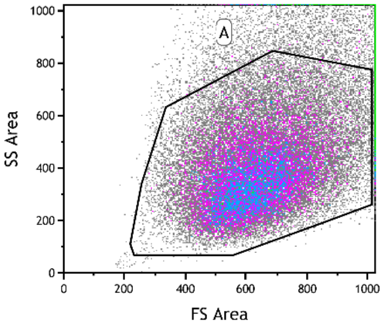

Gate	Number
All	50,663
A	40,266

Data Set 3: PCS fibroblasts p8 W6/32 antibody
[A] SS Area / SS Height

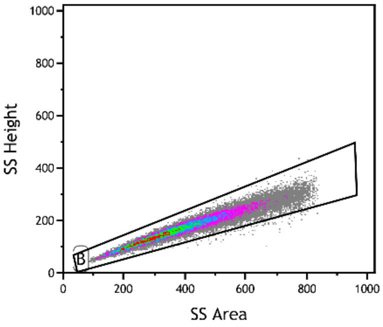

Data Set 3: PCS fibroblasts p8 W6/32 antibody
[B] FL1 Area

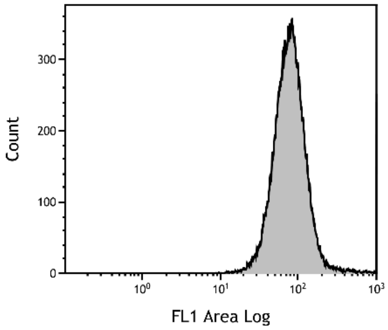

Gate	X-GMean
All	79.31

New Composite 2 - Plot Sheet 1

Data Set 4: GM2 fibroblasts p7 W6/32 antibody ? Event Count: 50,212

Data Set 4: GM2 fibroblasts p7 W6/32 antibody
[Ungated] FS Area / SS Area

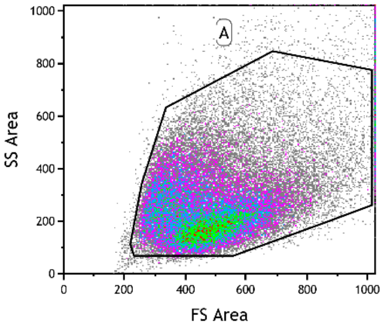

Gate	Number
All	50,212
A	47,911

Data Set 4: GM2 fibroblasts p7 W6/32 antibody
[A] SS Area / SS Height

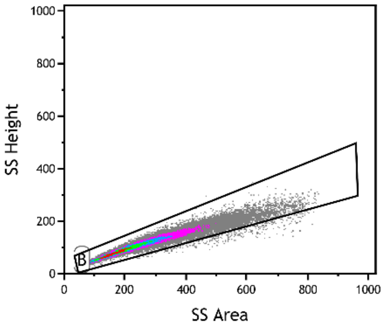

Data Set 4: GM2 fibroblasts p7 W6/32 antibody
[B] FL1 Area

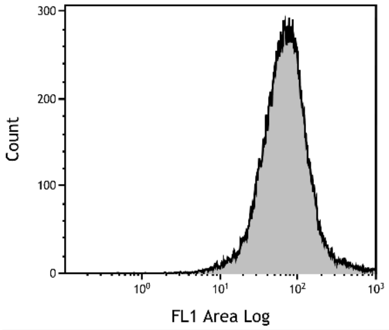

Gate	X-GMean
All	70.50

Data Set 6: bdTSC#2 p13 W6/32 antibody
[Ungated] FS Area / SS Area

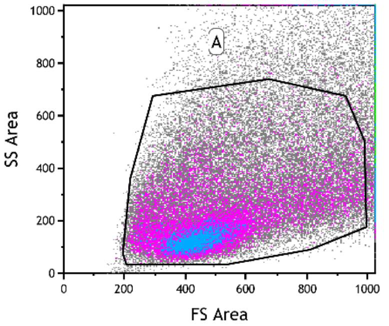

Gate	Number
All	103,966
A	51,002

Data Set 6: bdTSC#2 p13 W6/32 antibody
[A] SS Area / SS Height

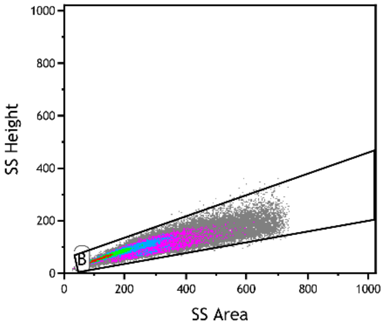

Data Set 6: bdTSC#2 p13 W6/32 antibody
[B] FL1 Area

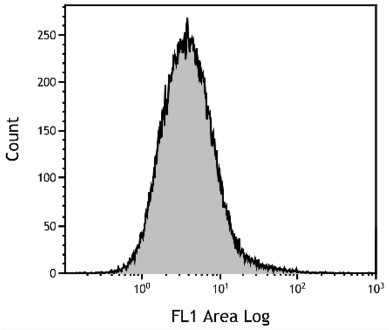

Gate	X-GMean
All	3.98

Data Set 7: hiTSC#4 p11 W6/32 antibody ? Event Count: 136,722

Data Set 7: hiTSC#4 p11 W6/32 antibody
[Ungated] FS Area / SS Area

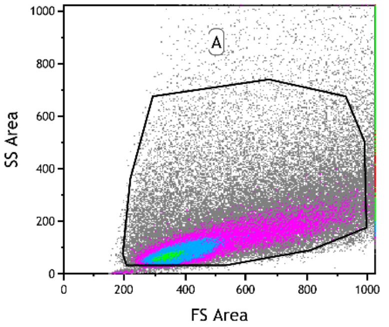

Gate	Number
All	136,722
A	88,179

Data Set 7: hiTSC#4 p11 W6/32 antibody
[A] SS Area / SS Height

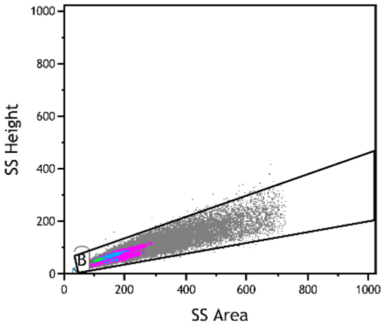

Data Set 7: hiTSC#4 p11 W6/32 antibody
[B] FL1 Area

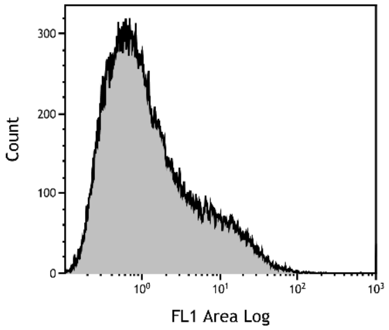

Gate	X-GMean
All	1.26

New Composite 2 - Plot Sheet 1

Data Set 8: hiTSC#11 p13 W6/32 antibody ? Event Count: 108,560

Data Set 8: hiTSC#11 p13 W6/32 antibody
[Ungated] FS Area / SS Area

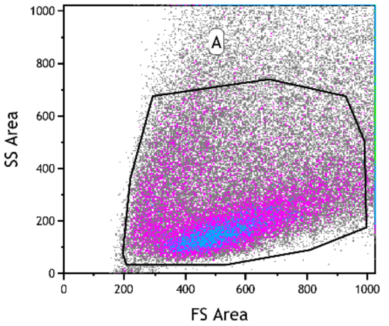

Gate	Number
All	108,560
A	49,612

Data Set 8: hiTSC#11 p13 W6/32 antibody
[A] SS Area / SS Height

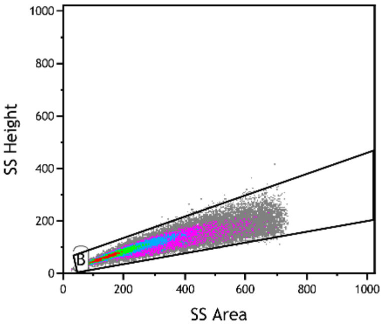

Data Set 8: hiTSC#11 p13 W6/32 antibody
[B] FL1 Area

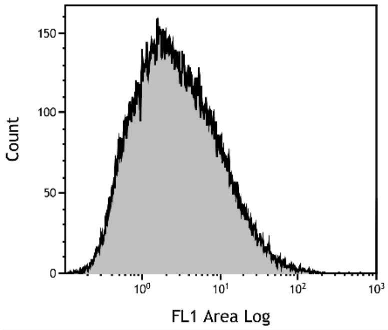

Gate	X-GMean
All	2.74

Data Set 9: hiTSC#16 p12 W6/32 antibody ? Event Count: 130,232

Data Set 9: hiTSC#16 p12 W6/32 antibody
[Ungated] FS Area / SS Area

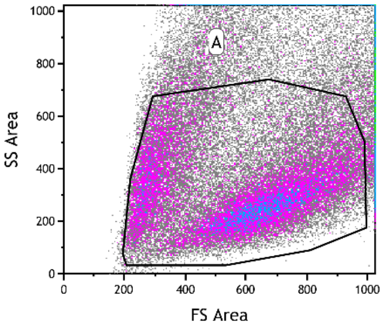

Gate	Number
All	130,232
A	49,892

Data Set 9: hiTSC#16 p12 W6/32 antibody
[A] SS Area / SS Height

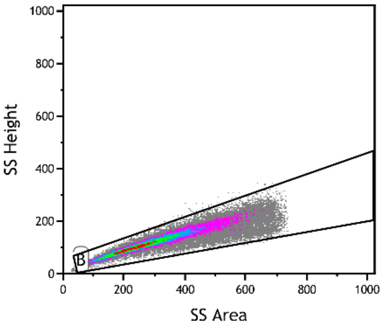

Data Set 9: hiTSC#16 p12 W6/32 antibody
[B] FL1 Area

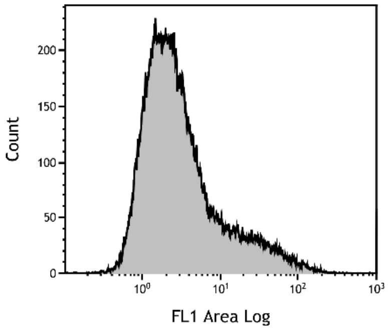

Gate	X-GMean
All	3.20

BD FACSDiva 8.0.1

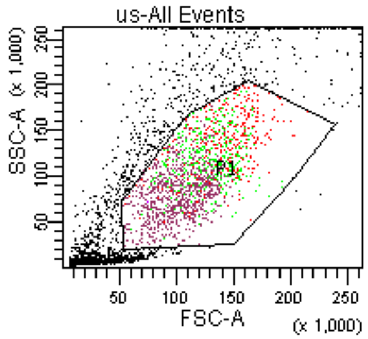

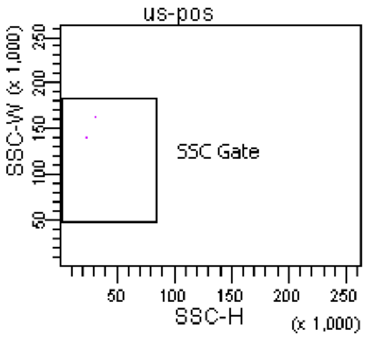

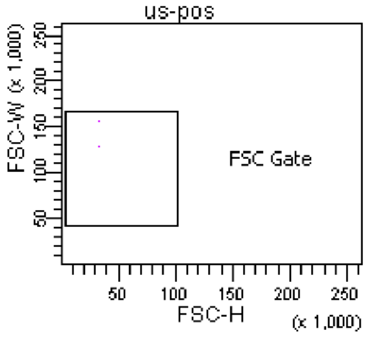

Tube: us

Population	#Events	%Parent	%Total
All Events	10,000	####	100.0
P1	4,571	45.7	45.7
SSC Gate	3,386	74.1	33.9
FSC Gate	2,284	67.5	22.8
pos	2	0.1	0.0
neg	2,282	99.9	22.8

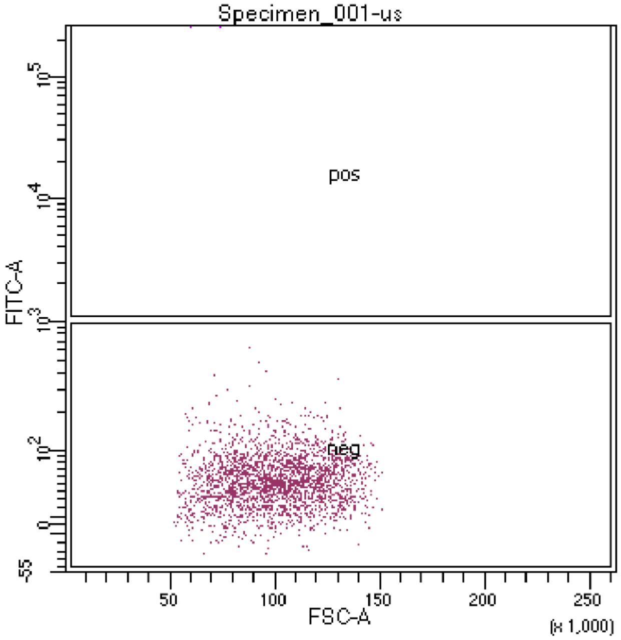

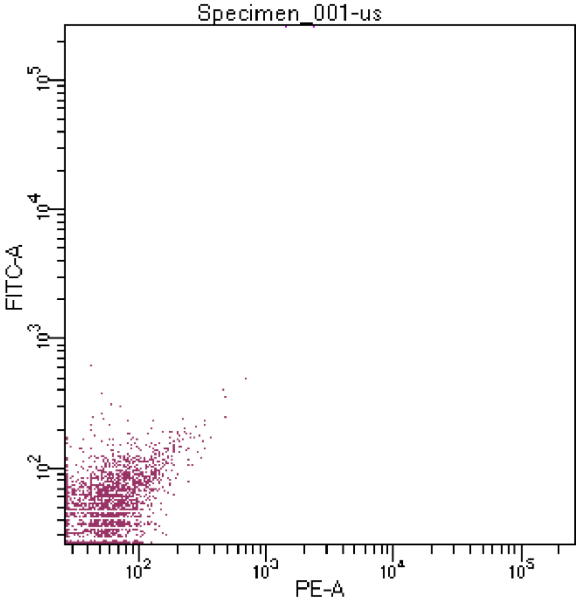

BD FACSDiva 8.0.1

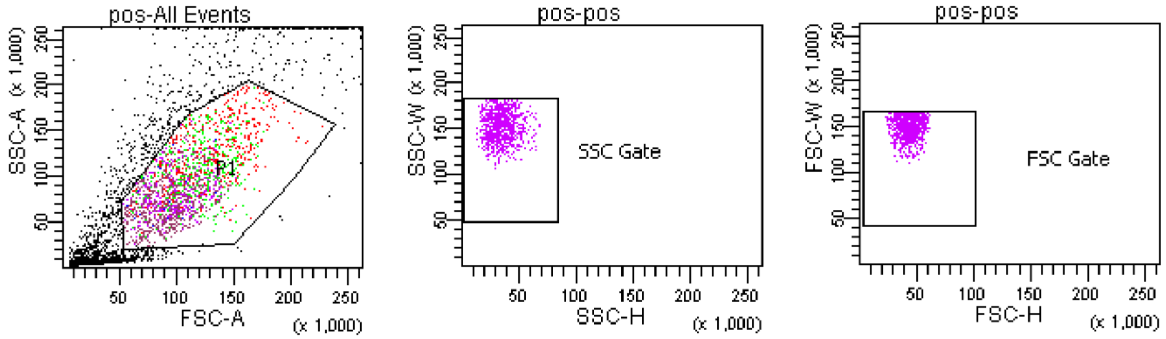

Tube: pos

Population	#Events	%Parent	%Total
All Events	10,000	####	100.0
P1	5,260	52.6	52.6
SSC Gate	3,696	70.3	37.0
FSC Gate	2,383	64.5	23.8
pos	831	34.9	8.3
neg	1,481	62.1	14.8

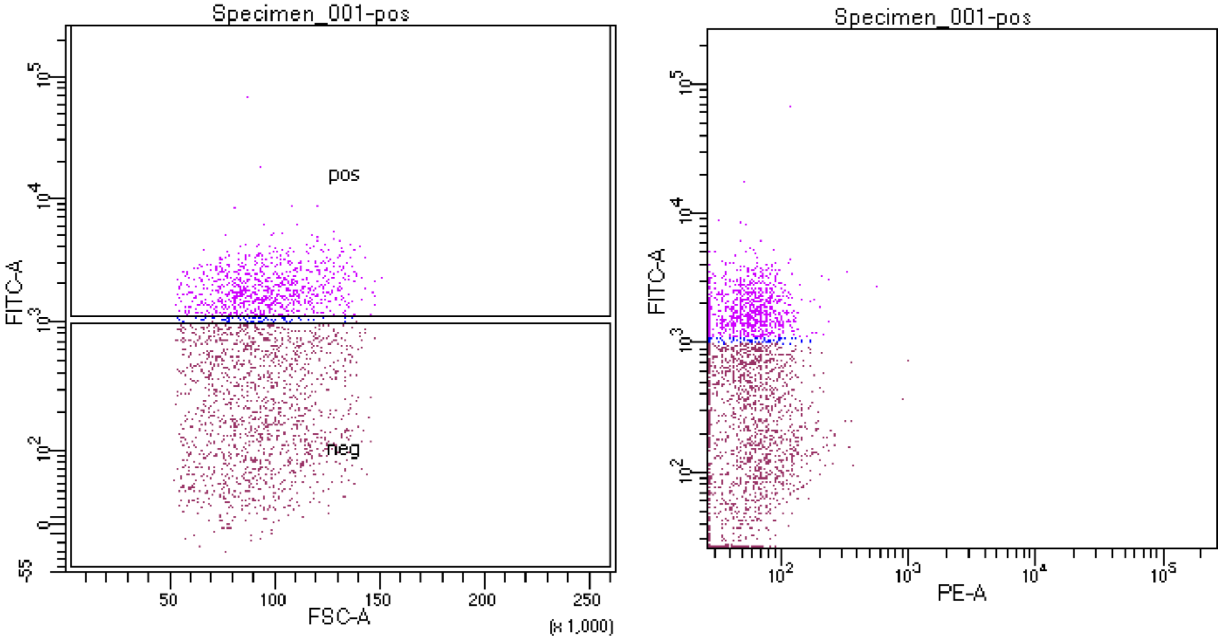

BD FACSDiva 8.0.1

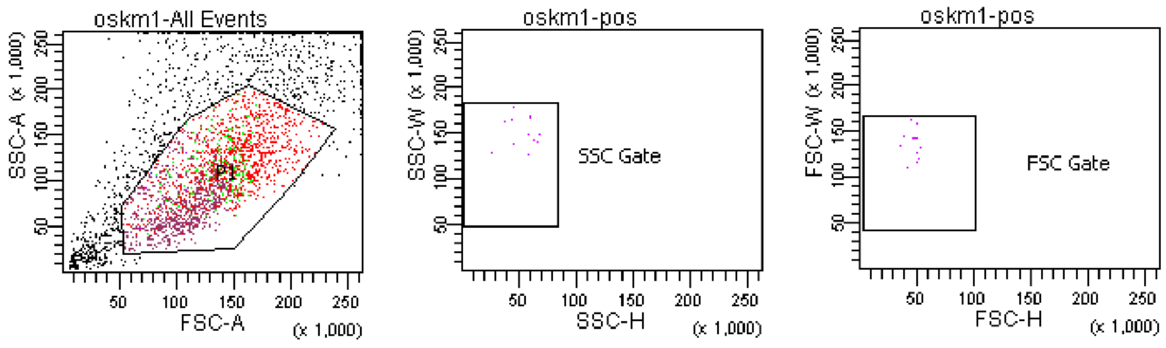

Tube: oskm1

Population	#Events	%Parent	%Total
All Events	10,000	####	100.0
P1	5,506	55.1	55.1
SSC Gate	2,560	46.5	25.6
FSC Gate	1,950	76.2	19.5
pos	12	0.6	0.1
neg	1,934	99.2	19.3

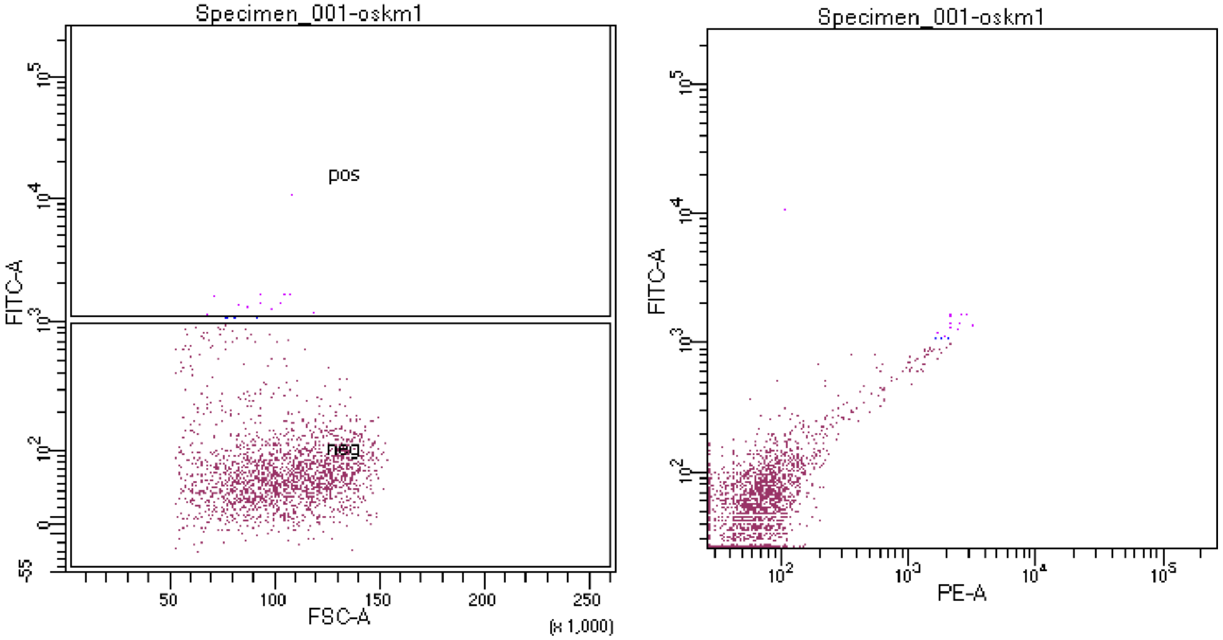

BD FACSDiva 8.0.1

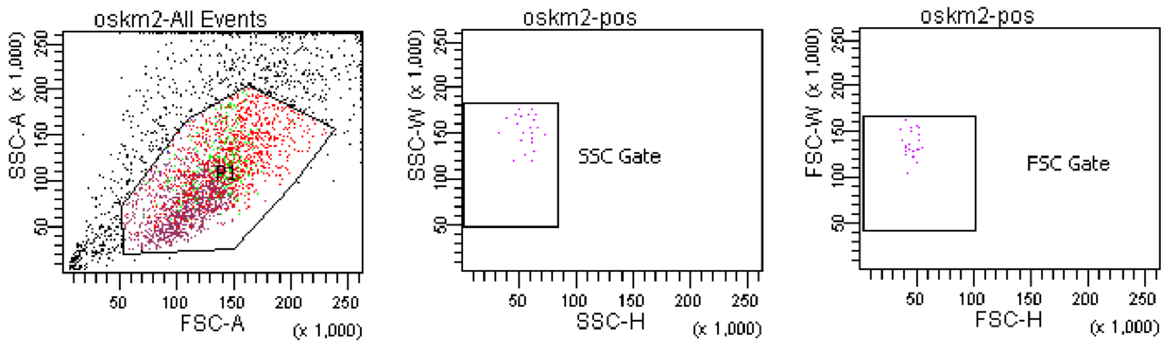

Tube: oskm2

Population	#Events	%Parent	%Total
All Events	10,000	####	100.0
P1	6,247	62.5	62.5
SSC Gate	3,101	49.6	31.0
FSC Gate	2,391	77.1	23.9
pos	23	1.0	0.2
neg	2,365	98.9	23.6

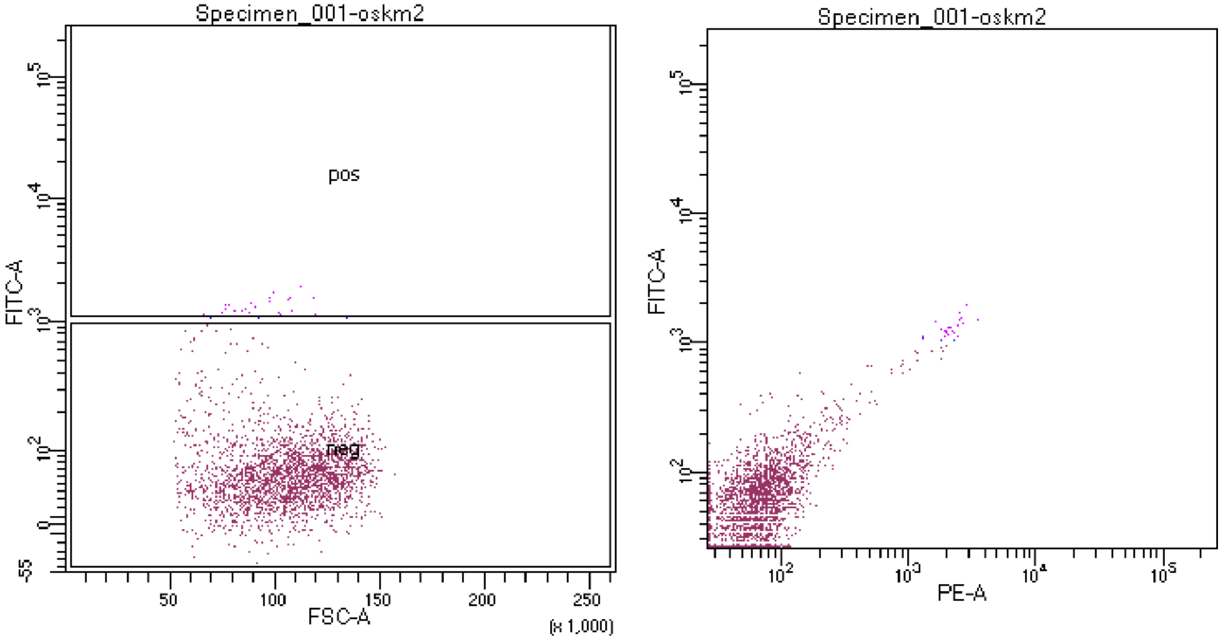

BD FACSDiva 8.0.1

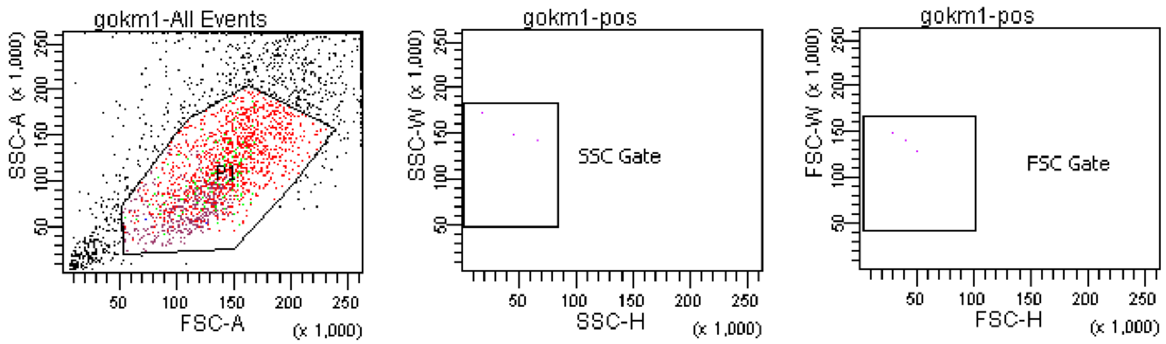

Tube: gokm1

Population	#Events	%Parent	%Total
All Events	10,000	####	100.0
P1	4,904	49.0	49.0
SSC Gate	1,470	30.0	14.7
FSC Gate	1,085	73.8	10.8
pos	3	0.3	0.0
neg	1,075	99.1	10.8

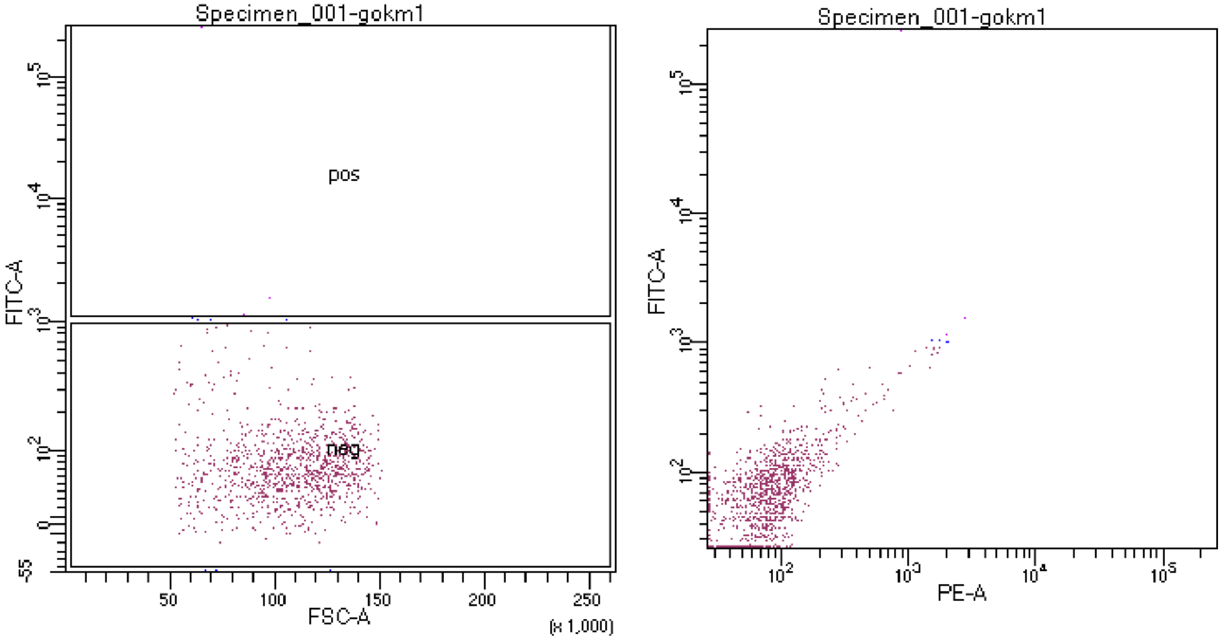

BD FACSDiva 8.0.1

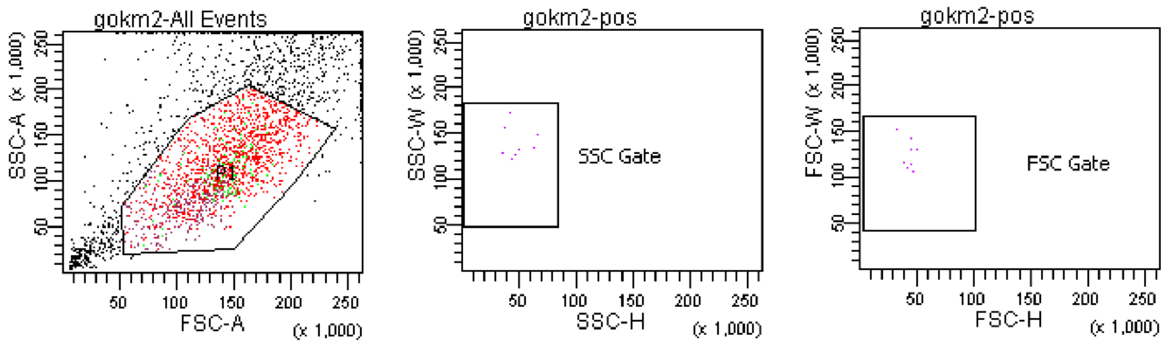

Tube: gokm2

Population	#Events	%Parent	%Total
All Events	10,000	####	100.0
P1	4,714	47.1	47.1
SSC Gate	1,164	24.7	11.6
FSC Gate	837	71.9	8.4
pos	8	1.0	0.1
neg	829	99.0	8.3

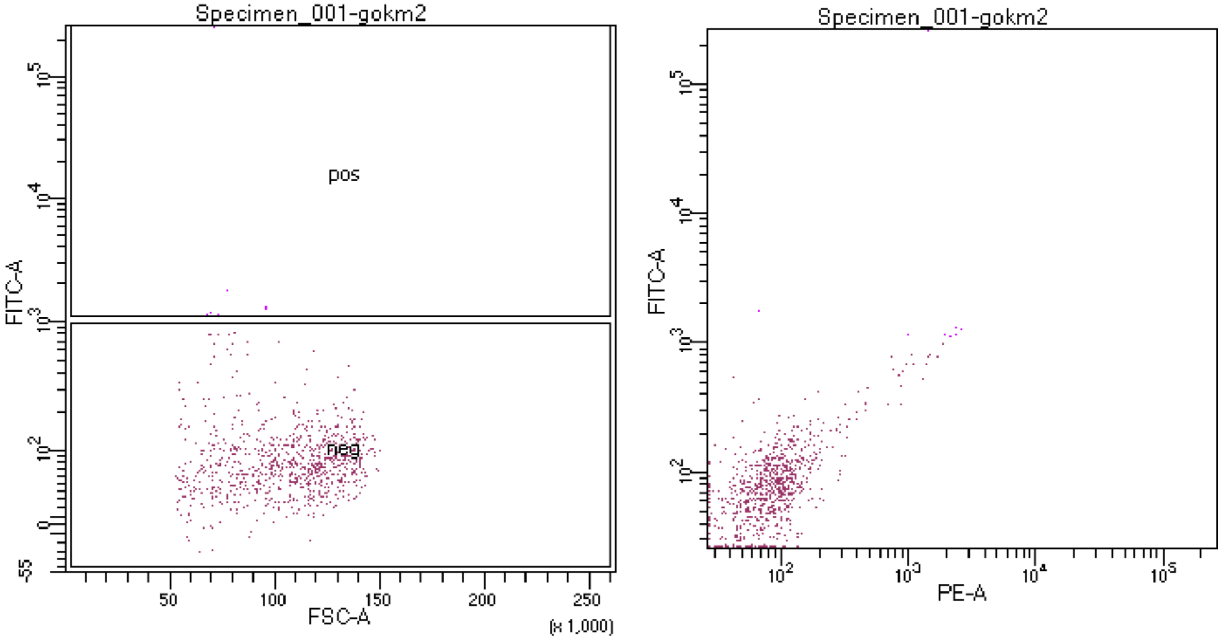

Supplementary Fig. 11. Gating strategy for HLA-A/B/C and TRA-1-60. To remove dead cells, all samples were initially gated using the FSC-A/SSC-A gating to identify the live cell population (below 200 FS Area for beckman coulter and 50 for FACSAria III). To remove cell doublets, single cells were selected by gating forward scatter height vs area. The positively fluorescent cells were gated based on the fluorescent intensity of positive control cells (Fibroblasts for HLA-A/B/C or iPSCs for TRA-1-60) as compared to negative control cells (secondary only and hbdTSCs for HLA-A/B/C or fibroblasts for TRA-1-60). For TRA-1-60, one time point (day 12) is shown but the same parameters were used for all time points.

METHODS

Gene	Application	Primer Sequence (5' --> 3')
GAPDH (intronic)	qPCR analysis of integration into genomic DNA normalization	F: TGGTATCGTGGAAGGACTCA<br>R: TTCAGCTCAGGGATGACCTT
GATA3 (F)	qPCR analysis of integration into genomic DNA	AGCCTGTCCTTTGGACCAC
TFAP2C (F)	qPCR analysis of integration into genomic DNA	AACCCTGGAGACCAGAGTCC
ESRRB (F)	qPCR analysis of integration into genomic DNA	GAAAGCATCTCTGGCTCACC
OCT4 (F)	qPCR analysis of integration into genomic DNA	CTGTCTCCGTCACCACTCTG
SOX2 (F)	qPCR analysis of integration into genomic DNA	GCACACTGCCCCTCTCAC
KLF4 (F)	qPCR analysis of integration into genomic DNA	GACCACCTCGCCTTACACAT
MYC (F)	qPCR analysis of integration into genomic DNA	AGCATACATCCTGTCCGTCC
mSOX2 (F)	qPCR analysis of mouse transgene integration into genomic DNA	GGAGCCCAGCGCCATACCG
FUW plasmid (R)	qPCR analysis of integration into genomic DNA	AGAATACCAGTCAATCTTTCAC
GAPDH	qPCR analysis of mRNA expression normalization	F: CCTCAACGACCACTTTGTCAAG<br>R: TCTTCCTCTTGTGCTCTTGCTG
GATA3 5' UTR<br>(endogenous expression)	qPCR analysis of mRNA expression	F: ACGACCCCTCCAAGATAATTTT<br>R: GTCGGGGGTCGTTGAATGAT
OCT4 3' UTR	qPCR analysis of mRNA expression	F: GGGTTTTTGGGATTAAGTTCTTCA

(endogenous expression)		R: GCCCCCACCCTTTGTGTT
TFAP2C	qPCR analysis of mRNA expression	F: GGTTGAATCTTCCGGCCG<br>R: TCTGCCACTGGTTTACTAGGA
TFAP2A	qPCR analysis of mRNA expression	F: GGACCACCTGGTATTCTGTATTT<br>R: CTGGGCAACAAAGGACTATGA
GATA2	qPCR analysis of mRNA expression	F: GAACCGACCACTCATCAAGC<br>R: TTCTTCATGGTCAGTGGCCT
KRT7	qPCR analysis of mRNA expression	F: AAGAACCAGCGTGCCAAGT<br>R: TCCAGCTCCTCCTGCTTG
TP63	qPCR analysis of mRNA expression	F: AGAAACGAAGATCCCCAGATGA<br>R: CTGTTGCTGTTGCCTGTACGTT
HLA-A	qPCR analysis of mRNA expression	F: GCTCCCACTCCATGAGGTAT<br>R: AGTCTGTGACTGGGCCTTCA
ERVFRD-1	qPCR analysis of mRNA expression	F: AGCCAACAACATTGACACCA<br>R: TTTGAAGGACTACGGCTGCT
CSH1	qPCR analysis of mRNA expression	F: ACTGGGCAGATCCTCAAGC<br>R: GTCATGGTTGTGCGAGTTTG
PSG1	qPCR analysis of mRNA expression	F: CTAACCCACCGGCACAGTAT<br>R: TCGACTGTCATGGATTTGGA
CGB	qPCR analysis of mRNA expression	F: CAGCATCCTATCACCTCCTGGT<br>R: CTGGAACATCTCCATCCTTGGT
SDC1	qPCR analysis of mRNA expression	F: CTATTCCCACGTCTCCAGAACC<br>R: GGACTACAGCCTCTCCCTCCTT

GCM1	qPCR analysis of mRNA expression	F: TGTGAAACTGCCACAGAACG<br>R: GTGTTTGGCATAGGAATCTGG
CHSY1	qPCR analysis of mRNA expression	F: GTCATGACCGCCCAGAAATA<br>R: TCCCAGGAATTGTCTTGGAC
HLA-G	qPCR analysis of mRNA expression	F: TTGGGAAGAGGAGACACGGAACAC<br>R: CTCCTTTGTTCAGCCACATTGGCC
MMP2	qPCR analysis of mRNA expression	F: TGGCACCCATTTACACCTACAC<br>R: ATGTCAGGAGAGGCCCCATAGA
ITGA1	qPCR analysis of mRNA expression	F: GAGCCTATGATTGGAATGGA<br>R: GGTTGTGTTTCGAGGGATTA
ITGA5	qPCR analysis of mRNA expression	F: CAACATCTGTGTGCCTGACC<br>R: CCAGGTACACATGGTTCTGC
KRT18	qPCR analysis of mRNA expression	F: CTGCTGCACCTTGAGTCAGA<br>R: ATGTTCAGCAGGGCCTCATA
CDH1	qPCR analysis of mRNA expression	F: CTCGACACCCGATTCAAAGT<br>R: GGCGTAGACCAAGAAATGGA
OCLN	qPCR analysis of mRNA expression	F: ACAAATGGACCTCTCCTCCA<br>R: ATGGCAATGCACATCACAATA
EPCAM	qPCR analysis of mRNA expression	F: GCAGCTCAGGAAGAATGTGTC<br>R: TGAAGTACACTGGCATTGACG
THY1	qPCR analysis of mRNA expression	F: CCAGAACGTCACAGTGCTCA<br>R: AGGTGTTCTGAGCCAGCAG
ZEB1	qPCR analysis of mRNA expression	F: TTTTCCCATTCTGGCTCCTA

		R: TGGTGATGCTGAAAGAGACG
VIM	qPCR analysis of mRNA expression	F: CCGACACTCCTACAAGATTTAGA<br>R: CAAAGATTTATTGAAGCAGAACC
ACTA2	qPCR analysis of mRNA expression	F: GTGACGAAGCACAGAGCAAA<br>R: TGGTGATGATGCCATGTTCT
hSOX2	qPCR analysis of human-specific mRNA expression	F: TCTTGGCTCCATGGGTTCG<br>R: GGGAGGAAGAGGTAACCACA
mSox2	qPCR analysis of mouse-specific mRNA expression	F: GCTGGGCTCCATGGGCTCT<br>R: GGGAGGAAGAGGTAACCACG
Primer pair A	qPCR analysis of CRISPR/Cas9 indels	F: TGTACAACATGATGGAGACGGA<br>R: GCTTGCTGATCTCCGAGTTG
Primer pair B	qPCR analysis of CRISPR/Cas9 indels	F: GGGCGGCGGCGGCAACTCCACC<br>R: GGGACCACACCATGAAGG
SOX2-gRNA	gRNA designed for generation of indels with pLentiCRISPR system	F: CACCGTGGGCCGCTTGACGCGGTC<br>R: AAACGACCGCGTCAAGCGGCCCAC
NANOG-gRNA	gRNA designed for generation of indels with pLentiCRISPR system	F: CACCGAGTCGGATGCTTCAAAGCA<br>R: AAACTGCTTTGAAGCATCCGACTC
PRDM14-gRNA	gRNA designed for generation of indels with pLentiCRISPR system	F: CACCGACCAGGGCAGATCGTAGAG<br>R: AAACCTCTACGATCTGCCCTGGTC

Supplementary Table 1. primer list
