# Supplementary material for: Pluripotency-independent induction of human trophoblast stem cells from fibroblasts
Source: Nat Commun. 2023 Jun 8;14:3359. doi: 10.1038/s41467-023-39104-1 (PMC10250329; doi:10.1038/s41467-023-39104-1)
Supplement: Supplementary file 2 — Reporting Summary [file 41467_2023_39104_MOESM2_ESM.pdf]

nature portfolio

Corresponding author(s): Yosef Buganim

Last updated by author(s): 04/23/2023

# Reporting Summary

Nature Portfolio wishes to improve the reproducibility of the work that we publish. This form provides structure for consistency and transparency in reporting. For further information on Nature Portfolio policies, see our Editorial Policies and the Editorial Policy Checklist.

## Statistics

For all statistical analyses, confirm that the following items are present in the figure legend, table legend, main text, or Methods section.

| n/a | Confirmed | |
|---|---|---|
| ☐ | ☒ | The exact sample size (*n*) for each experimental group/condition, given as a discrete number and unit of measurement |
| ☐ | ☒ | A statement on whether measurements were taken from distinct samples or whether the same sample was measured repeatedly |
| ☐ | ☒ | The statistical test(s) used AND whether they are one- or two-sided<br>*Only common tests should be described solely by name; describe more complex techniques in the Methods section.* |
| ☒ | ☐ | A description of all covariates tested |
| ☐ | ☒ | A description of any assumptions or corrections, such as tests of normality and adjustment for multiple comparisons |
| ☐ | ☒ | A full description of the statistical parameters including central tendency (e.g. means) or other basic estimates (e.g. regression coefficient) AND variation (e.g. standard deviation) or associated estimates of uncertainty (e.g. confidence intervals) |
| ☐ | ☒ | For null hypothesis testing, the test statistic (e.g. *F*, *t*, *r*) with confidence intervals, effect sizes, degrees of freedom and *P* value noted<br>*Give P values as exact values whenever suitable.* |
| ☒ | ☐ | For Bayesian analysis, information on the choice of priors and Markov chain Monte Carlo settings |
| ☐ | ☒ | For hierarchical and complex designs, identification of the appropriate level for tests and full reporting of outcomes |
| ☐ | ☒ | Estimates of effect sizes (e.g. Cohen's *d*, Pearson's *r*), indicating how they were calculated |

*Our web collection on statistics for biologists contains articles on many of the points above.*

## Software and code

Policy information about availability of computer code

| | |
|---|---|
| Data collection | Bulk RNA seq were collected with Illumina NextSeq 500 platform to generate 75-bp single-end reads. The RRBS libraries were sequenced by Illumina HiSeq 2500 platform. ChIP and ATAC data were obtained using Illumina NextSeq 500 platform. |
| Data analysis | RNA-Seq analysis: Poly-A/T stretches, and Illumina adapters were trimmed from the reads using cutadapt (doi:https://doi.org/10.14806/ej.17.1.200); resulting reads shorter than 30bp were discarded. Reads for each sample were aligned independently to the H. sapiens reference genome GRCh38 using STAR55. Counting proceeded over genes annotated in Ensembl using htseq-count56. Only uniquely mapped reads were used to determine the number of reads that map to each gene (intersection-strict mode). Differential analysis was performed using DESeq257 package in R with the betaPrior, cooksCutoff, and independentFiltering parameters set to False. Raw P values were adjusted for multiple testing using the procedure of Benjamini and Hochberg. Differentially expressed (DE) genes were determined by a p-adj of < 0.05, absolute fold changes > 2, and a count of at least 30 in at least one sample.<br>PCA analysis was done on the 1000 most variable genes between all samples.<br>Additional samples of hiTSC were added to the analysis. The relevant count matrixes were downloaded from3,9. The normalized counts of all samples were adjusted using the limma58 removeBatchEffect function for downstream visualization.<br>201 DE genes specific to hbdTSC with logFC > 6 when compared to both hiPSC and fibroblasts cells were selected, and their expression were plotted in the other samples. The same analysis was done for 289 DE genes specific to hiPSC.<br><br>DNA Methylation analysis: For the analysis of RRBS data, raw reads (FastQ files) were quality-trimmed using Trim Galore (v 0.6.5, default parameters) and aligned to the human genome GRCh38 using BSMAP (v 2.9 59). The methylation ratio of CpGs with sequencing depth of at least 10 reads were computed based on 100bp tiles. Differentially methylated regions (DMR) table obtained from Methylkit (v 1.14.2, DOI: 10.18129/B9.bioc.methylKit) processing of the BAM files yielded by BSMAP alignment. Each table represents the following parameters: Chromosome (chr), start and end coordinates of the methylated region (start, end), strand location (strand), probability value "pvalue", |

adjusted p-value "qvalue", differential methylation score "meth.diff". Only regions with a meth.diff score over 50 or under -50 and a q-value under 1E-5 where considered as differentially methylated (hyper- or hypo-methylated respectively).
For the analysis of direct amplification and sequencing of the ELF5 promoter a FATSTA file that contains ~20 kbp from the human genome GRCh38 upstream of ELF5 (chr11:34496606-34517332) was constructed. An index for the ELF5 FASTA file was constructed using the function "bismark_genome_preparation" from the Bismark (v 0.22.3, 60). Alignment was then performed using bismark --bowtie2 61 and methylation. Finally, methylation information was extracted using the function "bismark_methylation_extractor".

ATAC-Seq and ChIP-Seq analysis: For ATAC-seq, the cutadapt (doi:https://doi.org/10.14806/ej.17.1.200) tool was used to trim adapters using the following sequence: CTGTCTCTTATACACATCT from both reads. Additional 3 bases were trimmed from the end of the read. Reads which were shorter than 25 bases after the trimming were discarded. The trimmed reads were aligned to the H. sapiens reference genome GRCh38 using bowtie61 (version 1.0.0), using the options --maxins 2000, -m 1, best, strata, and -n 1. Reads that were mapped to the mitochondrial chromosome were excluded. Duplicate reads were removed using Picard (https://broadinstitute.github.io/picard/) (version 2.3.0) MarkDuplicates command. Peaks were called using MACS262 with --nomodel --shift -100 --extsize 200 parameters. Peaks from unknown contigs and blacklist regions were removed from the analysis.
For ChIP-seq, H3K4me2 data of hESC was downloaded from GSE16256.
Illumina adapters were trimmed from the reads using cutadapt (doi:https://doi.org/10.14806/ej.17.1.200). Reads which were shorter than 25 bases after the trimming were discarded. The trimmed reads were aligned to the H. sapiens reference genome GRCh38 using bowtie61 (version 1.0.0), using the options -m 1 --phred33-quals -5 0 -3 0 --best -n 1 -l 28 --strata. Reads that were mapped to the mitochondrial chromosome were excluded. Peaks were called using MACS262 with --nomodel --broad parameters. Peaks from unknown contigs and blacklist regions were removed from the analysis.
Peaks from all samples were merged using bedtools63 merge (version 2.29.2) command. R csaw package64 was used to count reads on the merged peaks. EdgeR65 package in R was used for normalization and differential peak analysis. Peaks were defined specifically for each group of cells and the R package (VennDiagram) was used to construct all Venn diagrams. Differential peaks were determined by FDR < 0.05 and absolute log2 fold changes > 1. For GOKM and OSKM differential ATAC-seq analysis, DA peaks were determined with the same criteria as above, with the additional filter of unique peaks (peaks that were called as peaks using MACS2 only in one of the samples).
Association between peaks and genes was done using GREAT66 with the Basal plus extension option with distal up to 150kb. Extracted genes were then intersected with a specific set of genes that were differentially expressed in hbdTSCs and not in fibroblasts or hESCs, or set of genes that were differentially expressed in hESCs and not in fibroblasts or hbdTSCs.
Motif analysis on specific sets of peaks was done using Homer67 findMotifsGenome.pl with --size given parameter. The summits of the peaks were extended +-100bp and these intervals were used for the motif analysis.

Flow cytometry analysis of HLA class I and TRA-1-60 expression: HLA class I stained cells were analyzed by Beckman Coulter (Gallios) flow cytometer using the Kaluza Software (V 1.0.14029.14028). TRA-1-60 stained cells were sorted using FACSAria III (BD Biosciences) and analyzed using BD FACSDiva (V 8.0.1).

For manuscripts utilizing custom algorithms or software that are central to the research but not yet described in published literature, software must be made available to editors and reviewers. We strongly encourage code deposition in a community repository (e.g. GitHub). See the Nature Portfolio guidelines for submitting code & software for further information.

# Data

Policy information about availability of data

All manuscripts must include a data availability statement. This statement should provide the following information, where applicable:

- Accession codes, unique identifiers, or web links for publicly available datasets
- A description of any restrictions on data availability
- For clinical datasets or third party data, please ensure that the statement adheres to our policy

All RNA-seq, RRBS, ATAC-seq and ChIP-seq data were deposited in the Gene Expression Omnibus database (GEO) under accession number GSE182017 (https://www.ncbi.nlm.nih.gov/geo/query/acc.cgi?acc=GSE182017). All analyses used UCSC hg38 human reference genome [http://genome-euro.ucsc.edu/cgi-bin/hgTracks?db=hg38]. The figures that are associated with the raw data files are: Figs 2a,b,d, 3a-n, 4a-d and 4f, 5a-j, S2a-e, S3a-j, S4a-k and S5a-k, S9a-h and S10. Remaining data are provided within the Article, Supplementary Information. Source Data are provided with this paper.

# Human research participants

Policy information about studies involving human research participants and Sex and Gender in Research.

| | |
|---|---|
| Reporting on sex and gender | Differences in the sex of trophoblast cells are known to impact placental function (PMID: 36114567). Therefore, we deemed it important to confirm our ability to derive bona-fide trophoblast stem cells from both male and female somatic cells. Thus, this study included both male (XY) and female (XX) primary human fibroblasts, and hiTSC identity was confirmed for each line individually. the Sex of cof the cells was confirmed using karyotype analysis (Figure S6A, except for the GM2 line), and additionally confirmed through RNAseq performed on all primary cell cultures from which hiTSCs were derived. |
| Population characteristics | N/A |
| Recruitment | Human cell lines were selected based on availability through our institution and collaborators. Though differences in the sex of trophoblast cells are known to impact placental function (PMID: 36114567), these differences were not explored in depth in this paper, as our focus was on the ability to derive bona-fide trophoblast stem cells from both male and female somatic cells. Therefore, selection of the specific male and female lines are not expected to significantly impact our results. |
| Ethics oversight | All embryo (hESCs and hbdTSCs) or patient-derived primary cells (KEN) were isolated in compliance with protocols approved by the Ethics Committee of Shaare Zedek Medical Center (IRB 87/07 and IRB 88/11).<br>Embryo donations were carried out under the strict regulation of the National Ethics Committee (Israel Health Ministry) and |

the NIH (which is in accordance with the ISSCR guidelines). The donated embryos are byproducts of PGD treatments and would otherwise have been destroyed. Complete separation was maintained between the individual who approached the patients and received informed consent for donation (genetic counselor), the attending physician (IVF gynecologist) and the researcher. While obtaining informed consent, the general aim of the research was explained to the patients. The patients were approached for donation only once and not every IVF cycle, to ensure that there is no connection between the signing of the informed consent form and the medical/diagnostic treatment. There was no monetary compensation for the embryo donation.

Note that full information on the approval of the study protocol must also be provided in the manuscript.

# Field-specific reporting

Please select the one below that is the best fit for your research. If you are not sure, read the appropriate sections before making your selection.

☒ Life sciences ☐ Behavioural & social sciences ☐ Ecological, evolutionary & environmental sciences

For a reference copy of the document with all sections, see nature.com/documents/nr-reporting-summary-flat.pdf

# Life sciences study design

All studies must disclose on these points even when the disclosure is negative.

| | |
|---|---|
| Sample size | No minimum sample size was calculated prior to start of experiments, as the bulk of our work was a qualitative assessment of hiTSC identity and function (i.e. marker expression, morphology, differentiation ability etc), rather than a quantitative comparison. RNA-seq, ATAC-seq, ChIP-seq and RRBS experiment sizes were determined in accordance with common practice (PMID: 29220667, PMID: 26412562, PMID: 31031139, PMID: 35715410) and included 2 (RNA-seq, ATAC-seq, ChIP-seq) or 3 (RRBS) biological replicates per line. For ATAC-seq, 50,000 cells were used per replicate. For ChIP-seq, ~10 million cells were used per replicate. |
| Data exclusions | In the RRBS analysis not all examined hiTSC clones were included in the final figure due to space constraints. The samples that were excluded demonstrated a comparable pattern of methylation to the other five hiTSC clones that were included in the final figure. Data available upon request. |
| Replication | Detailed descriptions of replications may be found in the figure legends and methods section. All hiTSC characterization experiments were repeated on three separate cell lines, as described in the manuscript. Culture and reprogramming methods were independently replicated by at least 5 scientists. Transcriptome and DNA methylation profiling were based on 2 (RNA-seq) or 3 (RRBS) biological replicates from at least 5 independent hiTSC clones as described in the text. Chromatin accessibility (ATAC-seq) and chromatin activity (ChIP-seq) for the parental cells, induced cells at day 3 of reprogramming and the final cells were performed on two biological duplicates. |
| Randomization | No randomization methods were utilized, as the majority of experiments were cell-culture based. When animals were used (subcutaneous injection into NOD/SCID mice), they were selected randomly for treatment. |
| Blinding | The investigators were not blinded during data collection and analysis, as is common practice for experiments in this work (PMID: 30487605). |

# Reporting for specific materials, systems and methods

We require information from authors about some types of materials, experimental systems and methods used in many studies. Here, indicate whether each material, system or method listed is relevant to your study. If you are not sure if a list item applies to your research, read the appropriate section before selecting a response.

## Materials & experimental systems

| n/a | Involved in the study |
|---|---|
| ☐ | ☒ Antibodies |
| ☐ | ☒ Eukaryotic cell lines |
| ☒ | ☐ Palaeontology and archaeology |
| ☐ | ☒ Animals and other organisms |
| ☒ | ☐ Clinical data |
| ☒ | ☐ Dual use research of concern |

## Methods

| n/a | Involved in the study |
|---|---|
| ☐ | ☒ ChIP-seq |
| ☐ | ☒ Flow cytometry |
| ☒ | ☐ MRI-based neuroimaging |

## Antibodies

| | |
|---|---|
| Antibodies used | Immunofluorescence Staining (1:200 dilution) , Flow Cytometry (1:300 dilution) and Immunohistochemistry (IHC, dilution specified when relevant):<br>GATA3 (Abcam, Cat#ab106625, polyclonal, Lot#GR3190040-10)<br>KRT7 (Abcam, Cat#ab215855, clone#KRT7/760, Lot#GR3230957-1, dilution 1:1000 for IHC)<br>Ki67 (Abcam, Cat#ab15580, polyclonal, Lot#GR3281726-1) |

GATA2 (Abcam, Cat#ab173817, polyclonal, Lot#GR302442-5)
CSH1 (Abcam, Cat#ab15554, polyclonal, Lot#GR3214399-1, dilution 1:100 for IHC)
HLA-G (Abcam, Cat#ab52455, clone#4H84, Lot#GR3343952-4, dilution 1:100 for IHC)
H3K4me2 (Millipore, Cat#07-030, polyclonal, Lot#3117301)
SOX2 (Abcam, Cat#ab97959, polyclonal, Lot#GR3246887-2)
OCT4 (Abcam, Cat#ab19857, polyclonal, Lot#GR3198906-7)
SDC1 (Abcam, Cat#ab128936, clone#EPR6454, Lot#GR3233756-2)
HLA class I - W6/32 (Abcam, Cat#ab22432, clone#W6/32, Lot#GR3247518-3)
TRA-1-60 (Abcam, Cat#ab16288, clone#TRA-1-60, Lot#GR3187999-10)
TFAP2C (SCBT, Cat#sc-12762, clone#6E4/4, Lot#F0916)
KRT18 (SCBT, Cat#sc-51582, clone#C-04, Lot#K0409)
CDH1 (SCBT, Cat#sc-7870, clone#H-108, Lot#L0910)
ITGA5 (Abcam, Cat#ab150361, clone#EPR7854, Lot#GR105933-76)
VIM (CST, Cat#5741S, Lot#B)
Secondary Antibodies (1:500 dilution):
AlexaFluor 594 donkey anti-mouse IgG (Abcam, Cat#ab150112, Lot#GR3218021-1)
AlexaFluor 488 donkey anti-rabbit IgG (Abcam, Cat#ab150065, Lot#GR3225143-2)
AlexaFluor 488 donkey anti-mouse IgG (Invitrogen, Cat#A21202, Lot#853504)

ChIP-seq:
H3K4me2 (Millipore, Cat#07-030, polyclonal, Lot#3117301, 2microgram/reaction)

Validation

Appropriate positive and negative controls were used in immunofluorescence staining and flow cytometry, including blastocyst-derived hTSCs and parental line human fibroblasts, respectively. In flow cytometry experiments, appropriate positive and negative controls as well as negative control staining with secondary antibody only were utilized.
Additionally, all antibodies used were commercially obtained and validated by suppliers (specific validation statements may be procured using Cat# and Lot# on manufacturer's website).

## Eukaryotic cell lines

Policy information about cell lines and Sex and Gender in Research

Cell line source(s)

Human primary fibroblasts (KEN, foreskin; Helsinki), (PCS-201-012, foreskin; ATCC), (GM25432, adult female skin; Coreill), hESCs (gift from Dr. Rachel Eiges), MDA-MB-231 (HTB-26, breast cancer; ATCC), 293T (CRL-3216, embryonic human kidney; ATCC), hbdTSCs (blastocyst-derived; Helsinki). All embryo (hESCs and hbdTSCs) or patient-derived primary cells (KEN) were isolated in compliance with protocols approved by the Ethic Committee of Shaare Zedek Medical Center (IRB 87/07 and IRB 88/11)

Authentication

Fibroblast lines and hiPSCs/hESC lines were confirmed using characteristic marker expression and RNA-seq expression data as described in the manuscript. hbdTSC lines were additionally confirmed to match previously published criteria for human trophoblast, including HLA expression profile and marker expression with immunostaining and cDNA qPCR (Lee et al., Stem Cell Reports 2016). Other cell lines (MDA-MB-231, 293T) were obtained through ATCC with a certificate of analysis.

Mycoplasma contamination

Cell lines were regularly tested and were mycoplasma negative.

Commonly misidentified lines
(See ICLAC register)

No commonly misidentified cell lines were used in this work.

## Animals and other research organisms

Policy information about studies involving animals; ARRIVE guidelines recommended for reporting animal research, and Sex and Gender in Research

Laboratory animals

Mouse (Mus musculus) NOD/SCID male, ~12 weeks of age were used.
The animals were housed in the Ein-Karem Faculty of Medicine and Hadassah Medical Center Animal House and all housing conditions were overseen by the Hebrew University Authority for Biological and Biomedical Models (ABBM).
Temperature and humidity were kept within the limits required by the ABBM standard operating procedures (30%-70% humidity, temp 20C +/- 2C). These parameters are measured and documented on a daily basis.
Controlled lighting is provided to ensure appropriate light/dark cycles according to the biological requirements of the animals and to provide a satisfactory working environment, generally 12:12 hours.

Wild animals

No wild animals were used.

Reporting on sex

In our experiments we used exclusively male NOD/SCID mice for subcutaneous injection of hiTSCs and controls, as we wanted to exclude the possibility of detection of endogenous mouse chorionic gonadotropin in our hCG assays.

Field-collected samples

No field-collected samples were used.

Ethics oversight

The joint ethics committee (IACUC) of the Hebrew University and Hadassah Medical Center approved the study protocol for animal welfare. The Hebrew University is an AAALAC international accredited institute.

Note that full information on the approval of the study protocol must also be provided in the manuscript.

# ChIP-seq

## Data deposition

☒ Confirm that both raw and final processed data have been deposited in a public database such as GEO.

☒ Confirm that you have deposited or provided access to graph files (e.g. BED files) for the called peaks.

| | |
|---|---|
| Data access links<br>*May remain private before publication.* | GSE182017, SuperSeries, private<br>GSE182012 [ATAC-Seq]<br>GSE182013 [ChIP-Seq]<br>GSE182014 [RNA-Seq]<br>GSE182015 [RRBS]<br><br>https://www.ncbi.nlm.nih.gov/geo/query/acc.cgi?acc=GSE182017<br>Token for reviewers: kfkneeiuxbmvxsn |
| Files in database submission | GSM5516199 H3K4me2_hbdTSC_E2_rep1<br>GSM5516200 H3K4me2_hbdTSC_E2_rep2<br>GSM5516201 H3K4me2_KEN_hFF_rep1<br>GSM5516202 H3K4me2_KEN_hFF_rep2<br>GSM5516203 H3K4me2_GOKM_72h_KEN_hFF_rep1<br>GSM5516204 H3K4me2_GOKM_72h_KEN_hFF_rep2<br>GSM5516205 H3K4me2_OSKM_72h_KEN_hFF_rep1<br>GSM5516206 H3K4me2_OSKM_72h_KEN_hFF_rep2 |
| Genome browser session<br>(e.g. UCSC) | N/A |

## Methodology

| | |
|---|---|
| Replicates | ChIP-seq analysis revealed similarity/dissimilarity in deposition of H3K4me2 histone mark between human blastocyst-derived trophoblast stem cells (hbdTSCs), parental fibroblasts (KEN) and pluripotent stem cells (hESCs) compared with fibroblasts which underwent 3 days of ectopic expression of GATA3, OCT4, KLF4, MYC (GOKM) or OCT4, SOX2, KLF4 and MYC (OSKM). The analysis included 2 biological replicates of each line/condition. The Pearson correlation is ~0.97 between replicates. |
| Sequencing depth | ChIPseq read length : 43 bp; paired end.<br>H3K4me2_bdhTSC_E2_rep1 - total reads: 45,501,752, aligned reads: 43,032,913<br>H3K4me2_bdhTSC_E2_rep2 - total reads: 61,587,065, aligned reads: 58,955,375<br>H3K4me2_Ken_hFF_rep1 - total reads: 48,362,971, aligned reads: 46,368,540<br>H3K4me2_Ken_hFF_rep2 - total reads: 49,594,482, aligned reads: 47,780,619<br>H3K4me2_GOKM_72h_Ken_hFF_rep1 - total reads: 43,109,767, aligned reads: 41,697,460<br>H3K4me2_GOKM_72h_Ken_hFF_rep2 - total reads: 46,730,919, aligned reads: 44,861,144<br>H3K4me2_OSKM_72h_Ken_hFF_rep1 - total reads: 37,088,984, aligned reads: 35,308,982<br>H3K4me2_OSKM_72h_Ken_hFF_rep2 - total reads: 85,991,294, aligned reads: 83,396,442 |
| Antibodies | H3K4me2 (Millipore, Cat#07-030, polyclonal, Lot#3117301, 2microgram/reaction) |
| Peak calling parameters | macs2 callpeak -t $bam_file --broad -f BAMPE -g hs -q 0.05 |
| Data quality | Read dense regions P < 0.001. Significant correlation between H3K4me2 rich regions and promoters of multiple key markers for the ESCs, hbdTSCs and hFFs states. |
| Software | Seqmonk, R/Bioconductor packages |

# Flow Cytometry

## Plots

Confirm that:

☒ The axis labels state the marker and fluorochrome used (e.g. CD4-FITC).

☒ The axis scales are clearly visible. Include numbers along axes only for bottom left plot of group (a 'group' is an analysis of identical markers).

☒ All plots are contour plots with outliers or pseudocolor plots.

☒ A numerical value for number of cells or percentage (with statistics) is provided.

## Methodology

| | |
|---|---|
| Sample preparation | For flow cytometry analysis, cells were trypsinized and blocked for ten minutes in incubation buffer containing 0.5% bovine serum albumin (Sigma Aldrich) in PBS. Then, cells were centrifuged and resuspended in incubation buffer with primary |

antibody for 1 hour on ice. Cells were then washed with incubation buffer and incubated for 30 minutes with relevant (Alexa) secondary antibody on ice, after which cells were washed, resuspended in incubation buffer, filtered through mesh and analyzed.

| | |
|---|---|
| Instrument | HLA class I stained cells were analyzed by Gallios Flow Cytometer (Beckman Coulter). TRA-1-60 stained cells were sorted using FACSAria III (BD Biosciences). |
| Software | Results of HLA class I stained cells were analyzed using Kaluza Software. Results of TRA-1-60 stained cells were analyzed using FACSDiva software. |
| Cell population abundance | HLA class I stained cells were not sorted.<br>TRA-1-60 positively stained cells were quantitatively assessed using FACSDiva software and subsequently seeded in hPSC culture conditions, after which the PSC colonies which emerged were quantified. Results are shown in Figure S7G. A low purity of the post-sort cells was expected, as the threshold for determination of positive cells was intentionally set to capture even weakly positive cells (see rationale in manuscript). |
| Gating strategy | For flow cytometry analysis of HLA Class I and TRA-1-60 expression experiments, cell debris was excluded using FSC vs SSC gate, while aggregates were excluded via FSC-H vs FSC-W. For sorting of TRA-1-60 positive cells, standard gating settings commonly utilized at the core facility of The Hebrew University-Hadassah Medical School were used. Positive and negative control cell samples as well as unstained controls (secondary antibody only) were used to set appropriate gates. |

☒ Tick this box to confirm that a figure exemplifying the gating strategy is provided in the Supplementary Information.
